# Biomarker Selection for Adaptive Systems

**Published:** 2024-08-12

**Authors:** Joshua Pickard, Cooper Stansbury, Amit Surana, Lindsey Muir, Anthony Bloch, Indika Rajapakse

**Affiliations:** 1 Department of Computational Medicine & Bioinformatics, University of Michigan, Ann Arbor, MI 48109; 2 RTX Technology Research Center, East Hartford, CT 06108; 3 Department of Mathematics, University of Michigan, Ann Arbor, MI 48109

## Abstract

Biomarkers enable objective monitoring of a given cell or state in a biological system and are widely used in research, biomanufacturing, and clinical practice. However, identifying appropriate biomarkers that are both robustly measurable and capture a state accurately remains challenging. We present a framework for biomarker identification based upon observability guided sensor selection. Our methods, Dynamic Sensor Selection (DSS) and Structure-Guided Sensor Selection (SGSS), utilize temporal models and experimental data, offering a template for applying observability theory to data from biological systems. Unlike conventional methods that assume well-known, fixed dynamics, DSS adaptively select biomarkers or sensors that maximize observability while accounting for the time-varying nature of biological systems. Additionally, SGSS incorporates structural information and diverse data to identify sensors which are resilient against inaccuracies in our model of the underlying system. We validate our approaches by performing estimation on high dimensional systems derived from temporal gene expression data from partial observations. Our algorithms reliably identify known biomarkers and uncover new ones within our datasets. Additionally, integrating chromosome conformation and gene expression data addresses noise and uncertainty, enhancing the reliability of our biomarker selection approach for the genome.

## Introduction

Biomarkers are measurable molecular features that report on the status of a biological system. Biomarkers in clinical settings aid in diagnosis, prediction (response to a therapy, disease risk), prognosis (association with disease outcome), monitoring, and quantifying therapeutic response [14]. However, even well-studied and widely used biomarkers have limitations. For example, the optimal target range for long-term blood sugar levels remains contested [25], but additional measures such as BMI and cholesterol clarify the clinical picture.

Across research and clinical practice, single biomarkers evaluated out of context can be mis-leading [14], while an integrated set of biomarkers provides more effective monitoring, including measuring both desired and undesired effects of an intervention. The decreasing cost of next generation and third generation sequencing and increased availability of analytics and computational power support transitioning from a single biomarker framework to a multi-biomarker framework [17, 114, 118]. The major challenge of a multi-biomarker framework is the selection of appropriate biomarkers that report on the state of the system across all essential features over time.

We can use the concept of observability to aid biomarker selection. A system is mathematically observable when objective measurements, or sensors, from the system sufficiently determine the state [71, 47]. In our approach the sensors are biomarkers, which are selected to maximize the observability in a dynamic model of a biological system. A systems theory framework offers a route for generalized biomarker selection.

While observability is a classic problem of systems theory [54, 35, 68], many challenges remain in applying input/output models or state space models typical of controls engineering to the study of biological systems [23]. In contrast to many physical systems – such as the pendulum, where the position and velocity capture the state and the equations of motion are known – the dynamics of biological systems must be inferred from incomplete data. The high dimensional nature and low temporal resolution of data gathered in many biological experiments present a further challenge, as these data are not compatible with standard methods for identification and learning dynamics of complex systems [13].

In spite of these challenges, many models for predicting cell trajectories during differentiation, perturbation, and reprogramming have been proposed [100, 95, 66]. Remarkably, the landmark cell reprogramming (controller) experiments of Weintraub [120] and Yamanaka [110] were based on characterizing key biomarker genes (observer) of a target cell type. This approach exemplifies a classic principle of control theory in biological systems: the dual concepts of controllability and observability. Nevertheless, our observability analysis of gene regulation supports the notion that steering and monitoring biological systems are in fact not equivalent problems.

To address these challenges, we introduce a framework for biomarker selection founded on dynamic models of gene regulation. We present two templates for sensor selection: Dynamic Sensor Selection (DSS) and Structure-Guided Sensor Selection (SGSS). We demonstrate their efficacy in identifying biomarkers that optimize the observability of dynamics on gene regulatory networks derived from time-series gene expression datasets. Our focus lies on the linear time variant (LTV) state-space model

(1) x(t+1)=A(t)x(t)y(t)=C(t)x(t).

Here, $\text{x}(t)\in {\mathbb{R}}^{n}$ is the system state, representing, for instance, the expression of each gene as a vector; $\text{A}(t)\in {\mathbb{R}}^{n\times n}$ signifies a state transition matrix, akin to a gene regulatory network; $\text{C}(t)\in {\mathbb{R}}^{p(t)\times n}$ stands as the sensor or measurement matrix, dictating our data collection process, so that $\text{y}(t)\in {\mathbb{R}}^{p(t)}$ denotes our measurements or data (where *p*(*t*) ≪ *n*). If **A**(*t*) is fixed for all *t*, we call the system linear time invariant (LTI). When it is cost-prohibitive to measure the full state at each time, the sensor selection problem involves crafting a measurement matrix (**C**(*t*)) to ensure that the low-dimensional data (**y**(*t*)) gathered throughout time or during an experiment offers the greatest insight into the complete state of the system (**x**(*t*)).

## Results

### Dynamic Sensor Selection

Traditional methods for sensor selections first evaluate each variable as a sensor and then suggest monitoring as many top-ranked sensors as feasible, considering experimental constraints [47]. However, by alternating our sensors at each time step to measure different variables, the estimation of nonlinear and time-varying systems can be improved.

#### Biological Oscillators as a Motivating Example.

As a first example, consider how to best observe a network of coupled oscillators

(2) $$\frac{d\text{x}(t)}{dt}=\text{F}(\text{x}(t),\mu (t))-\text{L}(\text{x}(t))\;\text{y}(t)=\text{C}(t)\text{x}(t).$$

Here, **x** is a vector representing the locations or values of each oscillator, **F** is the dynamics of individual oscillators with internal parameters *μ,* and **L** is the diffusion operator specific to the network structure. Turing’s theory of morphogenesis, Smale’s two cell system, the repressilator, and other higher order motifs exemplify the dynamics of many biological systems described by eq. (2), highlighting the importance of its observation [113, 76, 26, 80, 1].

**Figure 1:**
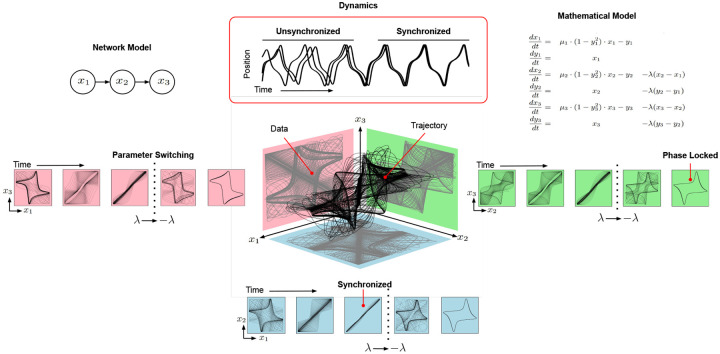
Mathematical Motivation. The trajectory of three coupled Van der Pol oscillators is shown in 3D with each of the possible pairwise projections, obtained by observing only two oscillators, shown in a different plane. As the network shifts from random initial conditions, to synchronization and then phase locking, the amount of information gathered in each plane and utility of the different observations changes. The network model of oscillator coupling, synchronization dynamics, and governing equations of the system are shown at the top.

Figure 1 shows the trajectories of three coupled Van der Pol oscillators. When measuring the state of a pair of oscillators **x**_1_, **x**_2_, or **x**_3_, the observations are 2D projections of the 3D trajectory. With fixed sensors, the question “which oscillators are the best sensors?” is akin to asking “data from with 2D plane enables the best reconstruction of the 3D shape?”

This presupposes that the observed data measure the same two variables at all times. However, the information within each projection changes as the oscillators synchronize and phase lock (fig. 1). As a result, alternating the plane of observed data throughout time provides a clearer picture of the 3D shape and enables better estimation and prediction of the network trajectory.

As a root node (fig. 1), **x**_1_ is a good sensor to monitor the long term behavior of the system [68, 71]. Nevertheless, modification of oscillator connectivity or parameterization before reaching the limiting behavior may necessitate sensor reallocation. As dynamics evolve, the number and distribution of sensors should change as well. For example, synchronized networks require fewer sensors than unsynchronized ones (fig. 1). Similarly, changes in parameterization and connectivity of oscillators necessitates reallocation of sensors (§SI.3.1).

Sensor reallocation is also relevant for monitoring biological processes in living cells. For example, during the cell cycle, whereby a cell replicates to make two new cells, we can monitor four distinct phases called G1: cell growth and copying organelles, S: synthesis or genome replication, G2: reorganize contents to prepare for mitosis, and M: mitosis or physical cell division. Visible, typically fluorescent, sensors have been developed for monitoring the cell cycle in live cells that emit a unique wavelength for each phase. The PIP-FUCCI system, for example, couples expression of three genes with three different colors and enables clear delineation of the G1 to S and S to G2/M transitions [43]. Distinct sensors are used to monitor each transition (SI.fig. 20) [101]. Recently, the introduction of adaptive sampling, which allows for a sequencer to update in real time which genes, cells, or other markers are measured, provides a flexible framework for DSS on high dimensional genomics experiments [17, 114, 118].

#### Maximizing Observability.

We propose two formulations of DSS. Output energy measures the magnitude of the observation **y**(*t*) over time. At time *T*, sensor selection to maximize energy ℰ is formulated

(3) $$\underset{\text{C}(t)}{\text{max}}ℰ\;\text{for}\;\text{all}\;t,\;\text{where}\;ℰ=\sum_{t=0}^{T}\text{y}{\left(t\right)}^{⊤}\text{y}(t).$$

Adapting the approach of [47], eq. (3) is solved through its Lagrange dual form (§SI.3.3). Equation (3) is predicated on the prediction of **y**(*t*), and while this assumption is reasonable in many scenarios the observability Gramian offers a generalized measure of output energy.

To form the discrete-time observability Grammian, let $\Phi \left(t_{2},t_{1}\right)=\text{A}\left(t_{2}\right)\text{A}\left(t_{2}-1\right)\cdots \text{A}\left(t_{1}\right)$ denote the transition matrix from time *t*_1_ to *t*_2_, so that the observability Gramian is

(4) $$G_{o}=\sum_{t=0}^{T}\Phi {\left(t,0\right)}^{⊤}\text{C}{\left(t\right)}^{⊤}\text{C}(t)\Phi (t,0).$$

Using the relation $\text{y}(t)=\text{C}(t)\Phi (0,t){\text{x}}_{0}$, utilizing C(t)Φ(0,t) relaxes the need for a prediction of **y**(*t*). By summing over the inner product of C(t)Φ(0,t) times its transpose, eq. (4) is a direct generalization of the energy ℰ in eq. (3).

In contrast to ℰ*, $G_{o}$* is a matrix rather than a scalar, and several measures of observability derived from $G_{o}$ have been proposed. We consider the problem

(5) $$\underset{\text{C}(t)}{\text{min}}J\left(G_{o}\right)$$

where *J*(·) denotes the trace, logarithm of the determinant, smallest eigenvalue, or rank, each of which provide a different observability measure (§SI.2.2.2). For the trace, eq. (5) is solved with a linear program and can applied to high dimensional systems (§SI.3.4.2). The methodologies of eq. (3) and eq. (5) can handle time-varying sensors, incorporate additional constraints such as SGSS, and support the implementation of scalable algorithms. When compared with alternative sensor selection techniques in table 1, these approaches are versatile.

### Structure Guided Sensor Selection

Sullivan’s maxim “form ever proceeds function” has long established the essence of the structure-function (S-F) causality dilemma. When the system identification problem remains unresolved and the model of function contains errors, SGSS can exploit information in both the structure and function domains to constrain the DSS optimization problems. SGSS considers system geometry and spatial arrangement, leveraging orthogonal experimental methods, to mitigate modeling errors and identify robust sensors.

Knowledge of the structure can aid our estimation and understanding of the dynamics based upon its function. This perspective resonates with approaches in other domains both (1) algorithmically, where methods such as PageRank [37] and the the fast multipole method [99] leverage additional structures to compute on complex systems, and (2) from data, where the S-F relationship has been recognized in the brain [10], gene regulation [96, 24], and community structures [36]. Paired data of the position (S) and effect (F) together, such as genome structure (Hi-C, S) and gene expression (RNAseq, F), is more powerful than either information alone.

Observability can be viewed as either a binary or scalar feature. A well known limitation of the binary Kalman observability test is that all systems in the form of eq. (1) are nearly observable. Apropos of this constraint, in 1974, Lin proposed structural observability, where the sparsity structure of the operators **A** and **C** determine a binary observability condition [68, 70, 71]. In contrast, scalar measures of observability, derived directly from **A**(*t*) and **C**(*t*), provided graded measures of observability (§SI.2.2.1).

While DSS adopts the scalar metric perspective, SGSS departs from the binary view of observability. The structure considered by SGSS is independent of **A**(*t*)’s sparsity but rather based upon external attributes or structures of our system that may not appear in the dynamics. While this notion of structure in SGSS varies from Lin’s usage of the word, the challenge remains the same: despite great experimental advancements over the past half century, system identification and learning the dynamics is not a solved problem for biological systems. Obtaining the data for traditional system identification techniques to be successful is both experimentally challenging and cost-prohibitive. Present methodologies have utilized LTI methods on time-series gene expression signals [47], and SGSS seeks to complement these methods by incorporating readily accessible data pertaining to genome structure.

#### Observability in a Small World.

The tendency to meet strangers with mutual acquaintances is a byproduct of the spatial structures that shape small world networks. For instance, Milgram’s infamous experiment was guided by the geography of individuals from Nebraska to Boston [79]; the Watts-Strogatz (WS) model positions each vertex in a lattice before forming the network [117]; and the small world structure of gene regulatory networks is guided by the 3D organization of chromatin [12]. In each case, the structure guides the formation and dissolution of interactions in the system.

The positioning of nodes on the lattice determines the expected value of each node as a sensor in small world generated with the WS model. We constructed an ensemble of small world networks and evaluated the contribution of each node to the network observability based upon the Gramian. The node contributions to observability on the lattices resembled their average contribution as sensors over all small world networks generated from each lattice (§SI.4.1). Moreover, evenly spacing sensor nodes across the lattice proves an effective strategy for placing sensor nodes on small world networks when the precise small world adjacency structure **A**(*t*) is unknown (§SI.4.1). This suggests that when the precise set or regulatory interactions or network edges of **A**(*t*) is only partially known but the underlying structure is well characterized sensor selection can be guided by the structure.

#### The Human Genome as a Small World.

**Figure 2:**
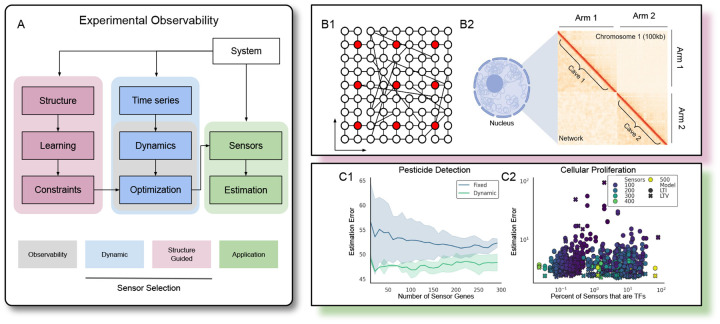
Experimental Framework. **(A)** Determining mathematical observability and performing sensor selection is typically considered within the gray box, where various optimization problems can be formulated based on the dynamics. In the broader experimental framework, we can observe two types of data: structural or functional. From the functional, time-series data, we learn dynamics. From the structural data, we can constrain our optimization. Together, DSS and SGSS should select sensors that are useful to estimate the internal system state. **(B)** Two systems with structural and functional relationships. B1: a small world network is shown where sensors (red) are placed evenly in space; B2: a simple network model (lower left triangle) is fit to Hi-C (upper left triangle) for chromosome 1. **(C)** Estimation results from the Proliferation and SWB25 datasets.

While network models of gene regulation and chromatin architecture have been developed from self organization principles [97] and molecular dynamics simulations [12, 75], quantification of Small World properties of the genome from structural data remains unexplored. We developed a four parameter network model whose adjacency structure qualitatively mirrors Hi-C (fig. 2, §SI.4.3.2). Small World and caveman properties capture the diagonal dominance and block structure characteristic of the fractal globule chromatin architecture and Hi-C data [67, 98]. Based upon our ability to fit networks to Hi-C with relatively few parameters, we proceeded to quantify the Small World Quotient (SWQ) for several Hi-C datasets.

Varying from individual chromosomes to the full genome, the SWQ of Hi-C networks was estimated at several different resolutions, and we observed small world properties in all cases. The SWQ increased with the resolution and size of the Hi-C network and matrix, and the small world properties at multiple resolutions are consistent with the self similar or fractal structures are consistent with classic, multi-scale perspectives of Hi-C [67, 98]. Utilizing Hi-C data collected in parallel with the proliferation and reprogramming datasets, we evaluated the SWQ throughout time; however, in neither dataset did we observe a significant change in the SWQ throughout time. The consistent small world propensity of Hi-C motivates augmenting DSS of gene regulatory networks based upon chromatin structure.

### Applications in Biological Data

We applied DSS and SGSS on a range of data including both genomic and electroencephalogram (EEG) signals (table 2, §SI.5). High dimensional, low frequency gene expression data are at the frontier of observability theory whereas the low dimensional, high frequency EEG signals are a classic problem to study. We used standard approaches to learn LTI and LTV models (§SI.2), and the sensors of each model are assessed based on their ability to estimate the full system state from the sensor measurements (§SI.2.2.3).

#### Proliferation.

To validate our models of gene expression dynamics, we employed established biomarkers from the literature to estimate gene expression during cell proliferation [16] (§SI.5.1). Human fibroblasts were synchronized in terms of both the cell cycle stage and circadian rhythm, offering optimal conditions for learning LTI and LTV models. For sensor selection, we employed the KEGG pathway database, which contains manually curated sets of genes [55] (§SI.5).

Initially, we investigate pathways associated with the cell cycle, such as the Basal Transcription Factors (hsa03022), Cell Cycle (hsa04110), Circadian Rhythm (hsa04710), Circadian Entrainment (hsa04713), and Cellular Senescence (hsa04218) pathways. LTV models had median component wise errors bounded near 10%, which outperformed LTI models when using sensors from all pathways except hsa04713 (SI.fig.19). Although LTV dynamics generally offer superior estimation, we observed that they exhibit decreased robustness due issues such as over fitting and poor conditioning of the observability matrix (§SI.2).

Considering the role of transcription factors (TFs) in determining cell fate and the duality of controllability and observability, we hypothesized that including TFs is essential to forming effective sensor sets. Consistent with this, while hsa04713 contained the third most genes of the sensor pathways considered thus far, it contained no TFs. Repeating the estimation anew with all human KEGG pathways as sensor sets (*n* = 346), we discovered neither the presence of a large number of TFs nor a large sensor set are necessary for good estimation, thereby challenging our hypothesis (fig. 2, §SI.5.6.1). Mathematically, TFs’ effectiveness as controllers but not observers, which is contrary to linear systems theory, is ascribed to the nonlinearity of biological systems. Biologically, TFs’ relatively low expression levels result in low output energy and less variability in their concentrations, necessitating more sensitive observer design and estimation approaches.

We observed a bifurcating behaviour in the estimation procedure. Of the sensors that poorly estimate the initial state, the failed predictions deviate from biologically meaningful values by several orders of magnitude. This improves the interpretability of our approach by offering a clear indicator of failure, even in cases where the true state of the system is unknown.

#### Pesticide Detection.

We build models of the gene regulatory network for *Pseudomonas fluorescens SBW25* and selected biomarkers for malathion detection, a commonly used insecticide [47]. In one model, we learned LTI dynamics (**A**) and time invariant sensors (**C**), and in another model, we learned LTV dynamics (**A**(*t*)) and used DSS (**C**(*t*)). Varying the number of sensors, we assessed the estimation capabilities of each model, and found that LTV dynamics and DSS improved prediction accuracy for reconstructing the expression levels of individual genes. Although eq. (3) and eq. (5) can always be further maximized by adding more sensors, in practice, increasing the number of sensors may not improve estimation, as illustrated in fig. 2.C1.

#### Cellular Reprogramming.

The low efficiency of Weintraub’s famous myogenic reprogramming experiment remains an active challenge in cell reprogramming [120, 69] (§SI.5.2). Monitoring cells throughout reprogramming may offer insight to this issue; however, both formulations of DSS fail to perform well on this system, likely due to the asynchronized and noisy experimental conditions.

We applied SGSS to improve state estimation and increase observability by selecting spatially distributed genes. Based on the hypothesis that colocalized genes are coregulated, we clustered genes according to Hi-C data and constrained DSS to select at most one sensor from each cluster (§SI.4.4). By including constrained selection from Hi-C, the distribution of sensors across chromosomes shifted to mirror the distribution of genes (SI.fig. 21–23). While we cannot measure the spatial proximity of clustered genes, we observed correlation in the expression values of several gene clusters, consistent with the concept of transcription factories. Regardless, the estimation was improved by the Hi-C constrained SGSS. When using few sensors, SGSS reduced the variance and improved estimation accuracy by approximately 25%.

To improve estimation further, we amplified the weak reprogramming signal by sampling genes involved in myogenesis and proliferation (§SI.5.2.1). This targeted dataset provides improved conditions for biomarker identification, counteracting the experimental conditions of reprogramming. Under these conditions, the estimation of the initial state for the reduced data shows median component-wise errors below 15% with all combinations of fixed or dynamic sensors from energy or Gramian based selection.

To close the design → build → test loop for myogenicSignal, we utilized these sensors to estimate the state of the complete reprogramming data. Sensors selected from the reduced data, when optimized for energy, fail to estimate the full data well. This occurs since the high energy genes in the targeted data have low energy in the complete reprogramming signals. However, biomarkers identified via the Gramian on the targeted dataset continue to perform well at estimating the full data. The median component-wise error is improved when applying Gramian selected biomarkers from the targeted data to the full data. Converse of targeted observability [83], where sensors are selected on the full reprogramming time series to observe only the myogenic signal, Gramian based sensor selection identified genes on the reduced data that estimate the full system well.

#### Electroencephalogram Signals.

We employed DSS to rank different sensors observed in EEG signals. The brain’s Small World properties are well-documented, and current research suggest EEGs are observable with few sensors [10, 92]. We ranked the sensors of 64-lead EEG signals based on their contributions to output energy and the observability Gramian. Relative to the genomics data, where synchronized or controlled experiments have low frequency, high dimensional measurements, EEG data are high frequency and low dimensional, and the EEG signals are unsynchronized. Instead EEG signals were partitioned according to different tasks the participants performed, such as opening or closing their eyes, prior to performing sensor selection. The sensor ranking exhibit great variability across different activities, which underscores the utility of DSS, when participants change between tasks, a common occurrence in clinical settings. In this context, the significance of sensors is determined by the participants’ activities or states rather than specific time points from the start of the EEG signals. Consistent with the principles of DSS, transitions between states coincide with variations in the most relevant sensors.

## Discussion

Unbiased biomarker selection offers an accurate and adaptive method to monitor biological systems. This work extends state space and network observability to develop a template for unbiased biomarker selection [54, 35, 68, 71]. We take into account the high-dimensional, non-linear dynamics and limitations on the resolution of data for biological systems and use an initial step of over measuring the system prior to sensor selection. These methods effectively estimated the state of several systems with limited information, improving estimation quality relative to fixed biomarkers. Our algorithms reliably identified known biomarkers and uncovered new ones within our datasets. Additionally, integrating structural and gene expression data effectively addressed noise and uncertainty, enhancing the reliability of our biomarker selection approach.

Time dependent observability is sensible when monitoring episodic systems where sensors can be selected relative to a fixed point in time [47]. For instance, the initial synchronization, addition of transcription factor(s) for reprogramming, or pesticide treatment in the different systems we consider allow time dependent biomarkers to be selected relative to a starting point. For systems with no *a priori* beginning, it is more appropriate to consider local observability [49, 106].

Here we identify transcription factor biomarkers using SGSS based on Hi-C data to improve estimation. Other data types and clustering techniques could also be used, including gene regulatory networks or chromatin accessibility. Thus our approach is versatile and can be adapted for sensor selection in different systems.

The representation of genes as model states is implicit in our state space model. Expanding the state space representation to include other system-level data such as isoforms and chromatin accessibility could enhance these models. In addition, the genomics data used here is at the population level and does not consider single cell dynamics. Integrating single cell data and pseudotime or RNA velocity [112, 61] into our framework would enable biomarker selection in subsets of cells in a complex population.

These modeling approaches, when used with high-quality data for maximizing observability, provide a path to gaining new insights in complex systems. New modeling approaches are especially important for discovery of biomarkers that are useful in humans, given that discovery in animal models does not reliably translate [19]. Here we set a foundation for unbiased selection of high-quality biomarkers that represent essential features of a particular biological system. Our approach can accelerate discovery of biomarkers that enable more effective monitoring and decision-making in biomedical research and clinical settings.

## Supplementary Material

1

## Figures and Tables

**Table 1:** Comparison of Methods.

Method	Criteria	DSS	SGSS	Targeted	Cost
Gramian	ℝ	✓	✓	✗	$𝒪\left(n^{2.5}\right)$
Energy	ℝ	✓	✓	✗	$𝒪\left(kn^{2}\right)$
Structure	0/1	✗	✗	✓	$𝒪\left(e\sqrt{n}\right)$

* *n* is the number of variables, *e* is the number of interactions, and *k* is the number of iterations in an eigenvalue calculation.

**Table 2:** Time series datasets.

Dataset	Dimension	Time Points	Replicates	Ref.
Proliferation	19 235	8	2	[16]
Reprogramming	19 235	15	3	[69]
myogenicSignal	404	15	3	
SBW25	624	9	2	[47]
EEG	64	160	109	[102]

## Significance

A major challenge in research and clinical practice is identification of high quality, reliable biomarkers. Here we develop new methods for unbiased selection of biomarkers in biological systems data. Our framework leverages observability theory for sensor selection while accounting for the dynamics and high-dimensionality of biological systems. Accelerating discovery of biomarkers will enable more effective monitoring and decision-making in biomedical research and clinical settings.
