# Supplementary material for: Dynamic Sensor Selection for Biomarker Discovery
Source: ArXiv. 2025 Jan 17:arXiv:2405.09809v5. Preprint. [Version 5] (PMC11142321)
Supplement: Supplement 1 [file NIHPP2405.09809v5-supplement-1.pdf]

Supplemental Information

The supplemental information is divided into three sections: §4 contains description of the models, datasets, parameters, and methods used in the paper; §5 contains details regarding the DSS optimization methods, and §6 contains supplemental figures referenced throughout the main text.

4 Materials and Methods

4.1 DNA Replication Control

Model Equations. Equation (14) presents the model of DNA replication control originally introduced in [40] and further analyzed in [35]. We note that the dynamics considered in these two studies differ slightly, and we adopt the model and parameterization from [35] to ensure consistency with their observability analysis. The variable R in these equations denotes the $RUM1$ gene of the original model.

$$
\begin{aligned}
\frac{dCdc25}{dt} &= -\frac{K_{CR}\cdot Cdc25}{K_{MCR}+Cdc25}+\frac{K_C\cdot Cdc25\cdot(G2K+\beta\cdot PG2)}{K_{MC}+1-Cdc25}\\
\frac{dG1K}{dt} &= K_5+(K_4+K_{8R})\cdot G1R-K_8\cdot G1K\cdot R-G1K\cdot(V_{6P}\cdot(1-UbE2)+V_6\cdot UbE2)\\
\frac{dG1R}{dt} &= -K_4\cdot G1R-K_{6P}\cdot G1R-K_{8R}\cdot G1R+K_8\cdot G1K\cdot R\\
\frac{dG2K}{dt} &= K_1+(K_4+K_{7R})\cdot G2R+(V_{25P}\cdot(1-Cdc25)+V_{25}\cdot Cdc25)\cdot PG2\\
&\quad -K_K\cdot G2K\cdot R-G2K\cdot(V_{2P}\cdot(1-UbE)+V_2\cdot UbE)-G2K\cdot(V_{WP}\cdot(1-Wee1)+V_W\cdot Wee1)\\
\frac{dG2R}{dt} &= -K_4\cdot G2R-K_{7R}\cdot G2R+K_7\cdot G2K\cdot R-G2R\cdot(K_{2P}+V_{2P}\cdot(1-UbE)+V_2\cdot UbE)\\
\frac{dIE}{dt} &= -\frac{K_{IR}\cdot IE}{K_{MIR}+IE}+\frac{K_I\cdot IEC\cdot(G2K+\beta\cdot PG2)}{K_{MI}+IEC}\\
\frac{d\text{mass}}{dt} &= \mu\cdot\text{mass}\\
\frac{dPG2}{dt} &= -(V_{25P}\cdot(1-Cdc25)+V_{25}\cdot Cdc25)\cdot PG2+K_4\cdot PG2R+K_{7R}\cdot PG2R\\
&\quad -K_7\cdot PG2\cdot R-PG2\cdot(V_{2P}\cdot(1-UbE)+V_2\cdot UbE)+G2K\cdot(V_{WP}\cdot(1-Wee1)+V_W\cdot Wee1)\\
\frac{dPG2R}{dt} &= -K_4\cdot PG2R-K_{7R}\cdot PG2R+K_7\cdot PG2\cdot R-PG2R\cdot(K_{2P}+V_{2P}\cdot(1-UbE)+V_2\cdot UbE)\\
\frac{dR}{dt} &= K_3+K_{6P}\cdot G1R+K_{8R}\cdot G1R+K_{7R}\cdot G2R+K_{7R}\cdot PG2R\\
&\quad -K_4\cdot R-K_8\cdot G1K\cdot R-K_7\cdot G2K\cdot R-K_7\cdot PG2\cdot R\\
&\quad -\frac{K_P\cdot\text{mass}\cdot(CIG1+\alpha\cdot G1K+G2K+\beta\cdot PG2\cdot R)}{K_{MP}+R}+(G2R+PG2R)\cdot(K_{2P}+V_{2P}\cdot(1-UbE)+V_2\cdot UbE)\\
\frac{dUbE}{dt} &= -\frac{K_{UR}\cdot UbE}{K_{MUR}+UbE}+\frac{K_U\cdot IE\cdot(1-UbE)}{K_{MU}+1-UbE}\\
\frac{dUbE2}{dt} &= -\frac{K_{UR2}\cdot UbE2}{K_{MUR2}+UbE2}+\frac{K_{U2}\cdot(G2K+\beta\cdot PG2)\cdot(1-UbE2)}{K_{MU2}+1-UbE2}\\
\frac{dWee1}{dt} &= -\frac{K_W\cdot(G2K+\beta\cdot PG2)+Wee1}{K_{WR}+Wee1}+\frac{K_{WR}\cdot(1-Wee1)}{K_{MWR}+1-Wee1}
\end{aligned}
\tag{14}
$$

Observability in the Synthetic Dataset. We used synthetic data to asses the observability of eq. (14) by validating the rank condition. One thousand state vectors $\mathbf{x}$ were randomly sampled, with each element chosen uniformly at random between 0 and 1. For each state vector $\mathbf{x}$ and for each possible sensor of the 13 state variables, the local, nonlinear observability matrix $\mathcal{O}_i(\mathbf{x})$ was constructed, where i denotes which of the state variables was used as the sensor. To determine the rank of the matrix, the Singular Value Decomposition (SVD) was performed on each matrix $\mathcal{O}_i(\mathbf{x})$ in order to obtain the singular values $\sigma_1 \geq \cdots \geq \sigma_{13}$.

In the strictest sense, the rank of a matrix is defined as the number of its nonzero singular values. However, due to numerical imprecision and noise in data, it is often more practical to consider only those singular values that are significantly different from zero [26, 60]. To asses the rank of only significant singular values, several mechanisms thresholding singular values or determinations of effective rank have been proposed [48, 16]. Here, we define the effective rank of a

matrix by thresholding its singular values, retaining only those that contribute at least ε to the cumulative distribution of singular values. The normalized distribution of singular values, $p_1, \dots, p_k$, is given by

$$p_i = \frac{\sigma_i}{\sum_{j=1}^{k} \sigma_j},$$

where σ_i are the singular values of the matrix. The effective rank is then determined as the largest index i such that $p_i \geq \varepsilon$:

$$\text{Effective rank} = \arg\max_i \{p_i \geq \varepsilon\}.$$

The distributions of normalized singular values and the effective ranks averaged across all thousand simulated states are shown in fig. 4.

The effective rank of $\mathcal{O}_i(\mathbf{x})$ depends on both the sensor variable i and the state variable $\mathbf{x}$. In fig. 4, the effective rank averaged over all thousand vectors $\mathbf{x}$ is shown for each sensors. Some variables, such as "mass", are uniformly poor sensors, as shown by the sharp elbow in their distribution of normalized singular values where only σ_1 has a meaningful contribution. Other sensors, such as *G2R* are in general very good sensors, with high effective ranks on average (thick black line), but elbows in the distribution show that there exist state vectors $\mathbf{x}$ for which those sensors make the system less observable. The variability in the singular values of $\mathcal{O}_i(\mathbf{x})$ indicate that some sensors are better than others for different states $\mathbf{x}$, even if the utility of most sensors is theoretically equivalent.

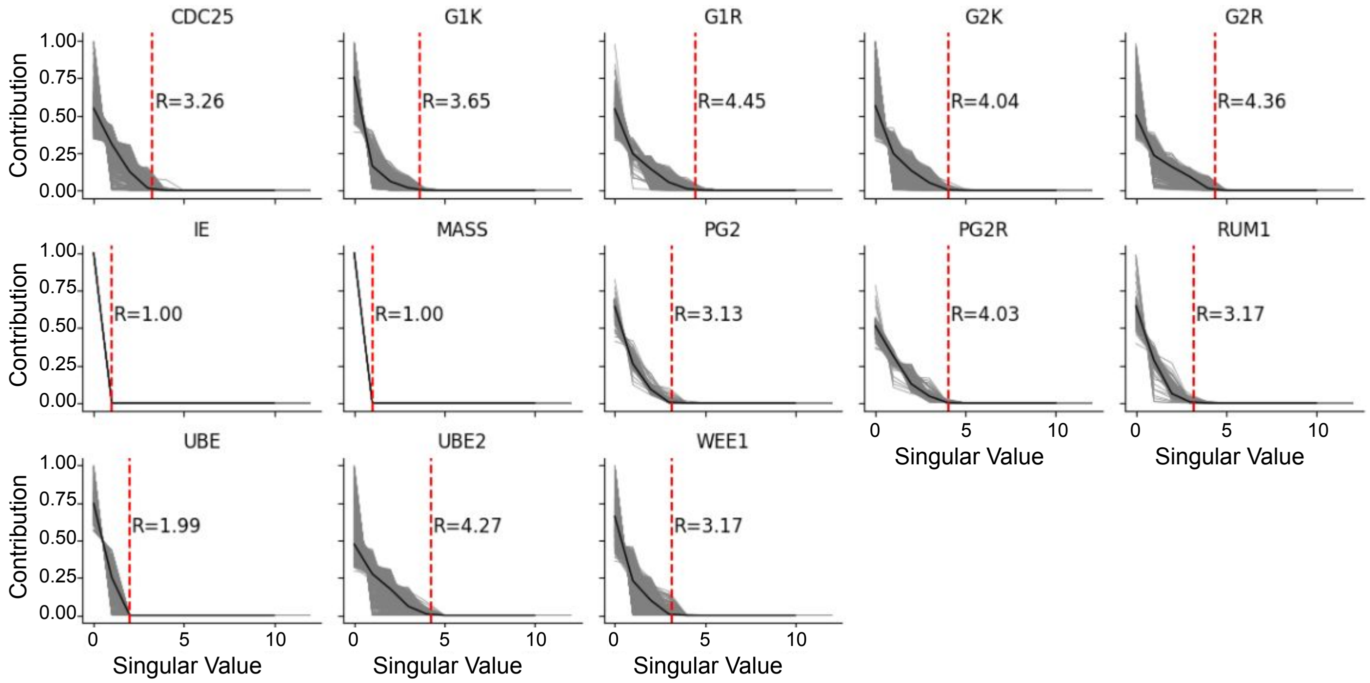

Figure 4: **Effective Rank of Observability Matrices.** The effective rank $\mathbf{R}$ of each sensor is shown using $\varepsilon = 10^{-5}$. Although the system is observable according to Sedoglavik's algorithm [51] as noted by [35], in practice some sensors are better than others.

Greedy Sensor Selection. The Greedy algorithm 1 iteratively selects sensors until the system satisfies the criterion $rank(\mathcal{O}(\mathbf{x})) = n$. The algorithm takes as input observability matrices $\mathcal{O}_i(\mathbf{x})$ where the index i indicates the ith variable in the system is used as a sensor. To select the best sensors, the algorithm examines the performance of different combinations of sensors based on the rank of the observability matrices formed by using groups of sensors togther. The algorithm initializes the desired sensors $\mathcal{D} = \emptyset$ as an empty set, and the candidate sensors $\mathcal{C} = \{1, \dots, n\}$ (`Line 2`). Groups of sensors are evaluated iteratively (loop over `Lines 4-8`), with the rank based stopping criteria (`Line 3`). The utility of adding a new sensor to the desired group $\mathcal{D}$ is evaluated by the marginal increase in the rank of $\mathcal{O}_{\mathcal{D}}(\mathbf{x})$ (`Line 5`), where

$$\mathcal{O}_{\mathcal{D}} = \begin{bmatrix} \mathcal{O}_{d_1}(\mathbf{x}) \\ \dots \\ \mathcal{O}_{d_k}(\mathbf{x}) \end{bmatrix} \text{ where } \mathcal{D} = \{d_1, \dots, d_k\}.$$

After evaluating each candidate sensor, the sensor with the largest marginal increase in the rank of the observability matrix is included in the desired sensor set $\mathcal{D}$ (`Lines 7-8`).

Algorithm 1 Greedy Sensor Selection

Requires: $\mathcal{O}_i(\mathbf{x})$ for $i = 1, \dots, n$
Let $\mathcal{C} = \{1, 2, \dots, n\}$ and $D = \emptyset$
while $\text{rank}(\mathcal{O}_D(\mathbf{x})) < n$ **do**
    for $s \in \mathcal{C} \setminus \mathcal{D}$ **do**
        Compute $\Delta(s) = \text{rank}(\mathcal{O}_{\mathcal{D}\cup\{s\}}(\mathbf{x})) - \text{rank}(\mathcal{O}_{\mathcal{D}}(\mathbf{x}))$
    end for
    Set $s^* = \text{argmax}_{s\in\mathcal{C}\setminus\mathcal{D}}\Delta(s)$
    Set $\mathcal{D} = \mathcal{D} \cup \{s^*\}$
end while
Return The set D.

4.2 Observability of the Andronov-Hopf Oscillator.

Andronov-Hopf Oscillator. The Andronov-Hopf oscillator is named after the physicist Aleksandr Andronov (1901-1952) and mathematician Eberhard Hopf (1902-1983). The Hopf bifurcation, where a parameter controls the transition of a stable point to a limit cycle, is also referred to as the Poincaré-Andronov-Hopf bifurcation to provide attribution to Poincaré and Andronov for their work studying this bifurcation [43, 37, 36, 21].

Empirical Gramians. For nonlinear systems, such as the Andronov-Hopf Oscillator, empirical gramians provide a means to asses the observability measures $\mathcal{M}_1, \mathcal{M}_2$, and $\mathcal{M}_3$ of a system. While the observability Gramian for LTI and LTV systems can be obtained using the Lyapunov equations or the approximation $\mathbf{G}_o \approx \mathcal{O}^\top\mathcal{O}$ such approaches are not directly applicable to nonlinear systems. Instead, the empirical Gramian framework uses simulations to compute observability Gramians [23]. The empirical observability Gramian is defined as:

$$\mathbf{G} = \frac{1}{|S_\mathbf{x}|}\sum_{l=1}^{|S_\mathbf{x}|}\frac{1}{d_l^2}\int_0 \Psi^l(t)\,dt \quad \text{where} \quad \Psi^l_{ij}(t) = (\mathbf{y}(t)_{li} - \bar{\mathbf{y}}_{li})^\top(\mathbf{y}(t)_{li} - \bar{\mathbf{y}}_{li}).$$

The output trajectories $\mathbf{y}(t)_{li}$ correspond go the initial state configuration $\mathbf{x}(0)_{li} = d_l\varepsilon_i + \bar{\mathbf{x}}$. See [28, 25] for additional details.

In our simulations of the Andronov-Hopf oscillator, we used $\varepsilon = 0.01$ and integrated the system for 100 time steps. We assessed $\mathcal{M}_3$, the trace of the observability Gramian, as a measure of how observable the oscillators were with the fixed parameter. Since the system is nonlinear, the observability is a function of both the state vector $\mathbf{x} = \begin{bmatrix} x_1 & x_2 \end{bmatrix}^\top$ and the parameter α. In general, the observability is more sensitive to α than the initial conditions $\mathbf{x}(0)$, but the choice of initial conditions does cause a small amount of variability in the observability of this oscillator, particularly when the system is stable.

4.3 Learning Dynamics from Data

Structure of Time Series Data. The time series data sets considered in the study were structured in the following form (table 2):

$$\mathbf{X} = \begin{bmatrix} | & | & & | & | \\ \mathbf{x}(0) & \mathbf{x}(1) & \dots & \mathbf{x}(T-1) & \mathbf{x}(T) \\ | & | & & | & | \end{bmatrix},$$

where n denotes the number of state variables (genes, neurons, eeg sensors, etc.) and T is the number of time points. Several datasets contained multiple replicates, which correspond to multiple time series matrices $\mathbf{X}$.

**Dataset**	**Dimension**	**Time Points**	**Reps.**	**Ref.**
SBW25	624	9	2	[20]
Reprogramming	19235	15	3	[33]
myogenicSignal	404	15	3	[33]
MiceNeurons	21	1508	3	[57]
EEG	64	160	109	[50]
Proliferation	19235	8	2	[8]

Table 2: Time series datasets.

Dynamic Mode Decomposition (DMD). DMD finds the best linear operator that explains the data $\mathbf{X}$. It solves the minimization:

$$\min_{\mathbf{A}} \|\mathbf{X}^+ - \mathbf{A}\mathbf{X}^-\|_F^2,$$

where the data $\mathbf{X}^-$ and $\mathbf{X}^+$ are matrices containing the first and last $T-1$ time points respectively. See [27] and references therein for additional details.

Data Guided Control. The DGC model for approximating time variant linear systems was proposed by [47] to model the dynamics throughout cell reprogramming. It models the LTV dynamics based on the relationship:

$$\mathbf{A}(t) = \mathbf{I} + \frac{(\mathbf{x}(t+1) - \mathbf{x}(t))\mathbf{x}(t)^\top}{\mathbf{x}(t)^\top \mathbf{x}(t)}.$$

This modeling approach was proposed to model and control similar dynamics of gene expression. The assumption of the DGC model is that gene expression of a population does not change considerably over time. Hence, the state transition matrix $\mathbf{A}$ should be similar to the identity $\mathbf{I}$. From this, the authors of [47] define $\mathbf{A}(t)$ as a rank one perturbation from the identity to fit the data exactly.

5 Dynamic Sensor Selection (DSS)

This section outlines further details regarding the observability optimizations of DSS.

5.1 Output Energy Maximization $\mathcal{M}_2$

Here we provide our method to maximize $\mathcal{M}_2$ based on its Lagrangian dual form. We first discuss how this problem is solved when the sensors $\mathbf{C}$ are fixed for all time and then consider dynamic sensor selection for $\mathcal{M}_2$.

5.1.1 Time Invariant Sensors

The objective is to select sensors $\mathbf{C}$ that maximize the signal or output energy of the system $\mathcal{M}_2$ where

$$\mathcal{M}_2 = \sum_{t=t_0} \mathbf{y}(t)^\top \mathbf{y}(t) = \sum_{t=t_0} \mathbf{x}(0)^\top \mathbf{G}_0(t,t_0)\mathbf{x}(0). \tag{15}$$

Restricting each sensor to measure a single state variable, the optimization is formalized as:

$$\max_{\mathbf{C}} \mathcal{M}_2 \text{ subject to } \mathbf{C}^\top \mathbf{C} = \mathbf{I}, \tag{16}$$

where $\mathbf{I}$ is the identity matrix. Hasnain *et al.* shows that the Lagrangian dual formulation of this problem is

$$\max_{\mathbf{C}} \mathcal{E} + \mathcal{L} \text{ where } \mathcal{L} = \mathbf{tr}((\mathbf{C}\mathbf{C}^\top - \mathbf{I})\mathbf{D}),$$

where $\mathbf{D}$ are the dual variables [20]. Define the Gram matrix $\mathbf{G}$ as

$$\mathbf{G}(t,t_0) = \Phi(t,t_0)\mathbf{x}(0)\mathbf{x}(0)^\top \Phi(t,t_0)^\top.$$

Following eq. 5 of [20], the solution to the optimization problem eq. (16) is achieved as:

$$\frac{\partial(\mathcal{E}+\mathcal{L})}{\partial \mathbf{C}^\top} = 2\mathbf{G}\mathbf{C}^\top - 2\mathbf{C}^\top \mathbf{D} = 0, \text{ such that } \mathbf{G}\mathbf{C}^\top = \mathbf{C}^\top \mathbf{D}. \tag{17}$$

This last expression implies that the eigenvectors of $\mathbf{G}$ are the sensor weights or the importance of each sensor at a critical point of the signal output energy with respect to the sensors. We extend the approach to the selection of time varying sensors.

5.1.2 Time Variant Sensors

Selecting time varying sensors $\mathbf{C}(t)$ that maximize $\mathcal{M}_2(t)$ at each point in time t can be regarded as solving separate optimization problems at each point in time. Here, our objective function is

$$\mathcal{M}_2(t) = \mathbf{y}(t)^\top \mathbf{y}(t) = \mathbf{x}(0)^\top \Phi(t,t_0)^\top \mathbf{C}(t)^\top \mathbf{C}(t)\Phi(t,t_0)\mathbf{x}(0), \tag{18}$$

which is equivalent to eq. (15) with the removal of the summation over time. Since $\mathbf{C}(t_1)$ and $\mathbf{C}(t_2)$ are independent of one another for all t_1 and t_2, we can maximize the energy at each time $\mathcal{E}(t)$ independently of one another. To do so, we

extend the fixed sensor selection optimization of $\mathcal{M}_2$ to an optimization of $\mathcal{M}_2(t)$, using a similar approach as in [20]. This is formulated as follows:

$$
\begin{aligned}
\frac{\partial(\mathcal{M}_2(t)+\mathcal{L})}{\partial \mathbf{C}(t)^\top} &= \frac{\partial}{\partial \mathbf{C}(t)^\top}\Big(\mathbf{x}(0)^\top\Phi(t,t_0)^\top\mathbf{C}(t)^\top\mathbf{C}(t)\Phi(t,t_0)\mathbf{x}(0) - \mathbf{tr}((\mathbf{C}(t)\mathbf{C}(t)^\top - \mathbf{I})\mathbf{D})\Big)\\
&= \frac{\partial}{\partial \mathbf{C}(t)^\top}\Big(\mathbf{tr}\big(\mathbf{x}(0)^\top\Phi(t,t_0)^\top\mathbf{C}(t)^\top\mathbf{C}(t)\Phi(t,t_0)\mathbf{x}(0)\big) - \mathbf{tr}((\mathbf{C}(t)\mathbf{C}(t)^\top - \mathbf{I})\mathbf{D})\Big)\\
&= \frac{\partial}{\partial \mathbf{C}(t)^\top}\Big(\mathbf{tr}\big(\mathbf{C}(t)\Phi(t,t_0)\mathbf{x}(0)\mathbf{x}(0)^\top\Phi(t,t_0)^\top\mathbf{C}(t)^\top\big) - \mathbf{tr}((\mathbf{C}(t)\mathbf{C}(t)^\top - \mathbf{I})\mathbf{D})\Big)\\
&= \frac{\partial}{\partial \mathbf{C}(t)^\top}\Big(\mathbf{tr}\big(\mathbf{C}(t)\mathbf{G}(t,t_0)\mathbf{C}(t)^\top\big) - \mathbf{tr}((\mathbf{C}(t)\mathbf{C}(t)^\top - \mathbf{I})\mathbf{D})\Big)\\
&= 2\mathbf{G}(t,t_0)\mathbf{C}(t)^\top - 2\mathbf{C}(t)^\top\mathbf{D} = 0.
\end{aligned} \tag{19}
$$

This has a similar interpretation to the time invariant case and says that the eigenvalues of $\mathbf{G}(t,t_0)$ denote the contribution of each state variable to observability at time t. To solve this and select sensors from time $t_0,\ldots,t$, we must form $\mathbf{G}(t,t_0)$ for all t. This requires integrating the system forward from the initial conditions $\mathbf{x}(0)$, which can be performed efficiently using model reduction, and then computing the largest eigenvector of the matrices $\mathbf{G}(t,t_0)$, for which there are fast algorithms. The weights of the eigenvectors of $\mathbf{G}(t,t_0)$ correspond to the contribution to observability provided by each state variable at time t.

5.2 Maximizing the Trace of the Observability Gramian $\mathcal{M}_3$

Here we provide our method to maximize observability measure $\mathcal{M}_3$ with linear programming. We begin by highlighting a few key properties of the observability Gramian for LTV systems, then provide the integer programming formulation to maximize $\mathcal{M}_3$. Finally, we provide a continuous relaxation of the integer programming problem, which can be solved as a linear optimization.

5.2.1 Properties of the Observability Gramian

To formulate the sensor selection problem in terms of integer programming, consider the case where there is a binary variable denoting whether or not each state variable $x_i \in \mathbf{x}$ is observed or measured at time t. Let $\mathbf{c}_{tj}$ be the j-th row of the matrix $\mathbf{C}(t) \in \mathbb{R}^{n\times p(t)}$ such that $\mathbf{c}_{ij}$ is a row vector with j-th entry as 1 and zero otherwise. Then the LTV observability Gramian can be written as

$$
\begin{aligned}
\mathbf{G}_o &= \sum_{i=0}\Phi(i,0)^\top\mathbf{C}(i)^\top\mathbf{C}(i)\Phi(i,0),\\
&= \sum_{i=0}\sum_{j=1}^{p_k}\Phi(i,0)^\top(\mathbf{c}_{ij})^\top\mathbf{c}_{ij}\Phi(i,0).
\end{aligned}
$$

Let $\delta_{tj} \in \{0,1\}$ be a binary variable, which indicates whether x_j variable is measured at time t. Then one can express above relation, as

$$
\mathbf{G}_o(\Delta) = \sum_{i=0}\sum_{j=1}^{n}\delta_{ij}\mathbf{W}_{ij} \text{ where } \mathbf{W}_{ij} = \Phi(i,0)^\top(\mathbf{c}_{ij})^\top\mathbf{c}_{ij}\Phi(i,0),
$$

and, $\Delta = (\delta_{11},\delta_{21},\cdots,\delta_{(T+1)n})^\top \in R^{n(T+1)}$ contains binary variables that indicate which state variables to measure at each time.

5.2.2 Integer Programming Formulation

Given the sensor selection variables Δ, the sensor selection problem can be written as a mixed-integer convex problem,

$$
\max_{\Delta}\mathcal{M}_3(t) = \max_{\Delta}\ trace(\mathbf{G}_o(\Delta)) \text{ subject to } \sum_{j=1}^{n}\delta_{tj} \le p(t), \quad t=0,1,\cdots \quad \text{where} \quad 0 \le \delta_{kj} \in \{0,1\}. \tag{20}
$$

Here, the first constraint restricts number of selected sensors to be no more than $p(t)$ for each time point. Because mixed-integer programs do not scale well for large problems, a convex relaxation to (20) provides a useful solution alternative.

5.2.3 Continuous Relaxation

In the continuous relaxation, the observation of variable j at time t is relaxed to the interval $\delta_{ij} \in [0,1]$ (i.e. $0 \leq \delta_{ij} \leq 1$). This leads to the convex program,

$$\max_{\Delta} \mathcal{M}_3(t) = \max_{\Delta} \; trace(\mathbf{G}_o(\Delta)) \text{ subject to } \quad \sum_{j=1}^{n} \delta_{tj} \leq p(t), \quad t = 0,1,\cdots \quad \text{where} \quad 0 \leq \alpha_{tj} \leq 1. \tag{21}$$

The advantage of the relaxation is that it can be solved in time that is polynomial in the number of variables using efficient techniques such as interior point methods. Furthermore, if the solution to the relaxed problem is such that $\alpha_{ij} \in \{0,1\}$ (within numerical tolerance), then the original mixed-integer problem has been solved. The relaxation serves two roles — an approximate (suboptimal) solution to the mixed-integer problem by rounding α_{ij}, and, in some cases, a fast optimal solution to the mixed-integer problem.

In both the mixed-integer problem (20) and the convex relaxation (21), the desired number of sensors was explicitly constrained to be $p(t)$. Another approach is to allow the number of sensors to be a free variable, and enforce a sparse solution, which can be achieved, for example, by l_1 regularization technique which yields a convex problem,

$$\max_{\Delta} \mathcal{M}_3(t) - c\|\Delta\|_1 \quad 0 \leq \delta_{ij} \leq 1, \tag{22}$$

where the constant $c \geq 0$ is the weighting on the l_1-norm penalty. By varying the weight c, the number of sensors in the solution set will change to balance the sparsity penalty with the observability measure.

6 Supplementary Figures

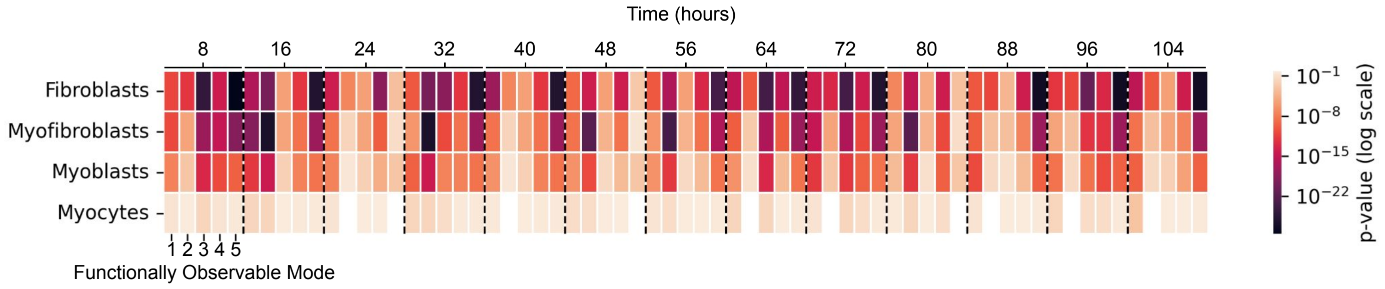

Figure 5: **Functionally Observable Cell Types.** The top 10% of genes that contribute to the first 5 functionally observable modes of the observablility matrix $\mathcal{O}$, which are obtained as the right singular vectors $\mathbf{V}$ from $\mathcal{O} = \mathbf{U}\Sigma\mathbf{V}^{\top}$, are enriched to identify which cell types are observable [65]. In the recreation of Weintraub's reprogramming experiment, Fibroblasts are reprogrammed to myogenic lineages. The functionally observable modes are highly enriched for Fibroblasts, Myofibroblasts and Myoblasts, which are progenitors of Myocytes. Myocytes, which come later in differentiation than Myoblasts, are not functionally observable in this data, which is consistent with the short duration over which the experiment is monitored. The strong enrichment for Fibroblasts and Myogenic lineages indicates that the DSS selected biomarkers make the early stages of reprogramming process functionally observable.

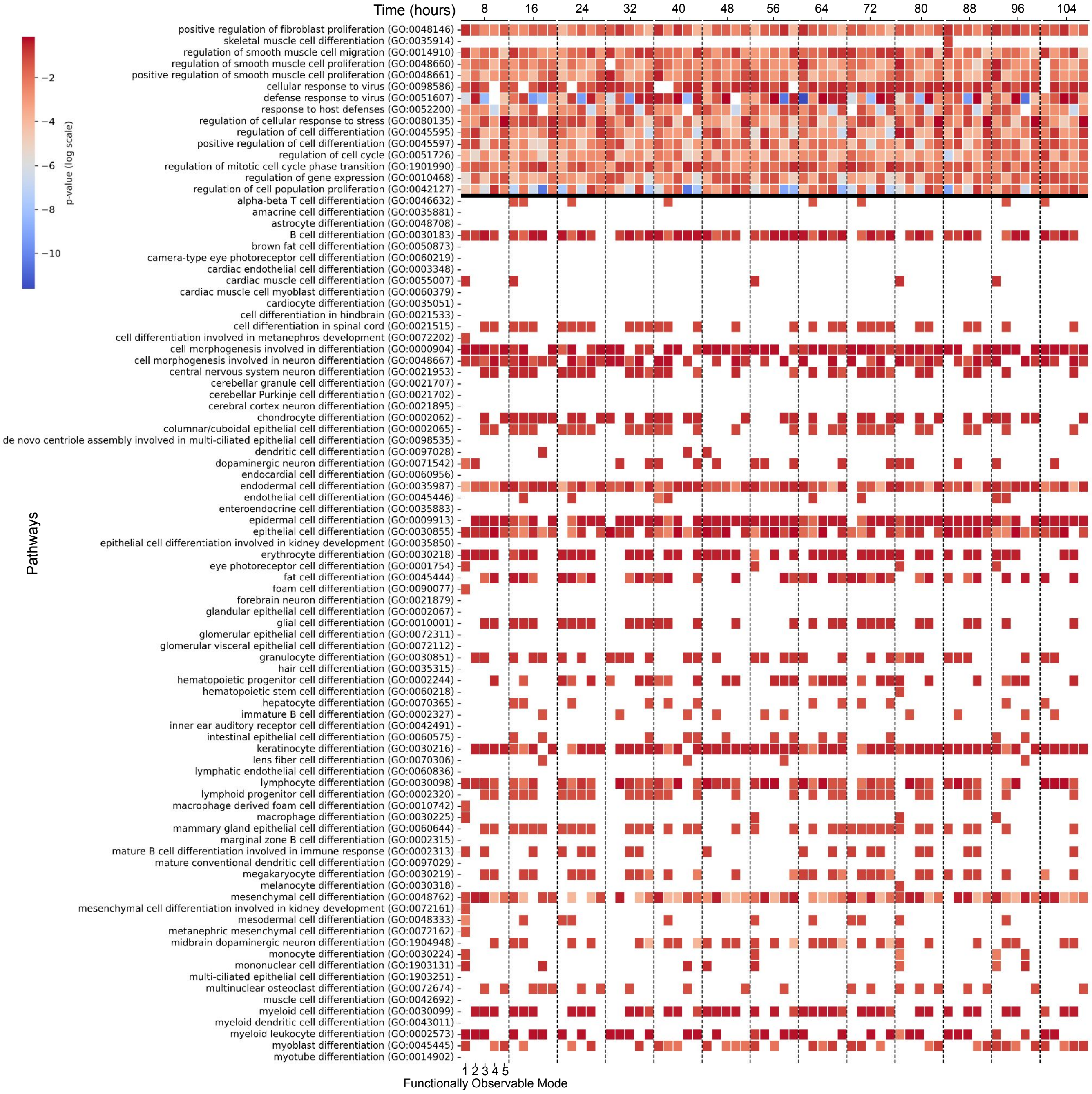

Figure 6: **Functionally Observable Pathways.** The top 10% of genes that contribute to the first 5 functionally observable modes of the system are enriched for the related pathways and processes that are monitored by the selected sensors. There is a strong tendency to monitor processes related to myogenic lineage, such as the regulation of smooth muscle cells, and pathways likely involved in the reprogramming process, such as the cellular response to virus, which is expected due to the Lentivirus used to introduce *MYOD* and initiate reprogramming. To contrast the significance of enrichment for myogenic pathways with differentiation of Fibroblasts to other cell types, the enrichment of pathways involved in differentiation to other cell types is shown below the horizontal black line.

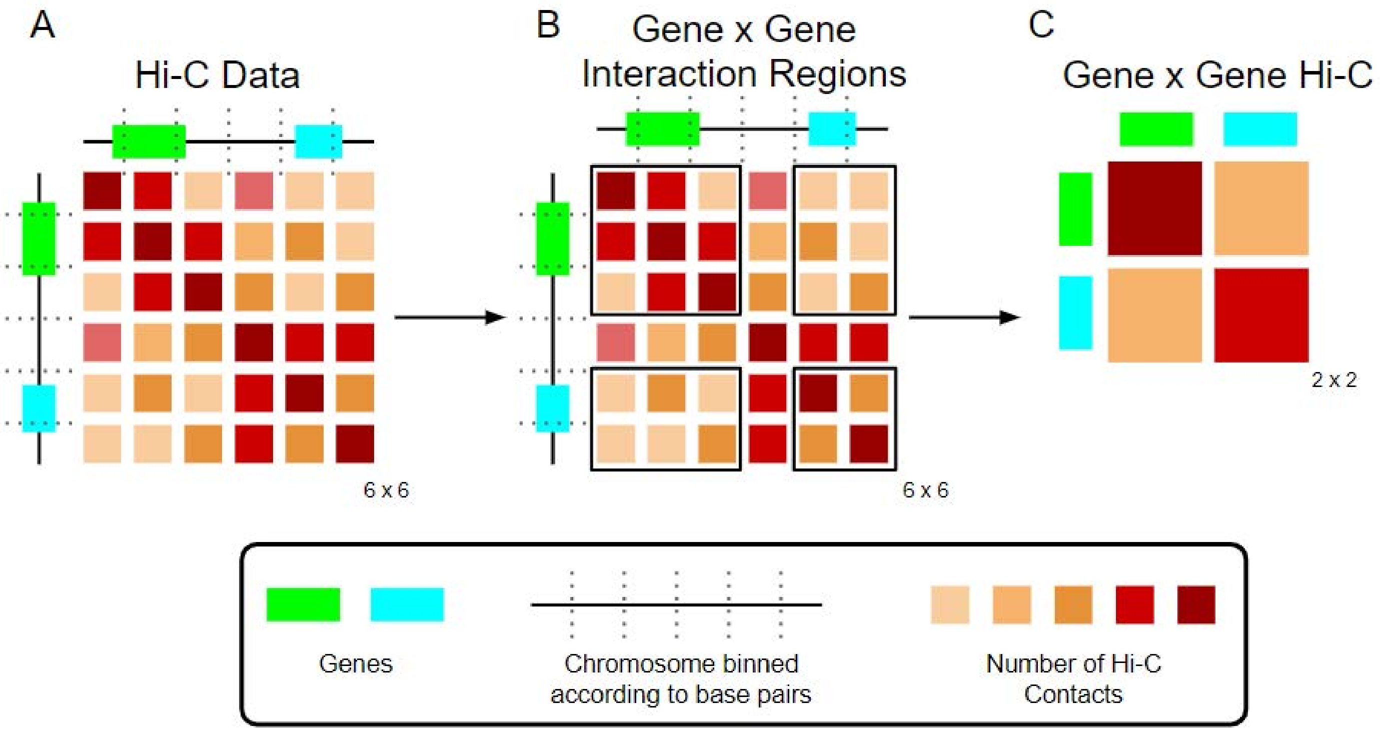

Figure 7: **Constructing Gene by Gene Hi-C Matrices.** Hi-C matrices, processed to any resolution, is constructed so that each Hi-C index or bin represents a fixed length of the genome (left). Based on the gene coding regions, we can identify the segments of Hi-C corresponding to gene-gene interactions (middle). Averaging over these regions, we can construct gene by gene Hi-C matrices where each row/column corresponds to a single gene and a variable length of the linear genome.

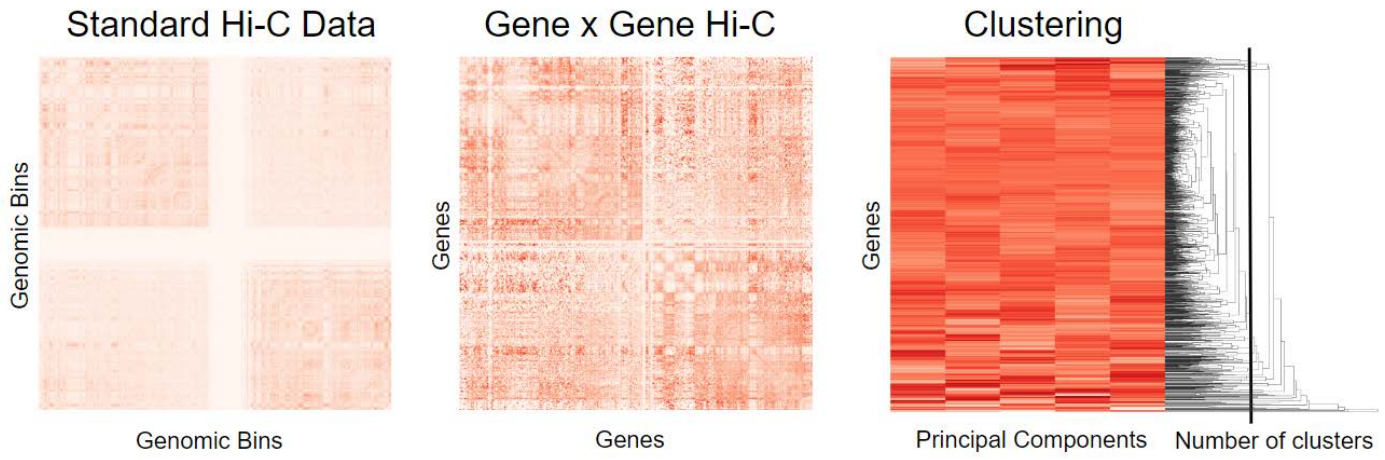

Figure 8: **Gene Clustering from Hi-C.** Standard Hi-C data from chromosome 1 is shown on the left, where genomic bins correspond to a fixed length of chromatin (i.e. 100kb). The gene by gene Hi-C matrix is constructed according the the process outlined above and in fig. 7 (middle). The principal components of the gene by gene Hi-C matrix are used to cluster genes, and the number of clusters is set to maximize the Silhouette score.

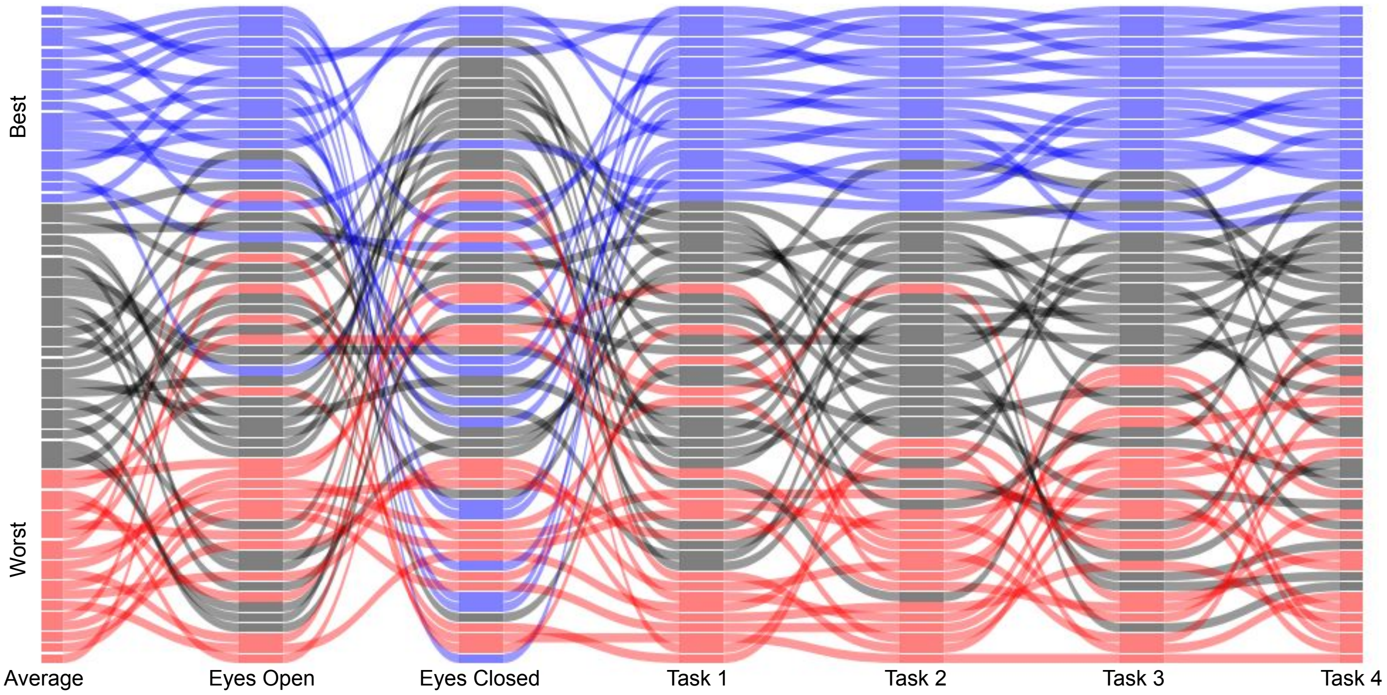

Figure 9: **Observability Contributions from EEG Signals During Six Tasks.** EEG Signals are ranked according to their contribution to observability. This is an extension of Fig 3.F that shows an additional four tasks. Task 1: open and close left or right fist. Task 2: imagine opening and closing left or right fist. Task 3: open and close both fists or both feet. Task 4: imagine opening and closing both fists or both feet.
