# Supplementary material for: Biomarker Selection for Adaptive Systems
Source: ArXiv. 2024 Aug 12:arXiv:2405.09809v3. Preprint. [Version 3] (PMC11142321)
Supplement: Supplementary file 1 [file NIHPP2405.09809V3-supplement-1.pdf]

Supporting Information

Contents

1 Introduction **19**

2 Related Work on Learning Dynamics, Observability, and Sensor Selection **20**

2.1 Learning Dynamics 20
2.2 Observability and Sensor Selection 24

3 Dynamic Sensor Selection **30**

3.1 Oscillator Networks 30
3.2 Sensor Selection Formulations 39
3.3 Output Energy Maximization 39
3.4 Gramian Based Observability 41

4 Structure Guided Sensor Selection **43**

4.1 Small World Networks 43
4.2 Constrained Optimizations 47
4.3 Small Worldness of the Genome 48
4.4 Hi-C Guided Biomarker Identification 52

5 Applications to Data **59**

5.1 Human Fibroblast Proliferation 59
5.2 Cellular Reprogramming 59
5.3 Pesticide Detection in SBW25 60
5.4 Electroencephalogram BCI2000 60
5.5 FUCCI Microscopy 61
5.6 Reference Databases 61

6 Supplementary Figures **63**

1 Introduction

This Supporting Information is organized as follows. Section §2 provides information about how we build LTI and LTV models of dynamics from time series data and previous perspectives on observability. Sections §3 and §4 provide mathematical formulations of Dynamic Sensor Selection and Structure Guided Sensor Selection respectively. Finally, Section §5 outlines particular data sets and results obtained on the data. Supplementary figures are provided in Section §6, and the references in the Supporting Information are distinct from those in the main text.

2 Related Work on Learning Dynamics, Observability, and Sensor Selection

The problem of learning dynamics and selecting sensors or measurements is a wide field of study with a deep history [8, 74, 72, 73]. Here, we clarify some of the particular algorithms we rely on for learning the dynamics of $\mathbf{A}(t)$ and survey classic measures of observability to distinguish our contributions. We consider the time-variant system with linear outputs

$$\begin{aligned} \mathbf{x}(t+1) &= \mathbf{A}(t)\mathbf{x}(t) + \mathbf{B}\mathbf{u}(t) \\ \mathbf{y}(t) &= \mathbf{C}(t)\mathbf{x}(t), \end{aligned} \tag{6}$$

where $\mathbf{x}(t) \in \mathbb{R}^n$ is the state vector, $\mathbf{A}(t) \in \mathbb{R}^{n\times n}$ is the state transition matrix, $\mathbf{C}(t) \in \mathbb{R}^{p(t)\times n}$ is the measurement matrix, and $\mathbf{y}(t) \in \mathbb{R}^{p(t)}$ is the system output. The measurements are lower dimensional than the state of our system (i.e. $p(t) \ll n$).

2.1 Learning Dynamics

We focus on the parameterization of the state transition matrices $\mathbf{A}(t)$ from evenly spaced time series data $\mathbf{X}$, where the data are of the form

$$\mathbf{X} = \begin{bmatrix} | & | & & | & | \\ \mathbf{x}(0) & \mathbf{x}(1) & \dots & \mathbf{x}(T-1) & \mathbf{x}(T) \\ | & | & & | & | \end{bmatrix}.$$

Here, $\mathbf{X} \in \mathbb{R}^{n\times(T+1)}$ is a matrix with n rows, where each row corresponds to a measurement, and T columns, where each column contains all measurements at a particular instant in time. Consistent with many data, we assume the data are collected at even intervals in time (columns), and we will learn a model where each state variable comes from a single measurement (row). We first review Dynamic Mode Decomposition (DMD), then shift focus to the LTV systems under the Data Guided Control (DGC) model.

2.1.1 Dynamic Mode Decomposition (DMD)

Here we outline the basic steps of DMD to provide a concise summary of the models we employ. For a comprehensive review on DMD and its applications in modeling biological and other complex systems, see [60, 103] and references therein. Given time series data $\mathbf{X}$, consider the first and last T samples, denoted by $\mathbf{X}^-$ and $\mathbf{X}^+$ where

$$\mathbf{X} = \begin{bmatrix} | & | & & | & | \\ \mathbf{x}(0) & \mathbf{x}(1) & \dots & \mathbf{x}(T-1) & \mathbf{x}(T) \\ | & | & & | & | \end{bmatrix} \text{ such that } \mathbf{X}^- = \begin{bmatrix} | & | & & | \\ \mathbf{x}(0) & \mathbf{x}(1) & \dots & \mathbf{x}(T-1) \\ | & | & & | \end{bmatrix} \text{ and}$$

$$\mathbf{X}^+ = \begin{bmatrix} | & & | & | \\ \mathbf{x}(1) & \dots & \mathbf{x}(T-1) & \mathbf{x}(T) \\ | & & | & | \end{bmatrix}. \tag{7}$$

The purpose of the DMD algorithm is to learn the best linear model of the dynamics $\mathbf{x}(t+1) = \mathbf{A}\mathbf{x}(t)$ to explain the observed data. Learning the dynamics $\mathbf{A}$ can be formalized by solving the matrix minimization

$$\min_A \|\mathbf{X}^+ - \mathbf{A}\mathbf{X}^-\|_F^2, \tag{8}$$

whose solution is given by $\mathbf{A} = \mathbf{X}^+(\mathbf{X}^-)^\dagger$, where $\dagger$ denotes the pseudo inverse. Consider the Singular Value Decomposition (SVD) $\mathbf{X}^- = \mathbf{U}\Sigma\mathbf{V}^\top$, where, $\mathbf{U} \in \mathbb{R}^{n\times r}$, $\mathbf{\Sigma} \in \mathbb{R}^{r\times r}$ and $\mathbf{V} \in \mathbb{R}^{(T-1)\times r}$ and $r \ll n$. The least squares problem eq. (8) has the solution

$$\mathbf{A} = \mathbf{X}^+(\mathbf{X}^-)^\dagger = \mathbf{X}^+\mathbf{V}\mathbf{\Sigma}^{-1}\mathbf{U}^\top,$$

which is the best linear model to explain the data $\mathbf{X}$.

Often, when we have multiple replicates of an experiment, rather than the data $\mathbf{X}$ being a $n \times T$ matrix, $\mathbf{X}$ will be a $n \times T \times r$ tensor, where r denotes the number of replicates or separate instances of time series data. When there are multiple replicates, as in the case of all time series datasets we consider, we form $\mathbf{X}^+$ and $\mathbf{X}^-$ similarly by removing the first and last time points to generate $n \times (T-1) \times r$ tensors. Alternatively, the data can be averaged over multiple replicates to obtain a $n \times T$ matrix and be treated similarly as if only one replicate had been obtained.

2.1.2 Model Reduction

DMD is classically applied to physical systems, which are often relatively low dimensional when compared with gene expression and biological systems. The high dimensional number of variables in omics data lends itself to state space models of higher dimensions than most applications of DMD. For instance, the human genome contains on the order of 20 thousand genes, each of which can be modeled as a state variable in $\mathbf{x}$; however, such a model omits other important biological states or features such as isoform or protein expression. These large state space require model reduction to be computationally tractable, whereby the number of free parameters is reduced. Such an approach is consistent with many biological processes, where understanding the dynamics of only a few variables is sufficient to understand the dynamics of the system.

To study the dynamics of our high dimensional data $\mathbf{X}$, we can change the coordinates to a lower dimensional space where it is easier to integrate, compute, and

analyze our model. Principal component (PC) based model reduction is standard to DMD. From computing the SVD of $\mathbf{X}^-$ in the DMD algorithm, the left singular vectors or PCs $\mathbf{U}$ map gene expression to principal component space. To make such a change of coordinates, we apply the transformation $\tilde{\mathbf{x}}(t) = \mathbf{U}^\top \mathbf{x}(t)$. Then the reduced state vector $\tilde{\mathbf{x}}$ will have r components where r is the rank of the data. Applying this transformation, we can produce a model reduced state transition matrix $\tilde{\mathbf{A}}$, where $\tilde{\mathbf{A}} = \mathbf{U}^\top \mathbf{A}\mathbf{U}$. To see this reduction, consider

$$\tilde{\mathbf{x}}(t+1) = \mathbf{U}^\top \mathbf{x}(t+1) = \mathbf{U}^\top \mathbf{A}\mathbf{x}(t) = \mathbf{U}^\top \mathbf{A}\mathbf{U}\tilde{\mathbf{x}}(t) = \tilde{\mathbf{A}}\tilde{\mathbf{x}}(t).$$

From this, $\tilde{\mathbf{A}}$ can be derived directly from the data $\mathbf{X}$ as

$$\tilde{\mathbf{A}} = \mathbf{U}^\top \mathbf{A}\mathbf{U} = \mathbf{U}^\top \mathbf{X}^+ \mathbf{V}\mathbf{\Sigma}^{-1},$$

which is $r \times r$ matrix.

Returning to the observability of eq. (6), we can cast the system output measurements $\mathbf{y}$ in terms of the model reduced system as

$$\begin{aligned} \tilde{\mathbf{x}}(t+1) &= \tilde{\mathbf{A}}\tilde{\mathbf{x}}(t), \\ \mathbf{y}(t) &= \mathbf{C}\mathbf{U}\tilde{\mathbf{x}}(t), \end{aligned}$$

where $\tilde{\mathbf{x}}(0) = \mathbf{U}^\top \mathbf{x}(0)$. Thus, sensor selection problem can be cast in terms of reduced system.

2.1.3 Switching Systems

Transitioning from LTI systems learned with DMD to LTV dynamics, consider a switching system, where there are two models of dynamics $\mathbf{A}_1$ and $\mathbf{A}_2$ that describe the flow of the system during two distinct phases in time. The dynamics of this model can be described as

$$\begin{aligned} \mathbf{x}(t+1) &= \mathbf{A}_1\mathbf{x}(t), \qquad 0 \le t \le T_s \\ \mathbf{x}(t+1) &= \mathbf{A}_2\mathbf{x}(t), \qquad t > T_s, \end{aligned}$$

where T_s denotes the time at which the dynamics switch from the first phase with $\mathbf{A}_1$ dynamics to the second phase with $\mathbf{A}_2$ dynamics. Dynamics such as these are well studied in social and communication systems and also characteristic of many biological dynamics such as bistable systems in cell regulation [124, 2].

Given data $\mathbf{X}_1$ is generated before T_s and $\mathbf{X}_2$ after T_s, we can employ the DMD algorithm to model the dynamics $\mathbf{A}_1$ and $\mathbf{A}_2$ for each respective time period. Let $\mathbf{X}_i = \mathbf{U}_i\mathbf{\Sigma}_i\mathbf{V}_i^\top$. Model reduction can then be applied to each time period, so that the dynamics of the system are expressed

$$\begin{aligned} \tilde{\mathbf{x}}(t+1) &= \tilde{\mathbf{A}}_1\tilde{\mathbf{x}}(t), \qquad 0 \le t \le T_s \\ \tilde{\mathbf{x}}(t+1) &= \tilde{\mathbf{A}}_2\tilde{\mathbf{x}}(t), \qquad t > T_s \end{aligned}$$

The full state space and reduced model are related to one another from the relation $\mathbf{x}(t) = \mathbf{U}_i^\top \mathbf{x}(t)$, where $i = 1$ or 2, depending on the period of the system. Given an initial condition $\mathbf{x}_0$ the system evolves as

$$\begin{aligned}
\tilde{\mathbf{x}}(1) &= \tilde{\mathbf{A}}_1 \mathbf{U}_1^\top \mathbf{x}(0), && \text{(model reduction)} \\
\tilde{\mathbf{x}}(2) &= \tilde{\mathbf{A}}_1 \tilde{\mathbf{x}}(1), && \text{(step forward)} \\
\vdots &= \vdots \\
\tilde{\mathbf{x}}(T_s) &= \tilde{\mathbf{A}}_1 \tilde{\mathbf{x}}(T_s - 1), \\
\tilde{\mathbf{x}}(T_s + 1) &= \tilde{\mathbf{A}}_2 \mathbf{U}_2^\top \mathbf{U}_1 \tilde{\mathbf{x}}(T_s), && \text{(switch dynamics)} \\
\tilde{\mathbf{x}}(T_s + 2) &= \tilde{\mathbf{A}}_2 \tilde{\mathbf{x}}(T_s + 1), && \text{(step forward)} \\
\vdots &= \vdots \\
\tilde{\mathbf{x}}(T) &= \tilde{\mathbf{A}}_2 \tilde{\mathbf{x}}(T - 1).
\end{aligned}$$

Thus, at any time point t, the reduced, one step, state transition matrix for a switching system can be written:

$$\tilde{\mathbf{A}}_t = \begin{cases} \tilde{\mathbf{A}}_1 \mathbf{U}_1^\top, & \text{if } t = 0 \\ \tilde{\mathbf{A}}_1, & \text{if } 0 < t \leq T_s \\ \tilde{\mathbf{A}}_2 \mathbf{U}_2^\top \mathbf{U}_1, & \text{if } t = T_s + 1 \\ \tilde{\mathbf{A}}_2, & \text{if } t > T_s + 1 \end{cases}.$$

2.1.4 State Transition Matrices Φ

Given time points time points t_1 and t_2 where $t_1 \leq t_2$, we are interested in defining a single transition matrix between these times. For a LTI system, the transition matrix between t_1 and t_2 is found as $\mathbf{A}^{t_2 - t_1}$ or via a similar matrix exponentiation for the continuous case. For the LTV system eq. (6), we can define a matrix $\Phi(t_2, t_1)$ that maps directly from time t_1 to t_2 as

$$\Phi(t_2, t_1) = \mathbf{A}(t_2 - 1)\mathbf{A}(t_2 - 2) \cdots \mathbf{A}(t_1).$$

Then, given the initial conditions $\mathbf{x}_{t_0}$ and the control signals $\mathbf{u}_{t_0}, \mathbf{u}_{t_0+1}, \cdots, \mathbf{u}_{t-1}$ input to eq. (6), the state $\mathbf{x}(t)$ can be written as

$$\mathbf{x}(t) = \Phi(t, t_0)\mathbf{x}_{t_0} + \sum_{i=t_0}^{t-1} \Phi(t, i+1)\mathbf{B}(i)\mathbf{u}(i).$$

The transition matrix Φ has following properties:

$$\begin{aligned}
\Phi(k_2, k_0) &= \Phi(k_2, k_1)\Phi(k_1, k_0) \qquad k_0 \leq k_1 \leq k_2, \\
\Phi(k, k) &= \mathbf{I}.
\end{aligned}$$

These properties hold for the reduced transition matrix $\tilde{\Phi}$, which is similarly defined as $\tilde{\Phi}(t_1, t_2) = \tilde{\mathbf{A}}(t_2 - 1)\tilde{\mathbf{A}}(t_2 - 2)\cdots\tilde{\mathbf{A}}(t_1)$.

2.1.5 Data Guided Control (DGC) Model

The DGC model for approximating time variant linear systems was proposed by [100] to model the dynamics throughout cell reprogramming. This approach to learning dynamics considered the discrete-time LTV system with control:

$$\mathbf{x}(t+1) = \mathbf{A}(t)\mathbf{x}(t) + \mathbf{B}\mathbf{u}(t), \tag{9}$$

where $\mathbf{B}_t$ is the control configuration matrix and $\mathbf{u}$ is the input or control signal.

The fundamental assumption of the model is that gene expression of a population does not change considerably over time. Hence, the state transition matrix $\mathbf{A}$ should be similar to the identity $\mathbf{I}$. From this, the authors of [100] define $\mathbf{A}(t)$ as a rank one perturbation from the identity:

$$\mathbf{A}(t) = \mathbf{I} + \frac{(\mathbf{x}(t+1) - \mathbf{x}(t))\mathbf{x}(t)^\top}{\mathbf{x}(t)^\top\mathbf{x}(t)}. \tag{10}$$

The authors then posed the challenge of selecting $\mathbf{u}(t)$ for cell reprogramming as an optimal control problem. Based on the success of this model for controlling cell dynamics, we chose to study its observability.

2.2 Observability and Sensor Selection

The problem of observing dynamical systems is a broad area with deep roots in systems theory [54, 35]. Mathematically, the ability to uniquely determine $\mathbf{x}(0)$ is sufficient to call a system observable, since the knowledge of $\mathbf{A}$, $\mathbf{x}(0)$ and possibly a known control signal is sufficient to determine the state $\mathbf{x}(t)$ at any future point in time. Here we survey several classic tests for observability of linear systems, challenges associated with nonlinear observability, and how we can use observed outputs to estimate the full state of a system.

2.2.1 Tests for Observability of Linear Systems

Classic tests for observability seek to provide binary criteria regarding if eq. (27) is observable. From the binary perspective, a system is mathematically observable if the full system initial condition $\mathbf{x}(0)$ can be uniquely determined. In many instances, however, it is beneficial to consider more refined notions of observability, such as local observability, which is the ability to determine $\mathbf{x}(0)$ within a subset of possible states, or targeted observability, which is the ability to determine a subset of relevant state variables in $\mathbf{x}(0)$ [106, 33, 83, 82, 81, 9, 50, 6, 104, 91]. Here, we survey the Kalman Rank Condition, Popov-Belevitch-Hautus Test, and Structural Observability, three classical tests of observability.

Kalman Rank Condition. The Kalman Rank condition for observability, likely the most famous criteria, guarantees a system is observable when the initial state of the system $\mathbf{x}_0$ can be uniquely deteremined from the measurements $\mathbf{y}(0), \ldots, \mathbf{y}(n-1)$. As a test for this, the rank of the so called observability matrix $\mathcal{O}$ is compared with the dimension of the state $\mathbf{x}$ so that when

$$rank(\mathcal{O}) = dim(\mathbf{x}) \text{ where } \mathcal{O} = \begin{bmatrix} \mathbf{C} \\ \mathbf{CA} \\ \mathbf{CA}^2 \\ \vdots \\ \mathbf{CA}^{n-1} \end{bmatrix} \text{ such that } \begin{bmatrix} \mathbf{y}(0) \\ \mathbf{y}(1) \\ \mathbf{y}(2) \\ \vdots \\ \mathbf{y}(n-1) \end{bmatrix} = \begin{bmatrix} \mathbf{C} \\ \mathbf{CA} \\ \mathbf{CA}^2 \\ \vdots \\ \mathbf{CA}^{n-1} \end{bmatrix} \mathbf{x}(0) \quad (11)$$

has a unique solution for $\mathbf{x}(0)$, the system is observable. This approach is the foundation upon which subsequent tests and our estimation procedure are developed.

PBH Test. Building upon the Kalman Condition, the Popov-Belevitch-Hautus (PBH) test, also known as the Hautus Lemma, guarantees observability where

$$rank(\mathcal{P}) = \dim(\mathbf{x}) \text{ where } \mathcal{P} = \begin{bmatrix} \mathbf{A} - \lambda \mathbf{I} \\ \mathbf{C} \end{bmatrix},$$

for all eigenvalues λ of $\mathbf{A}$ [94]. The rank deficiency of the PBH test can be seen as the existance of an eigenvector in the null space of the observability matrix $\mathcal{O}$. By verifying $rank(\mathcal{P}) = n$ for all λ, the PBH test provides an equivalent check that $rank(\mathcal{O}) = n$ or that $\mathbf{x}(0)$ can be deteremined from the first n measurements $\mathbf{y}(0), \ldots, \mathbf{y}(n-1)$.

Although the Kalman Rank Condition and PBH Test offer convenient and consistent methods for assessing the observability of a LTI system, based on the matrices defining dynamics and observables, they are seldom utilized in practice due to three main challenges:

(C1) Identifiability: If we fail to identify $\mathbf{A}$ to infinite precision, which will certainly be the case in any experimental system such as the ones studied here, then both the Kalman Rank Condition and PBH Test will almost certainly be observable [63].

(C2) Cost: Explicitly constructing and determining the rank of $\mathcal{O}$ or $\mathcal{P}$ for all λ is relatively expensive, particularly for high dimensional systems. For instance, many numerical schemes compute the SVD to determine the matrix rank, which is approximately a 3rd order polynomial operation, once the matrix is constructed.

(C3) Condition: Both the PBH and Kalman conditions provide a binary condition for observability where either YES a system is observable or NO it is not. In practice, however, many systems which are experimentally studied are unobservable, yet this does not deter us from gaining new insights. Rather, a graded degree of observability or targeted observability, where we are interested in only a few hidden states, is sufficient to understand internal dynamics from limited outputs.

To address C1-3, various alternatives have been proposed. For instance, Lin's structural controllability, in the next section, addressed both C1 and C2. Continuing this work, the measures of observability and formulations proposed in §3 address C2 and C3 by allowing fast algorithms to provide scalar measures of observability, and the structural constraints of §4 are designed to address C1.

Lin's Structural Observability. To address the concern of system identifiability and, more recently, the cost involved in assessing observability for large or high dimensional systems, the theory of structural controllability was developed for LTI systems, and structural observability is formulated similarly [68, 70, 71]. To address (C1), Lin identified graph structures, based on the sparsity structures of $\mathbf{A}$ and $\mathbf{C}$, that allow LTI dynamics to be observable based on the PBT test [68]. To address (C2), Liu, Slotine, and Barabási developed the Minimum Inputs theorem from which a fast, graph based maximum matching algorithm can be applied to efficiently select sensor nodes.

Structural observability and controllability have been employed to study a variety of input/output systems across domains [105, 78, 84, 15, 7, 115]. Yet there remain limitations to this approach as well [20] For our work in particular, it leaves challenge C3 unanswered, as structural observability provides a binary YES/NO condition.

2.2.2 Measures of Observability

The system output or observability energy provides a direct method to measure observability and address (C3). Output energy quantifies the amount of energy, defined as a norm, of the output measurements transmitted from a system $\mathbf{y}(0), \mathbf{y}(1), \ldots$ with the equation

$$\mathcal{E} = \sum_{t=0} \mathbf{y}(t)^\top \mathbf{y}(t). \tag{12}$$

When the output energy is small or zero, we lack useful information about the system's state. Therefore, one way to frame the sensor selection problem is as a maximization of the output energy.

The observability Gramian is a generalization of output energy from which many measures of observability have been proposed. Based on the relation that $\mathbf{y}(t) = \mathbf{C}\mathbf{A}^t\mathbf{x}(0)$, the Gramian generalizes eq. (12) as

$$\mathbb{G}_o = \sum_{t=0}^{\infty} (\mathbf{A}^t)^\top \mathbf{C}^\top \mathbf{C}\mathbf{A}^t = \mathcal{O}^\top \mathcal{O}. \tag{13}$$

This definition of the observability Gramian can also be derived from the Lyaponov equation $\mathbf{A}^\top \mathbb{G} + \mathbb{G}\mathbf{A} = -\mathbf{C}^\top \mathbf{C}$. Due to the infinite summation, $\mathbf{A}$ must be stable with all eigenvalues bound inside the unit circle to prevent $\mathbb{G}$ from diverging.

The eigenvalues of the $\mathbb{G}_o$ represent the relative observability of each system mode:

- The minimum eigenvalue $\lambda_{min}(\mathbb{G}_o)$ is a measure of the output energy for the least observable mode, and $\lambda_{min}^{-1}(\mathbb{G}_o)$ characterizes the maximum estimation uncertainty.

- The maximum eigenvalue $\lambda_{max}(\mathbb{G}_o)$ is the measure of the output energy for the most observable mode, and $\lambda_{max}^{-1}(\mathbb{G}_o)$ characterizes the minimum estimation uncertainty.
- The eigenvector corresponding to the maximum eigenvalue, $\lambda_{max}(\mathbb{G}_o)$, is the direction with the largest gain (and thus most observable mode), therefore a small perturbation in that direction yields an output energy equivalent to that of a larger perturbation in the direction of the eigenvector corresponding to the minimum eigenvalue, $\lambda_{min}(\mathbb{G}_o)$ (direction of the least observable mode). Because the condition number $\kappa(\mathbb{G}_o) = \lambda_{max}(\mathbb{G}_o)/\lambda_{min}(\mathbb{G}_o)$ measures the ratio of maximum eigenvalue to minimum eigenvalue, an observability gramian $\mathbb{G}_o$ with a large condition number indicates that the output energy is dominated by some modes, while others are difficult to observe. Furthermore, $\kappa(\mathbb{G}_o^{-1})$ captures the shape of estimation uncertainty ellipsoid.
- The $\log det(\mathbb{G}_o)$ and equivalently $\log det(\mathbb{G}_o^{-1})$ measures the log of the volume of estimation uncertainty ellipsoid.
- The trace of the Gramian, i.e., $\mathbf{tr}[\mathbb{G}_o]$ captures the average output energy, and equivalently $tr[\mathbb{G}_o^{-1}]$ measure the average estimation uncertainty.

Based on these, the following measures of observability have been proposed,

$$\begin{gathered} J_1(\mathbb{G}_o) = \mathbf{tr}[\mathbb{G}_o^{-1}], \quad J_2(\mathbb{G}_o) = \log det(\mathbb{G}_o^{-1}), \quad J_3(\mathbb{G}_o) = -\lambda_{min}(\mathbb{G}_o), \\ J_4(\mathbb{G}_o) = rank(\mathbb{G}_o), \text{ and } J_5(\mathbb{G}_o) = \mathbf{tr}[\mathbb{G}_o], \end{gathered} \tag{14}$$

which needs to be minimized to minimize the uncertainty in state estimate. Note that J_1 and J_2 are convex functions. Also minimizing $\lambda_{min}^{-1}(\mathbb{G}_o)$ is equivalent to maximizing $\lambda_{max}(\mathbb{G}_o)$ which is a concave function. Hence, J_3 is also a convex function.

All the measures J_i for $i = 1, 2, 3$ are defined only when $\mathbb{G}_o$ is full rank. To handle such unobservable cases one could use metrics such as $\mathbf{tr}[\mathbb{G}_o^{\dagger}]$, and corresponds to the average energy required to move the system around the observable subspace, or the log product of nonzero eigenvalues which relates to the "volume" of the subspace reachable with one unit of input energy.

2.2.3 Least Squares Estimation

The mathematical notion of observability and our ability to estimate hidden states of a system from limited output are intimately related. The relationship between these concepts begins with the Kalman Rank Condition, which guarantees mathematical observability for LTI systems when there is a unique solution for $\mathbf{x}(0)$ to the system

$$\begin{bmatrix} \mathbf{y}(0) \\ \mathbf{y}(1) \\ \mathbf{y}(2) \\ \vdots \\ \mathbf{y}(n-1) \end{bmatrix} = \begin{bmatrix} \mathbf{C} \\ \mathbf{CA} \\ \mathbf{CA}^2 \\ \vdots \\ \mathbf{CA}^{n-1} \end{bmatrix} \mathbf{x}(0). \tag{15}$$

This problem is solved for $\mathbf{x}(0)$ directly as $\mathbf{x}(0) = \mathcal{O}^{\dagger}\mathbf{Y}$, where $\mathbf{Y}$ is the left matrix in eq. (15) and $\mathcal{O}$ is the observability matrix as defined in section 2.2.1. This approach is readily adapted for LTV with control. Given a LTV system eq. (6), with time dependent dynamics $\mathbf{A}(t)$, measurements $\mathbf{C}(t)$, input matrices $\mathbf{B}(t)$, and control signals $\mathbf{u}(t)$, the output measurements of the system evolve over time as

$$\begin{aligned}
\mathbf{y}(0) &= \mathbf{C}(0)\mathbf{x}(0), \\
\mathbf{y}(1) &= \mathbf{C}(1)\mathbf{x}(1) = \mathbf{C}(1)\Phi(1,0)\mathbf{x}(0) + \mathbf{C}(1)\mathbf{B}(0)\mathbf{u}(0), \\
\vdots &= \vdots \\
\mathbf{y}(t) &= \mathbf{C}(T)\mathbf{x}(t) = \mathbf{C}(T)\Phi(T,0)\mathbf{x}(0) + \mathbf{C}(T)\sum_{i=0}^{T-1}\Phi(T,i+1)\mathbf{B}(i)\mathbf{u}(i).
\end{aligned}$$

Thus, one can express the system as,

$$\mathbf{Y} = \mathcal{O}\mathbf{x}(0) + \mathbf{U} \qquad \text{with the solution} \qquad \mathbf{x}(0) = \mathcal{O}^{\dagger}(\mathbf{Y} - \mathbf{U}) \tag{16}$$

where

$$\mathbf{Y} = \begin{bmatrix} \mathbf{y}(0) \\ \mathbf{y}(1) \\ \vdots \\ \mathbf{y}(T) \end{bmatrix}, \quad \mathcal{O} = \begin{bmatrix} \mathbf{C}(0) \\ \mathbf{C}(1)\Phi(1,0) \\ \vdots \\ \mathbf{C}(T)\Phi(T,0) \end{bmatrix}, \quad \text{and} \quad \mathbf{U} = \begin{bmatrix} \mathbf{0} \\ \mathbf{C}(1)\mathbf{B}(0)\mathbf{u}(0) \\ \vdots \\ \mathbf{C}(T)\sum_{i=0}^{T-1}\Phi(T,i+1)\mathbf{B}(i)\mathbf{u}(i) \end{bmatrix}. \tag{17}$$

There is a unique solution for $\mathbf{x}(0)$ in the above two equations when $\mathcal{O}^{\dagger}$ is full rank, motivating the Kalman Rank condition to check that $rank(\mathcal{O}) = dim(\mathbf{x})$.

Assuming that the measurements $\mathbf{y}$ are corrupted by independent and identically distributed (i.i.d.) zero-mean Gaussian noise with normal distribution $\mathcal{N}(0, \sigma\mathbf{I})$, using a weighted least squares formulation, the minimum variance estimate for $\hat{\mathbf{x}}^*(0)$ is given by,

$$\hat{\mathbf{x}}^*(0) = (\mathcal{O}^{\top}\mathcal{O})^{-1}\mathcal{O}^{\top}(\mathbf{Y} - \mathbf{U}). \tag{18}$$

The estimate of $\hat{\mathbf{x}}_0^*$ has error covariance $\mathbf{P}_{\mathbf{x}_0}$

$$\mathbf{P}_{\mathbf{x}(0)} = \mathbb{E}\big[(\hat{\mathbf{x}}^*(0) - \mathbf{x}(0))(\hat{\mathbf{x}}^*(0) - \mathbf{x}(0))^{\top}\big] = \sigma(\mathcal{O}^{\top}\mathcal{O})^{-1} = \sigma(\mathbb{G}_o)^{-1}.$$

The least squares estimator is known to be efficient (i.e., the Cramer-Rao lower bound is achieved), therefore the Fisher Information Matrix (FIM) $\mathbf{F} = \sigma^{-1}\mathcal{O}^{\top}\mathcal{O}$ is exactly the inverse of the estimation covariance [107, 21].

To see the relation between the LS estimation proceedure and the Gramian, note that the observability Gramian can be expressed in terms of $\mathcal{O}$ based on the rela-

tionship

$$\begin{aligned}\mathcal{O}^\top\mathcal{O} &= \begin{bmatrix}\mathbf{C}(0)^\top & \Phi(1,0)^\top\mathbf{C}(1)^\top & \cdots & \Phi(T,0)^\top\mathbf{C}(T)^\top\end{bmatrix}\begin{bmatrix}\mathbf{C}_0 \\ \mathbf{C}_1\Phi(1,0) \\ \vdots \\ \mathbf{C}_T\Phi(T,0)\end{bmatrix}, \\ &= \sum_{i=0}^{T}\Phi(i,0)^\top\mathbf{C}(i)^\top\mathbf{C}(i)\Phi(i,0) = \mathbb{G}_o(0). \end{aligned} \tag{19}$$

By relation (19), the observability gramian $\mathbb{G}_o$ is proportional to the FIM and inversely proportional to the estimate covariance. Consequently, the eigenvalues of the observability Gramian directly control the Fisher information and inversely control the estimation covariance, and can be used to define sensor selection measures/metrics. Moreover, when $\mathbb{G}_o$ is full rank, the least squares estimation of $\hat{\mathbf{x}}(0)^*$ in eq. (18) is well determined.

Despite this relationship between observability and estimating $\mathbf{x}(0)$, to date, the marriage of these concepts for biological systems has been prevented due to practical considerations.

- Noise: Experimental observations or measurements in a real system are corrupted with noise.
- Scalability: the least squares approach requires a significant number of observations to achieve the theoretical guarantee of observability.

In the experimental setting, our measurements $\mathbf{y}(0), \mathbf{y}(1), \ldots$ will always be corrupted with noise, and for high dimensional systems where $\mathbf{x} \in \mathbb{R}^n$ and n is large, such as genomics data ($n \gg 20,000$), it is cost prohibitive and experimentally infeasible to obtain n measurements. Fortunately, through careful sensor selection truncated observability matrices $\mathcal{O}$, where the number of rows are truncated to only include time points for which we have data, can be designed so that least squares estimation is still well conditioned. We aim to address these considerations in addition to C1-3 in the following sections regarding Dynamic and Structure Guided Sensor Selection.

3 Dynamic Sensor Selection

In this section, we return to eq. (6) and address the challenge of selecting the best measurement matrices $\mathbf{C}(t)$ when given known dynamics and state transition matrices $\mathbf{A}(t)$. We first review some basic objects and control perspectives on observability for LTI systems and then present two formulations for sensor selection for time variant systems.

3.1 Oscillator Networks

Oscillator networks are characterized by internal dynamics of each oscillator, as well as interactions among them. This is described by the equation:

$$\frac{d\mathbf{x}(t)}{dt} = \mathbf{F}(\mathbf{x}(t), \mu) - \mathbf{L}(\mathbf{x}(t)), \tag{20}$$

as seen in eq. (2). Here, $\mathbf{x}$ is a vector representing the locations or values of each oscillator, F is the dynamics of individual oscillators with internal parameters μ, and L is the diffusion operator specific to the network structure.

The dynamics of $\mathbf{F}(\mathbf{x}(t))$ can be any oscillator, such as the Van der Pol or Andorov-Hopf oscillators, where each oscillator may possess its unique parameters. Additionally, the coupling between oscillators in $\mathbf{L}(\mathbf{x}(t))$ can occur across networks with diverse directionalities and weights. This framework via eq. (20) encompasses several well-known oscillators, including: (1) the Kuromoto oscillator, in which individual oscillators positioned along the unit circle interact with sinusoidal functions [59]; (2) Turing's Equation, which generates oscillations by coupling of two stable systems, and Smale's related work [113, 76].

Continuing the work of Kuromoto, Turing, and Smale, systems of this form have garnered recent interest in the study of higher order structures. For instance, network motifs couple groups of oscillators to form complex and emergent behavior that have been studying in network science [1], systems biology [5, 4], communication systems [64], among other systems. In network biology, genetic circuits such the Repressilator [26], Goodwin Oscillator [40, 39], Toggle Switch [32] can be mathematically expressed in the form of eq. (20).

3.1.1 Repressilator

The canonical Repressilator shown in fig. 3 is one such instance of a network of coupled oscillators, each of which represents the expression from DNA to mRNA and finally protein. In *Escherichia coli.*, the expression from mRNA to protein of lacI, tetR, and cl can be seen as three osicillators, where the regulation the mRNA and protein concentration for a single gene is both regulated internally ($\mathbf{F}(\mathbf{x}(t))$) and repressed by coupled proteins ($\mathbf{L}(\mathbf{x}(t))$). Borrowed from [26], the continuous deterministic Repressilator has dynamics

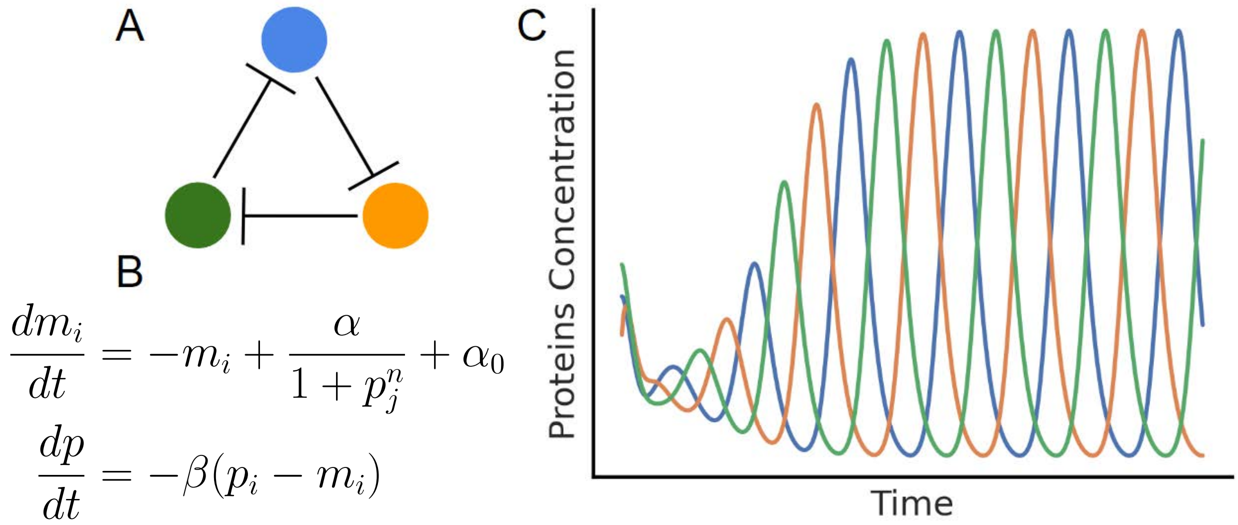

Figure 3: **Repressilator. (A)** The Repressilator contains three nodes, each of which represents the expression of a gene. These nodes are coupled in an inhibitory chain where as the state or concentration of one gene increases, it will inhibit expression of the next gene in the chain. **(B)** The dynamics of a single node are expressed with these differential equations where m_i and p_i denote the concentrations of mRNA and protein associated with gene i respectively. Consistent with the biology, production of protein is positively regulated by the mRNA concentration and both mRNA and protein negatively regulate themselves. Additionally, the production of mRNA of gene i decreases with the concentration of protein for gene j to encode the inhibitory relationship. The full system of equations for all three genes together is in eq. (21)**(C)** We simulate several oscillations of the Repressilator from random initial conditions with $\alpha = 20, \alpha_0 = 0, \beta = 2$ and $n = 2.1$.

$$
\frac{dm_{lacI}}{dt} = \overbrace{-m_{lacI} + \alpha_0}^{\mathbf{F}(\mathbf{x})} \overbrace{+\frac{\alpha}{1+p_{cl}^n}}^{-\mathbf{L}(\mathbf{x})}, \quad \frac{dp_{lacI}}{dt} = \overbrace{-\beta p_{lacI}}^{\mathbf{F}(\mathbf{x})} \overbrace{+\beta m_{lacI}}^{-\mathbf{L}(\mathbf{x})},
$$

$$
\frac{dm_{tetR}}{dt} = -m_{tetR} + \alpha_0 + \frac{\alpha}{1+p_{lacI}^n}, \quad \frac{dp_{tetR}}{dt} = -\beta p_{tetR} + \beta m_{tetR},
$$

$$
\frac{dm_{cl}}{dt} = -m_{cl} + \alpha_0 + \frac{\alpha}{1+p_{tetR}^n}, \quad \frac{dp_{cl}}{dt} = -\beta p_{cl} + \beta m_{cl}. \tag{21}
$$

Here, there are have six chemical species describing the mRNA (m) and protein (p) concentrations of the lacI, tetR, and cl genes; α, β, and n, are parameters; and there is a pair of equations to describe the dynamics of mRNA and protein concentrations separately. Each form of the equations can be expressed in terms of eq. (20), where $\mathbf{F}(\mathbf{x}(t))$ describes how the concentrations change as a function of the concentration of a given species, and $\mathbf{L}(\mathbf{x}(t))$ describes how the concentration changes as a function of either the upstream mRNA or the repressing protein.

3.1.2 Oscillator Types

Oscillator networks, particularly those found in gene regulatory or neurological systems, can be directly modeled from data, as we do in the analysis of various genomics and EEG time series data; however, in addition to empirical approaches, we may also explore theoretical models of such oscillators. We turn to the following well known oscillators:

- Van der Pol (VP):

$$
\frac{d^2x}{dt^2} - \mu(1-x^2)\frac{dx}{dt} + x = 0 \qquad \text{or} \qquad \begin{cases} \frac{dx}{dt} &= \mu \cdot (1-y^2) \cdot x - y \\ \frac{dy}{dt} &= x \end{cases} \tag{22}
$$

- Goodwin (GW):

$$
\begin{cases} \frac{dx_1}{dt} &= \frac{\alpha}{1+x_3^n} - x_1 \\ \frac{dx_2}{dt} &= x_1 - x_2 \\ \frac{dx_3}{dt} &= x_2 - x_3 \end{cases} \tag{23}
$$

- Andronov-Hopf (AH):

$$
\begin{cases} \frac{dx}{dt} &= ax - by - x(x^2+y^2) \\ \frac{dy}{dt} &= bx + ay - y(x^2+y^2) \end{cases} \tag{24}
$$

The VP oscillator was introduced to model vacuum tubes by the Dutch physicist Balthasar van der Pol (1889-1959). This second order differential equation and its representation as a pair of first-order differential equations via the Koopman theory, is a non-conservative oscillator that exhibits nonlinear damping. This model has since been applied to study several other biological and complex systems, such as neurological circuit action potentials, and has been well studied [30, 86, 44].

The GW oscillator, proposed by biologist Brian Goodwin (1931-2009), was an early model of a genetic oscillator with three variables to model concentrations of RNA, proteins, and a final product all produced from the same gene and regulated with negative feedback [40, 39, 38]. During the early to mid-1960s, this model of a genetic oscillator was proposed shortly after François Jacob and Jacques Monod introduced their model of gene regulation [52, 53]. The oscillator was developed as a Hamiltonian system and an early proponent of using Hill functions in biology.

The AH oscillator, after the physicist Aleksandr Andronov (1901-1952) and astronomer and mathematician Eberhard Hopf (1902-1983), as shown in eq. (24) is one particular form of a general AH oscillator, which is a pair of coupled oscillators x and y that exhibit a Hopf bifurcation (when a critical point becomes a limit cycle); the Hopf bifurcation is sometimes also referred to as the Poincaré-Andronov-Hopf bifurcation to provide attribution to Poincaré and Andronov for their work studying this bifurcation [93, 77, 76, 48]. Numerous other ecological and biological oscillators, such as the Lotka–Voltera, SIR, Hodgkin–Huxley models, exhibit Hopf bifurcations as well [123, 45, 3].

Internal Oscillator Parameters. The trajectories an oscillator networks are governed in part by the internal dynamics of each oscillator and governed by parameters associated with individual oscillators.

The VP oscillator is governed by the parameter μ, which controls the stability and limit cycle of the system. In fig. 4.A, the dynamics of three instances of the VP oscillator are shown. As μ changes from less than zero to greater than zero, the limiting behavior of the system changes from the origin being a stable critical point to a limit cycle with fast and slow transitions. The top row in fig. 4.A plots the position x in terms of time, and the stability of this system is interpreted based upon how the oscillation amplitud changes between periods. For instance, in the top row where $\mu < 0$, the contraction of the position over time indicates stability. The bottom row of in fig. 4.A plots the position x relative to the velocity y, and there the stability can be seen based upon the shift of the trajectory toward the origin or in a stable limit cycle. For instance, in the bottom row where $\mu > 0$, the trajectory enters a limit cycle where it remains throughout time, similar to the fixed amplitude oscillations above. By varying μ, the behavior of individual oscillators is changed and can effect the dynamics of an oscillator network.

The GW oscillator of eq. (23) is governed by two parameters α and n. Often, and in the original parlance of Goodwin, this oscillator is written with more parameters, but the number of free parameters may be reduced to 2 in the case of equal degredation rates when written in the dimensionless form, which is useful for the purpose of considering its birfurcation behavior [121]. Regardless, the Goodwin oscillator exhibits a Hopf bifurcation, as illustrated in fig. 4.B. By fixing the value of n, and

varying α, we see that for certain values of α the system is stable while other values produce internal oscillations in the form of a limit cycle. The AH, VP, Liénard, and many other oscillators exhibit Hopf bifurcations as well.

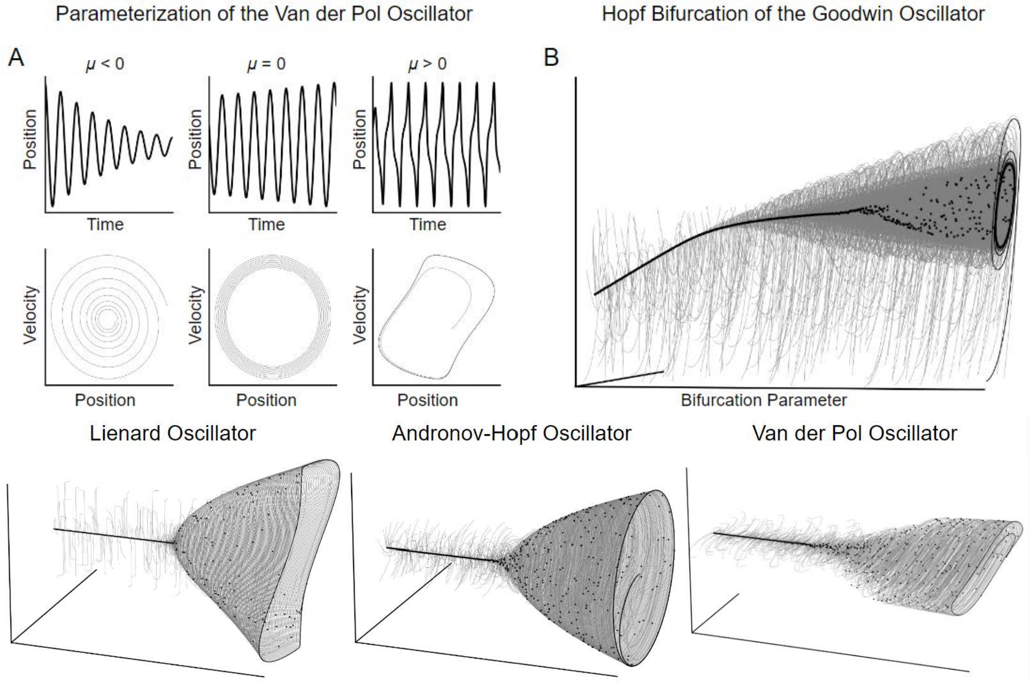

Figure 4: **(A)** The dynamics of a single VP oscillator depend on its parameter μ. The top row shows the time parameteric state of the oscillator where x from the left of eq. (22) is shown as a function of time and the bottom row plots the position x relative to the velocity y based on the right side of eq. (22). **(B)** We fix $n = 20$ and vary the bifurcation parameter α from 0.5 to 2. For each value of α, we simulate a random trajectory, shown in grey, of the oscillator, and plot the end point of the trajectory in black. On the left hand side of this plot, all the trajectories arrive at a final point near each other indicating stability as a fixed point, and on the right hand side, the black dots and end of the trajectories are not necessarily near each other but rather positioned along a limit cycle. This transition occurs near 1.6 and demonstrates the existance of a Hopf bifurcation. Similar examples of the Hopf bifurcation on the AH, VP, and Liénard system are shown below.

3.1.3 Diffusion and Strong Coupled Oscillators

The trajectories of oscillator networks are governed in part by the network structure and external interactions between oscillators. Diffusive coupling of oscillators forces the state of an oscillator toward the state of its neighboring osillators. In particular, when the oscillators are coupling on a graph $\mathcal{G}$ with a Laplacian $\mathbf{L}$, the diffusive

coupling of a single oscillator $\mathbf{x}_i$ can be written as

$$\mathbf{L}(\mathbf{x}_i) = \lambda \sum_{j \in \mathcal{N}(i)} \mathbf{x}_i - \mathbf{x}_j, \tag{25}$$

where $\mathcal{N}(i)$ is the neighbors or oscillators that are adjacent to oscillator i on the graph $\mathcal{G}$, λ denotes the coupling strength, and the vector $\mathbf{x}_i$ represents all state variables of the oscillator.

Returning to fig. 1, there are three VP oscillators coupled in a directed chain. Each of these oscillators has the internal dynamics $\mathbf{F}(\mathbf{x}_i)$ of eq. (22), and the chain of interactions in fig. 1.D defines the graph laplacian $\mathbf{L}(\mathbf{x})$ two edges, from oscillators $\mathbf{x}_1$ to $\mathbf{x}_2$ and from $\mathbf{x}_2$ to $\mathbf{x}_3$. The full dynamics of this system are written as

$$\begin{aligned}
\frac{dx_1}{dt} &= \overbrace{\mu_1 \cdot (1 - y_1^2) \cdot x_1 - y_1}^{\mathbf{F}(\mathbf{x},\mu)} \quad \overbrace{-0 \qquad\qquad}^{-\mathbf{L}(\mathbf{x})}, \\
\frac{dy_1}{dt} &= x_1 \quad -0 \quad, \\
\frac{dx_2}{dt} &= \mu_2 \cdot (1 - y_2^2) \cdot x_2 - y_2 \quad -\lambda(x_2 - x_1), \\
\frac{dy_2}{dt} &= x_2 \quad -\lambda(y_2 - y_1), \\
\frac{dx_3}{dt} &= \mu_3 \cdot (1 - y_3^2) \cdot x_3 - y_3 \quad -\lambda(x_3 - x_2), \\
\frac{dy_3}{dt} &= x_3 \quad -\lambda(y_3 - y_2).
\end{aligned} \tag{26}$$

Here, there are three Van der Pol oscillators written in their two dimensional form $\mathbf{F}(\mathbf{x}, \mu)$, each with its on internal state $\mathbf{x}_i = [x_i \quad y_i]^\top$, and internal parameters μ_i discussed in the prior section. In eq. 26, x_1 and y_1 are not influenced by external parameters from other oscillators, so the long term behavior of the oscillator network is controlled by oscillator 1.

From Lin's perspective of structural observability/control, oscillator 1 is called a root node. This means all other nodes can be influenced from oscillator one and it is uneffected by any node. As a result, from the perspective of structural observability/control, oscillator 1 is a good sensor/actuator. This is based on the notion that the LTI system defined on the adjacency matrix of the graph from fig. 1.D is observable/controllable with oscillator 1 as a sensor/actuator according to the PBH test.

One factor not considered by Lin's structural perspective is the strength or weight of the coupling between oscillators. In eq. 26, all oscillators are coupled with an identical parameter $\lambda > 0$, but in practice, each interaction could have its own coupling strength. Since coupled oscillators interact by adjusting their phase relative to one another, the coupling coefficients λ, control both the speed and direction with which oscillators interact [51].

In fig. 5 and fig. 6, for instance, several instances of coupled VP and AH oscillators are shown. We consider oscillator networks containing either 2 or 5 oscillators coupled in a ring or cycle network where all interactions have a weight λ. In the first two rows, we consider the case where $\lambda_1 = 1$ and $\lambda_2 = -1$ such that if the oscillators were uncoupled, one would decay quickly toward the origin and one would exhibit a limit cycle. In the top row, $\lambda > 0$ such that the oscillator in the limit cycle induces synchronized oscillations in the otherwise stable oscillator, and in the second row, where $\lambda < 0$, so that the induced oscillations of the stable VP oscillator are out of phase with the oscillator in the limit cycle. Rows 3 and 4 recreate rows 1 and 2 where both oscillators are parameterized with $\mu > 0$ so that both oscillators exhibit limit cycles. Here the coupling of λ synchronizes the oscillations rather than induces new ones, and comparing between the left and right columns where the magnitudes of λ change, we see that as the coupling strength increases, the oscillators shift into synchrony or phase locking quicker. Finally, rows 5 and 6 recreate identical results as in rows 3 and 4, with an expanded number of oscillators so that the synchronization and phase locking is more clear.

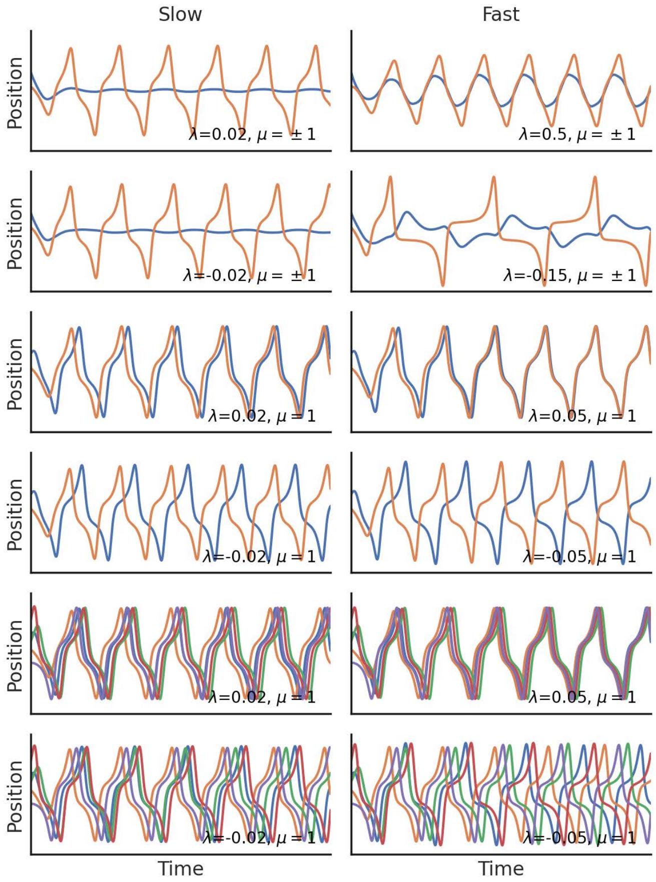

Figure 5: Coupled VP oscillators are used in the above 12 simulations. The oscillators are coupled in rings, and rows 1-4 have networks of 2 oscillators whereas rows 5 and 6 have 5 oscillators. Each row illustrates one type of dynamics, where the left column illustrates weak coupling between the oscillators and the right column illustrates strong coupling, governed by the parameter λ. Row 1: an oscillator with $\mu = 1$, which alone exhibits a limit cycle, is coupled with an oscillator where $\mu = -1$ that would decay. The oscillating node induces synchronized oscillations in the network. Row 2: Similar set up as row 1, now with $\lambda > 0$ so that phase locking is induced. Row 3: synchronization between 2 oscillators nodes. Row 4: Phase locking between 2 oscillating nodes. Row 5: synchronization between several oscillators. Row 6: phase locking between several oscillators.

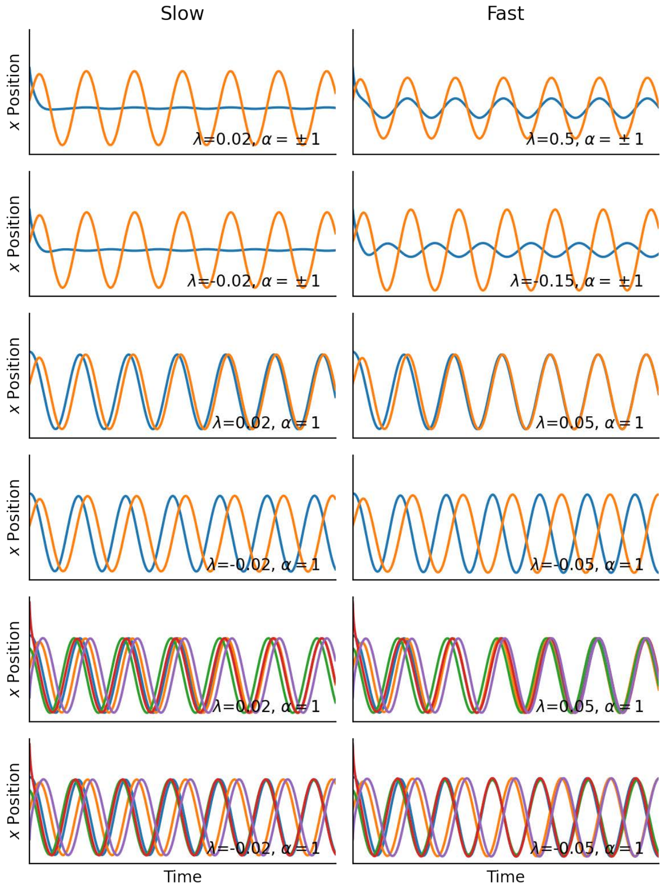

Figure 6: Coupled AH oscillators are used in the above 12 simulations. Whereas VP oscillators have a single position variable x, AH oscillators have two variables x and y. The behavior is qualitatively similar between the x and y variables, so only the position of the x variables for each oscillator is shown in these simulations. The interpretation of these twelve plots mirrors that of the corresponding simulations of VP oscillators detailed in fig. 5

3.2 Sensor Selection Formulations

Based on the oscillator networks as a guiding example and the reviewed methods of observability in section 2, here we propose two methods for dynamically selecting sensors. Consider the system

$$\begin{cases} \mathbf{x}(t+1) & = \mathbf{A}(t)\mathbf{x}(t) \\ \mathbf{y}(t) & = \mathbf{C}(t)\mathbf{x}(t), \end{cases} \tag{27}$$

which is equivalent to eq. (6) without control. We summarize two methods of sensor selection and designing $\mathbf{C}$ for eq. (27), which are extensions of similar methods for LTI systems.

- **Energy based selection:** One graded approach to maximize observability is to maximize the output energy of the system, formalized as:

  $$\max_{\mathbf{C}(t)} \mathcal{E} = \max_{\mathbf{C}(t)} \mathbf{x}(0)^\top \mathbb{G}\mathbf{x}(0) \text{ subject to } \mathbf{C}(t)\mathbf{C}(t)^\top = \mathbf{I}. \tag{28}$$

  A similar formulation with LTI dynamics with fixed outputs was used by Hasnain et. al. to select biomarkers [47]. This maximization problem is a quadratic program with linear constraints, and particularly when $\tilde{\mathbf{A}}$ has been computed from DMD, the sensor rankings from this optimization can be obtained quickly.
- **Gramian based selection:** Sensors can be selected to optimize a particular function of the Gramian that corresponds to the observability of the system. We consider the maximization

  $$\max_{\mathbf{C}(t)} J(\mathbb{G}), \tag{29}$$

  where J is one of the five functions discussed in section 2.2.2. Summers et. al. utilized this formulation for fixed sensor selection on LTI systems [109]. The five proposed functions J of the Gramian are submodular, making them conducive to greedy algorithms for sensor selection, and when the trace is used, eq. (29) is solved with a linear program.

Since the objective functions of the optimization problems in eq. (28) and eq. (29) are continuous, (C3) is addressed by providing scalar value of observability rather than a binary criteria. Moreover, since both optimizations allow for the use of model reduction, and can be solved with fast algorithms to address (C2). In the subsequent sections, we provide further detail regarding how to solve these optimization problems and select sensors.

3.3 Output Energy Maximization

Here we provide our method to solve eq. (28) based on its Lagrangian dual form. We first discuss how this problem is solved when the sensors $\mathbf{C}$ are fixed for all time and then consider the dynamic selection of sensors.

3.3.1 Time Invariant Sensors

The objective is to select sensors $\mathbf{C}$ that maximize the signal or output energy of the system $\mathcal{E}$ where

$$\mathcal{E} = \sum_{i=t_0} \mathbf{y}(i)^\top \mathbf{y}(i) = \sum_i \mathbf{x}(0)^\top \Phi(i,t_0)^\top \mathbf{C}^\top \mathbf{C} \Phi(i,t_0)\mathbf{x}(0),$$

which is formalized as

$$\max_{\mathbf{C}} \mathcal{E} \text{ subject to } \mathbf{C}^\top \mathbf{C} = \mathbf{I}. \tag{30}$$

Equation (30) is the fixed sensor formulation of eq. (28). Hasnain et. al. shows that the Lagrangian dual formulation of this problem is

$$\max_{\mathbf{C}} \mathcal{E} + \mathcal{L} \text{ where } \mathcal{L} = \mathbf{tr}((\mathbf{C}\mathbf{C}^\top - \mathbf{I})\mathbf{D}),$$

where $\mathbf{D}$ are the dual variables [47]. Following eq. 5 of [47],

$$\frac{\partial(\mathcal{E}+\mathcal{L})}{\partial \mathbf{C}^\top} = 2\mathbf{G}\mathbf{C}^\top - 2\mathbf{C}^\top\mathbf{D} = 0, \text{ such that } \mathbf{G}\mathbf{C}^\top = \mathbf{C}^\top\mathbf{D}. \tag{31}$$

This last expression implies that the eigenvectors of $\mathbf{G}$ are the sensor weights or the importance of each sensor at a critical point of the signal output energy with respect to the sensors. We extend the approach to the selection of time varying sensors.

3.3.2 Time Variant Sensors

Suppose we seek to maximize the signal energy at time t such that our objective function is $\mathcal{J}_t$ where

$$\mathcal{E}(t) = \mathbf{y}(t)^\top \mathbf{y}(t) = \mathbf{x}(0)^\top \Phi(t,t_0)^\top \mathbf{C}(t)^\top \mathbf{C}(t)\Phi(t,t_0)\mathbf{x}(0). \tag{32}$$

Applying the prior Lagrangian formulation here, we seek to maximize $\mathcal{E}(t)$ with respect to $\mathbf{C}(t)$ at all times. Since $\mathbf{C}(t_1)$ and $\mathbf{C}(t_2)$ are independent of one another for all t_1 and t_2, we can maximize the energy at each time $\mathcal{E}(t)$ independently of one another. To do so, we extend the fixed sensor selection optimization of $\mathcal{E}$ to an optimization of $\mathcal{E}(t)$, using a similar approach as in [47]. This is formulated as follows:

$$\begin{aligned}
\frac{\partial(\mathcal{E}_t+\mathcal{L})}{\partial \mathbf{C}(t)^\top} &= \frac{\partial}{\partial \mathbf{C}(t)^\top}\Big(\mathbf{x}(0)^\top\Phi(t,t_0)^\top\mathbf{C}(t)^\top\mathbf{C}(t)\Phi(t,t_0)\mathbf{x}(0) - \mathbf{tr}((\mathbf{C}(t)\mathbf{C}(t)^\top - \mathbf{I})\mathbf{D})\Big) \\
&= \frac{\partial}{\partial \mathbf{C}(t)^\top}\Big(\mathbf{tr}\big(\mathbf{x}(0)^\top\Phi(t,t_0)^\top\mathbf{C}(t)^\top\mathbf{C}(t)\Phi(t,t_0)\mathbf{x}(0)\big) - \mathbf{tr}((\mathbf{C}(t)\mathbf{C}(t)^\top - \mathbf{I})\mathbf{D})\Big) \\
&= \frac{\partial}{\partial \mathbf{C}(t)^\top}\Big(\mathbf{tr}\big(\mathbf{C}(t)\Phi(t,t_0)\mathbf{x}(0)\mathbf{x}(0)^\top\Phi(t,t_0)^\top\mathbf{C}(t)^\top\big) - \mathbf{tr}((\mathbf{C}(t)\mathbf{C}(t)^\top - \mathbf{I})\mathbf{D})\Big).
\end{aligned} \tag{33}$$

At this point, let

$$\mathbf{G}(t,t_0) = \Phi(t,t_0)\mathbf{x}(0)\mathbf{x}(0)^\top\Phi(t,t_0)^\top.$$

Then, returning to eq. (33) we can obtain the optimal sensors $\mathbf{C}(t)$

$$\begin{aligned}\frac{\partial(\mathcal{E}_t+\mathcal{L})}{\partial\mathbf{C}(t)^\top} &= \frac{\partial}{\partial\mathbf{C}(t)^\top}\Big(\mathbf{tr}\big(\mathbf{C}(t)\mathbf{G}(t,t_0)\mathbf{C}(t)^\top\big) - \mathbf{tr}((\mathbf{C}(t)\mathbf{C}(t)^\top - \mathbf{I})\mathbf{D})\Big)\\ &= 2\mathbf{G}(t,t_0)\mathbf{C}(t)^\top - 2\mathbf{C}(t)^\top\mathbf{D} = 0.\end{aligned} \tag{34}$$

This has a similar interpretation to the time invariant case, were the eigenvalues of $\mathbf{G}(t,t_0)$ denote the contribution of each state variable to observability at time t.

To solve this and select sensors from time $t_0,\ldots,t$, we must form $\mathbf{G}(t,t_0)$ for all t. This requires integrating the system forward from the initial conditions $\mathbf{x}(0)$, which can be performed efficiently using model reduction, and then computing the largest eigenvector of the matrices $\mathbf{G}(t,t_0)$, for which there are fast algorithms.

3.4 Gramian Based Observability

Here we provide our method to solve eq. (29) with standard optimization techniques. We begin by highlighting a few key properties of the observability Gramian for LTV systems, then provide the integer programming formulation of eq. (29) as an integer programming. Finally, we provide a continuous relaxation of the integer programming problem, making it solvable as a linear program.

3.4.1 Integer Programming Formulation

To formulate the sensor selection problem in terms of integer programming, consider the case where there is a binary variable denoting whether or not each state variable $x_i \in \mathbf{x}$ is observed or measured at time t. Let $\mathbf{c}_{tj}$ be the j-th row of the matrix $\mathbf{C}_t \in R^{n\times p_t}$. Then the LTI observability Gramian can be reweitten as

$$\begin{aligned}\mathbb{G}_o &= \sum_{i=0}\Phi(i,0)^\top\mathbf{C}(i)^\top\mathbf{C}(i)\Phi(i,0),\\ &= \sum_{i=0}\sum_{j=1}^{p_k}\Phi(i,0)^\top(\mathbf{c}_{ij})^\top\mathbf{c}_{ij}\Phi(i,0).\end{aligned}$$

Let, $\alpha_{tj} \in \{0,1\}$ be a binary variable, which indicates whether x_j variable is measured at time t. Then one can express above relation, as

$$\mathbb{G}_o(\alpha) = \sum_{i=0}\sum_{j=1}^{n}\alpha_{ij}\mathbf{W}_{ij},$$

where, $\alpha = (\alpha_{11},\alpha_{21},\cdots,\alpha_{(T+1)n})^\top \in R^{n(T+1)}$, where T is the upper bound on the first summation used to compute the observability Grammian, and

$$\mathbf{W}_{ij} = \Phi(i,0)^\top(\mathbf{c}_{ij})^\top\mathbf{c}_{ij}\Phi(i,0).$$

Within this representation, note that $\mathbf{c}_{ij}$ is a row vector with j-th entry as 1 and zero otherwise. Note that each $\mathbf{W}_{ij}$ is a positive semidefinite matrix. Because composition with affine mappings preserves convexity, each measure $J_i(\mathbb{G}_o)$ discussed in the Section 3.4 is also a convex function $J_i(\mathbb{G}_o(\alpha))$ of the sensor selection variable α.

Given the sensor selection variables α, the sensor selection problem can be written as a mixed-integer convex problem,

$$\min_{\alpha} J(\mathbb{G}_o(\alpha)) \quad \text{subject to} \quad \sum_{j=1}^{n} \alpha_{tj} \leq p_t, \quad t = 0, 1, \cdots \quad \text{where} \quad 0 \leq \alpha_{kj} \in \{0, 1\}. \tag{35}$$

Here, the first constraint restricts number of selected sensors to be no more than p_t for each time point. Because mixed-integer programs do not scale well for large problems, a convex relaxation to (35) provides a useful solution alternative.

3.4.2 Continuous Relaxation

In the continuous relaxation, the observation of variable j at time t is relaxed to the interval $\alpha_{ij} \in [0, 1]$. This leads to the convex program,

$$\min_{\alpha} J(\mathbb{G}_o(\alpha)) \quad \text{subject to} \quad \sum_{j=1}^{n} \alpha_{tj} \leq p_t, \quad t = 0, 1, \cdots, \quad \text{where} \quad 0 \leq \alpha_{tj} \leq 1. \tag{36}$$

The advantage of the relaxation is that it can be solved in time that is polynomial in the number of variables using efficient techniques such as interior point methods. Furthermore, if the solution to the relaxed problem is such that $\alpha_{ij} \in \{0, 1\}$ (within numerical tolerance), then the original mixed-integer problem has been solved. The relaxation serves two roles — an approximate (suboptimal) solution to the mixed-integer problem by rounding α_{ij}, and, in some cases, a fast optimal solution to the mixed-integer problem.

In both the mixed-integer problem (35) and the convex relaxation (36), the desired number of sensors was explicitly constrained to be p_t. Another approach is to allow the number of sensors to be a free variable, and enforce a sparse solution, which can be achieved, for example, by l_1 regularization technique which yields a convex problem,

$$\min_{\alpha} J(\mathbb{G}_o(\alpha)) + c\|\alpha\|_1 \quad 0 \leq \alpha_{tj} \leq 1, \tag{37}$$

where the constant $c \geq 0$ is the weighting on the l_1-norm penalty. By varying the weight c, the number of sensors in the solution set will change to balance the sparsity penalty with the observability measure, tracing the Pareto tradeoff curve between sparsity and observability can be used to achieve a particular level of observability.

4 Structure Guided Sensor Selection

For many systems where the exact network edge structure is unknown or partially known, we can supplement our understanding of the system from structure among the nodes that extends beyond the adjacency structure of the network. For instance, in constructing social, telecommunications, or postal networks, where the contacts of only some individuals are known, the locations of individuals is informative to network structure [79, 122, 36, 89]; and given our knowledge of the transcription cluster and chromosome territories, the positioning of genes on different chromosomes is important for understanding gene regulatory networks [46, 24]. In each case, we can assign location/positional attributes to each node that form an underlying structure between the nodes, independent of the edge or adjacency structure on the network.

4.1 Small World Networks

Small World networks, characterized by relatively small diameters (distance across the network) and high clustering coefficients (similarity between nodes), are often formed from an underlying structure among the nodes. In 1967, Stanley Milgram's famous experiment, from which the Small World and related six degrees of separation theories stem, mapped the first Small World network by considering the social network between Nebraska and Boston [79, 116]; the location of individuals around the United States informed the social network through which packages were mailed to friends and acquaintances. Subsequently, in 1998, Duncan Watts and Steve Strogatz proposed a tunable model, dubbed the Watts-Strogatz model (WS), for generating networks with Small Worlds properties [117]. This model introduces long range connections, or "weak ties" to highly structured lattices [42, 41]. The initial lattice structure, resembling spatial distribution, serves as the framework for forming Small World networks. Small World properties have since garnered recognition across a variety of fields where both structural and functional or relational data are captured [42, 88, 56, 108, 90, 29, 11, 10].

Considering the significance of structure in Small World networks and their ubiquity, we study the observability and effective sensor selection methods for such networks. We present results from two simulation to investigate the observability and quality of sensor placement on highly structured, Small World , and random networks, focusing on scenarios where only the lattice or spatial structure is known.

4.1.1 Sensor Ranking Distribution from Lattices

Suppose the lattice structure used to generate a Small World network but not the rewired, long-range interactions exist in a Small World network formed from the WS model.

What can the lattice tell us about the expected ranking of each node as a sensor in the Small World network?

To investigate this question, we considered the following model. Small World networks were generated according the the SW model (WattsStrogatz in algorithm 1) using the lattice structures in fig. 7. The unweighted, bidirectional adjacency

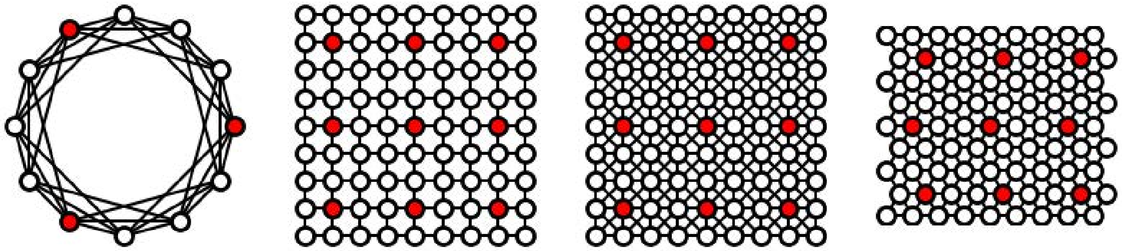

Figure 7: Lattice structures used to create Small World networks are shown with evenly spaced sensor nodes (red) placed across the network. Four types of lattices are shown: (1) ring with 12 vertices, degree of 6, and 3 sensors, (2) 9×9 grid with connections to 4 nearest neighbors and 9 evenly spaced sensors, (3) 9×9 grid with connections to 8 nearest neighbors and 9 evenly spaced sensors, and (4) 9×9 isometric grid with 9 evenly spaced sensors.

matrix $\mathbf{A}$ from each network was used in place of $\mathbf{A}_t$ in eq. (1) or eq. (6). The value of each node as a sensor was evaluated based upon their contribution to observability as defined by section 3.4.2.

Based upon this model, the expected contribution of each node as a sensor to observability in a small world network mirrors its impact on observability within the lattice structure. In fig. 8, the sensor rankings of each node on the lattice are juxtaposed with the average sensor ranking over 2500 instances of Small World networks generated from the lattice as well as the sensor rankings on a single Small World network. Qualitatively, the anticipated sensor contributions of each node closely resemble those observed on the lattice.

To asses and provide a scale for the utility of the lattice structure in this sensor selection, we measured the similarity in the sensor selection on the Small World and random networks relative to the lattice structures. As a baseline, we utilized the Erdős-Rényi-Gilbert (ERG) model for random graphs to ensure that the lattice, Small World , and reference point shared identical node and edge counts [27, 34, 28]. We recorded the contribution to observability of each node in a vector and calculated the distance between these vectors using the Frobenius norm, as illustrated, as illustrated in fig. 9.

Across all norms, the square, diagonal, and isometric lattices typically show more similarity to the sensor ranking on the lattice than the random graph, but this is not true of the ring lattice. In this ring lattice, all nodes are equivalent such that the ring lattice is symmetric up to any rotation of nodes; however, in each of the other three lattices, the structure creates center, edge, and even corner nodes, each with different properties such as their degrees or contributions to observability as shown in fig. 8. Similar to the ring lattice, the expected structure of an ERG network is perfectly symmetric. The increased symmetry of the ring lattice explains why across the shown norms, the distributions generated from the ring and random lattices shown in fig. 9 are similar.

Our choice to use the Frobenius norm is arbitrary, so for completion, we evaluate the similarity based upon several additional distances in fig. 24-27. Regardless of

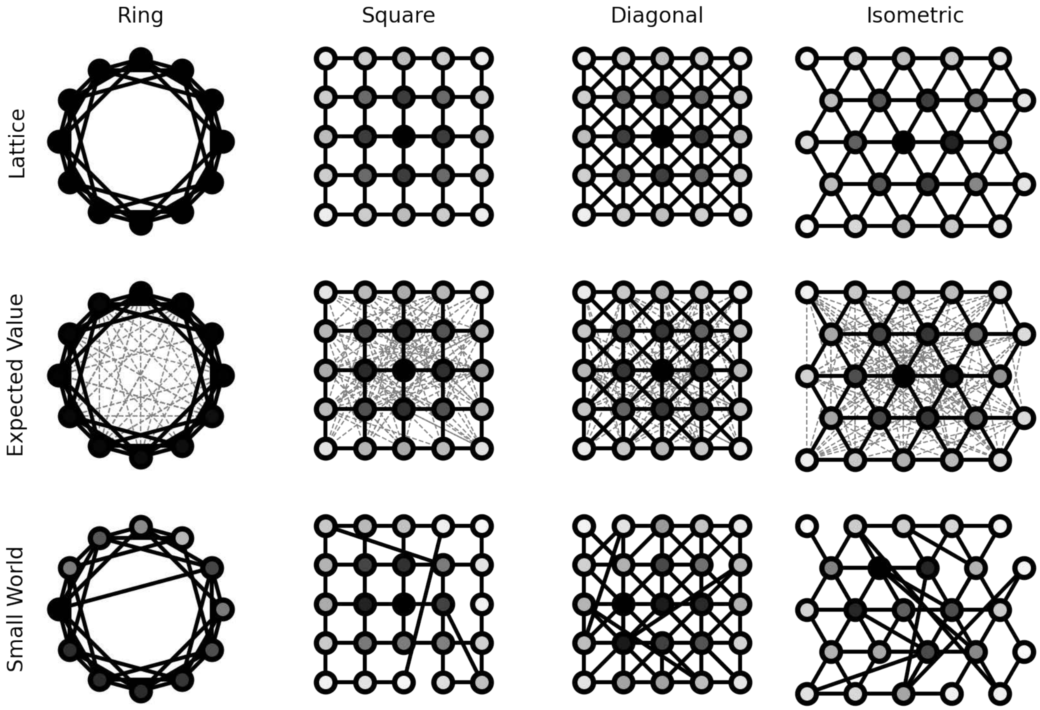

Figure 8: The rank of each node as a sensor on four different lattice structures and average ranking from SW graphs generated from these lattices is shown. Darker intensity indicates high ranking as a sensors. The top row is the sensor ranking on the lattice shown with black edges; the middle row is the average sensor ranking from 2,500 Small World networks generated from the lattice; the bottom row is the sensor ranking for one instance of a Small World network generated from the lattices.

the choice in norm, the Small World and ERG distributions of similarity in sensor rankings relative to the lattice show clear separation, and across all norms except for $\|\cdot\|_1$, the Small World sensors were more similar to the lattice than random.

4.1.2 Observability from Spatially Distributed Sensors

Given that the expected sensor ranking of a Small World network can be determined from the lattice, we consider effective stratigies for placing sensors. In line with the supposition that only the lattic structure is known, we consider the peformance of evenly placed sensors on the lattice, as shown in fig. 7.

When is placing sensors evenly in space an effective strategy?

To investigate this question, we consider the following experiment. Sensors are uniformly distributed in space on a lattice.

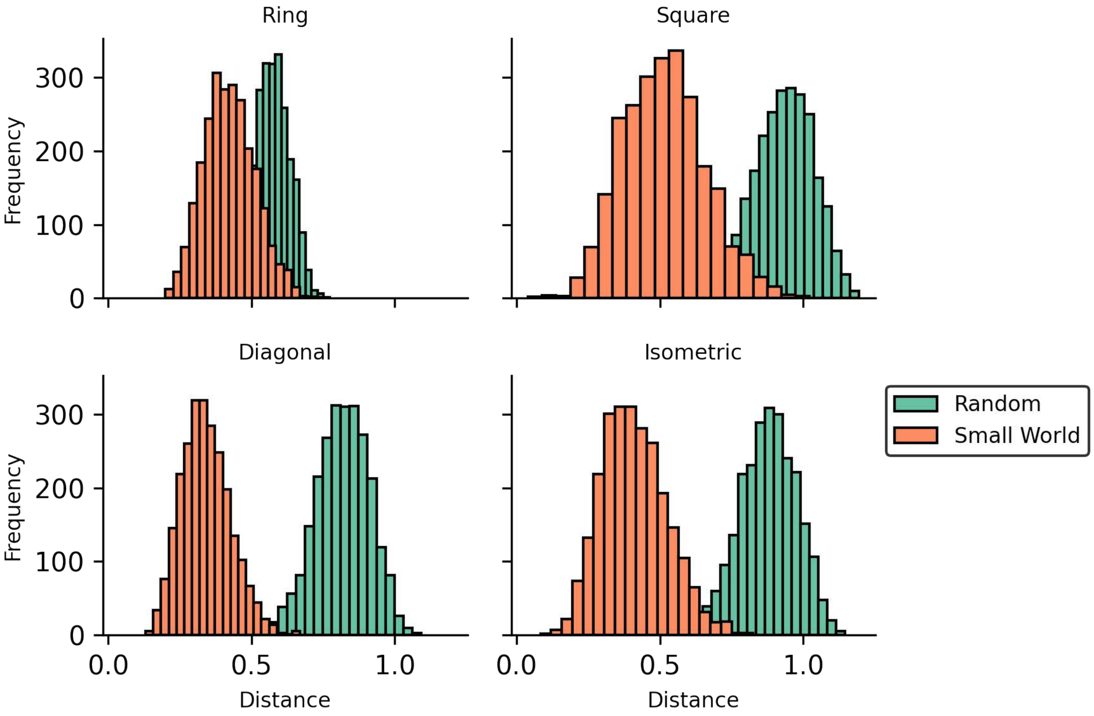

Figure 9: The "distance" in sensor rankings between the lattice and Small World networks generated from the lattices are shown. For scale, the distance between the lattice sensor rankings and sensor rankings from random networks are shown. Sensor ranking distance is computed by representing the contribution of each node to observability as entries in a vector, normalizing each network, and computing the distance between vectors from different networks.

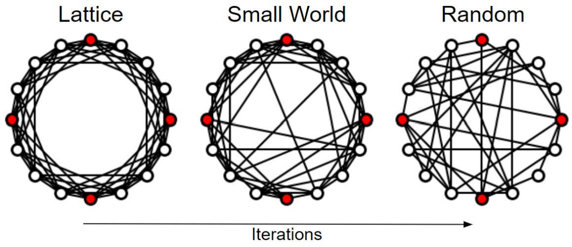

Figure 10: Similar to fig. 1 in [117]; however, here we fix p and change the number of iterations whereas Watts' figure was focused on the variability of p.

Subsequently, while the sensors remain fixed, we conduct multiple iterations of IterativeWattsStrogatz (see algorithm 1) wherein edges are progressively rewired, similar to the WS model. Over multiple iterations, the network structure gradually changes from a lattice, to a small world network (see fig. 10), and finally a random network, and we evaluate the observability from the fixed sensors nodes throughout. As a baseline for comparison, also we measured the observability of the system given by random sensors.

In the early, highly structured phase, the evenly spaced sensors have increased

network observability relative to the randomly spaced sensors, on all latices except for the ring (see fig. 11). As the iterations progress and the network approaches a random graph, the utility of the evenly spaced sensors relative to randomly selected sensors diminishes, and the overall observability of the system decreases. The initially increased observability from the structure guided sensors followed by its decrease suggest there exist a set of networks whose sensor selection can be based on the underlying node structure. The underlying lattice structure alone is not sufficient to guarantee good sensor placement, but it does suggest that enforcing constraints to space out the sensors may be beneficial on small world networks.

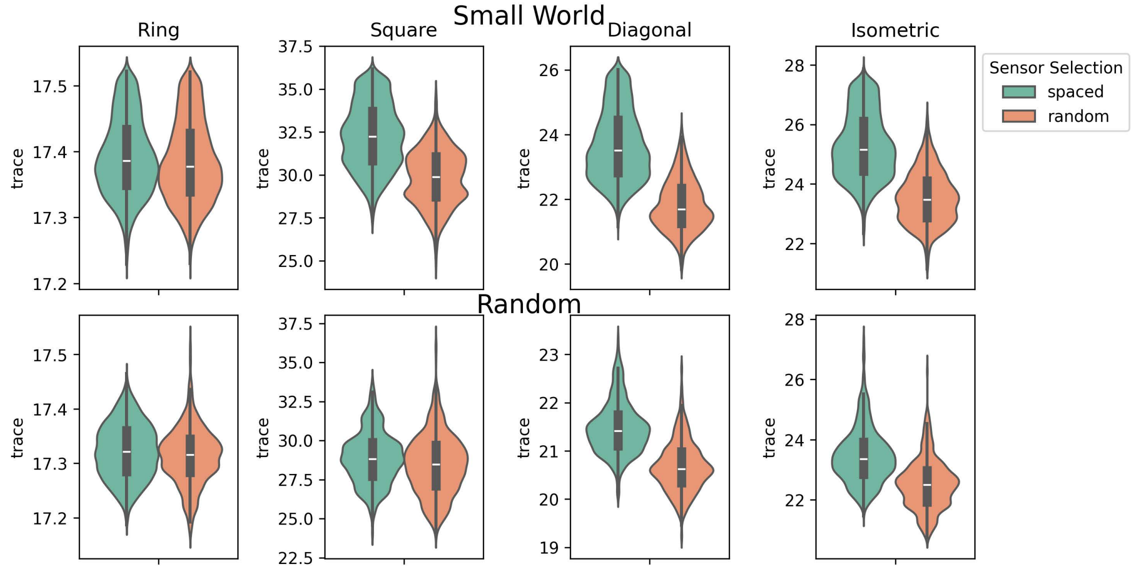

Figure 11: This figure was made based on the four lattice types with 144 sensors each. Evenly spaced sensors on the lattice improve network observability while the networks are Small World (iterations 0-50), and at later time points, the overall observability and the relative gain in observability from evenly spaced sensors on the lattice as opposed to randomly spaced sensors diminishes .

4.2 Constrained Optimizations

The DSS energy and observability Gramian based sensor selection problems (eq. (12) and eq. (29)) can be constrained according to the structure of the system. In the small world example above, we see that placing sensors evenly in space can be an effective strategy for selecting sensors. Here, we modify the DSS optimizations in order to constrain the selection so that at most one sensor per cluster is selected. In principal, these clusters can be created in space or based upon spatial data so that the sensors will be evenly placed in space, but the selection of a clustering method is not of primary importance.

Algorithm 1 Generators for Small World Networks

```
procedure WattsStrogatz(N, k, p)
    G ← Initialize a ring lattice with N nodes, each connected to its k nearest neighbors
    for (u, v) ∈ edges of G do
        if random number < p then
            w ← Randomly select a node (excluding u and its neighbors)
            Remove edge (u, v)
            Add edge (u, w) to G
        end if
    end for
    return G
end procedure

procedure IterativeWattsStrogatz(N, k, p, I)
    G ← Initialize a ring lattice with N nodes, each connected to its k nearest neighbors
    for i = 1, . . . , I do
        rewire each edge with probability p
    end for
    return G
end procedure
```

4.2.1 Linear Constraints in Gramian Optimization

Using the trace of the observability Gramian as a measure of observability, the DSS optimization eq. (29) is formulated as a linear program in eq. (36). In the unconstrained DSS optimization, the only constraint imposed on the linear program is to bound the number of sensor selected. However, we can modify the constraints of this problem based on the spatial clustering of different state variables and solve the optimization problem exactly.

4.2.2 Heuristic Approach for Energy Optimization

To modify the Energy based optimization of eq. (28), we use a heuristic, ranking approach. We first solve the maximization problem of eq. (28) directly and rank the contribution of each sensor at every time. Then, we apply a greedy selection algorithm that selects the top ranked sensor at each time according to their rankings and so as not to violate the rule of selecting multiple sensors per cluster. Pseudocode for this proceedure is provided in algorithm 2.

4.3 Small Worldness of the Genome

Networked models of the nucleus are well established, and the propensity for small world properties have even been seen from molecular dynamics simulations of chromatin conformation [97, 24, 12, 75]. The genome and gene regulatory networks exhibit numerous parallels with classic Small World networks:

Algorithm 2 Energy based SGSS

Given: dynamics $\mathbf{A}_t$ and spatial clusters $C_1, \ldots, C_s$
for each time point t **do**
    place genes into a queue according to their contribution to observability from eq. (28)
    for i=1,...,numSensors **do**
        if the top ranked gene is in a cluster containing no sensors **then**
            select the top ranked gene as a sensor at time t
        end if
        remove the top ranked gene from the queue
    end for
end for

- Similar to Milgram's experiment, our current understanding of the full structure in the nucleus and complete set of regulatory interactions of the genome remains incomplete.
- While our understanding of the system is incomplete, like Milgram, recent experimental assays allow us to partially observe both the structure, the location of individuals in Milgram's experiment or the folding of chromatin in the nucleus, and function, social or gene regulatory interactions, of the genome.
- Similar to the WS model, the genome's architecture arises from the polymer structure of chromatin. The modeling and simulation of such polymers employs a bead-on-a-string model that imposes a one dimensional lattice structure while allowing for distant long range interactions or weak ties to form [58, 85, 18].

Despite these similarities, there has been no empirical quantification of the small-worldness of the nucleus from experimental data. Based upon the observability of Small World networks, to apply SGSS for biomarker identification, we examine Small World properties observed in experimental data by (1) constructing Small World networks with adjacency structures that qualitatively resembles Hi-C and (2) quantitatevly evaluating the small world quotient from Hi-C data.

4.3.1 Overview of Experimental Data

Here we provide a concise discussion of the experimental data types we consider in our study. Hi-C is a genome wide experimental technique to map the spatial arrangement of chromatin within the nucleus [67]. Similar to other Chromosome Conformation Capture methodologies, the Hi-C assay (1) cross-links, (2) digests, (3) ligates, and (4) sequences pieces of DNA to identify proximal genomic loci. The resulting data structure is typically viewed as an occurrence or frequency matrix, where the i, jth element denotes the number of times the ith and jth loci of chromatin were observed near one another.

From a data science or controls perspective, Hi-C is both interesting and challenging to consider because it can be analyzed at multiple resolutions, as shown in

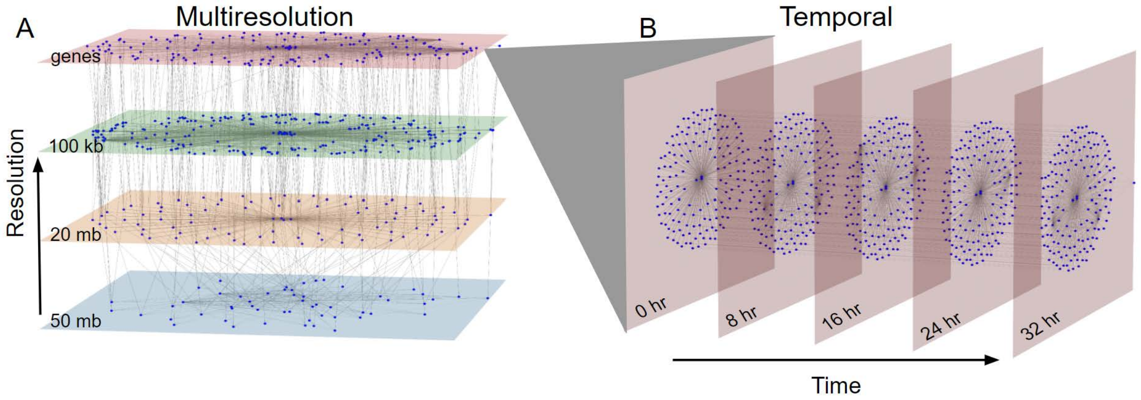

Figure 12: Hi-C data can be viewed as a multilevel or multiresolution network. The state variables of Hi-C and the gene regulatory network is not a one-to-one mapping, but there is a mapping between the two.

fig. 12. Whereas gene expression occurs in discrete units, Hi-C is analyzed by binning or summing over the base pairs that form the chromatin polymers. Examining genome wide Hi-C at base pair resolution is both challenging, as it requires a matrix with $\approx 9 \times 10^{18}$ entries, and unnecessary, as viewing the data at lower resolutions by averaging the number of contacts yields interesting and relevant information. Also, in contrast to eeg or gene expression signals, population Hi-C is less variable throughout time, particularly when the cells are unsynchronized.

In the following sections, we propose and validate models to demonstrate that the genome exhibits Small World properties. However, a point of clarity must be made regarding the relationship between Hi-C and gene expression. The motivation to identify biomarkers for methods such as adaptive sequencing based upon the RNAseq datasets such as Proliferation, Reprogramming, MyogranicSignal, and SWB215 utilizes a gene-centric perspective. From these data, we characterize gene expression dynamics using eq. (1), where the node set or state vector $\mathbf{x}$ represents individual gene expression levels. In contrast, the state representation of Hi-C data classically utilize a basepair-centric perspective, where nodes represent distinct regions of chromatin that may or may not overlap with one or more genes. Consisitent with SGSS and the prior consideration of observing Small World networks, our investigation of the Small World properties of the genome is focused on nuclear structure and not on the gene regulatory dynamics.

4.3.2 Network Model for Hi-C

We propose a simple 4-parameter network model that qualitatively captures the main features found in a single chromosome of Hi-C. Hi-C data are characterized by (1) having strong diagonal dominance, whereby interactions of chromatin are likely to interact with one another, (2) having more distant self-interacting regions, such as Topologically Associated Domains or, at a coarser scale, the arms of a chromosome,

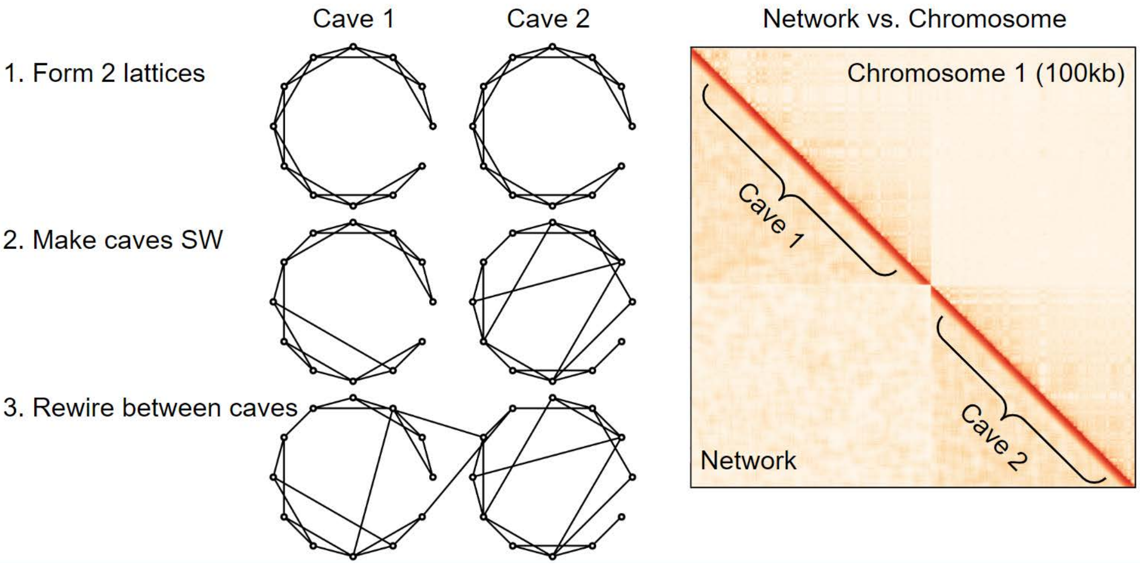

Figure 13: **Small World Network Model for Hi-C.** (left) An outline of the process for forming such networks is shown. First two line lattices are constructed, and each lattice is individual rewired to make it Small World . Then, the lattices are rewired with one another to introduce long range interactions. (right) The network model on the left qualitatively resembles the diagonal dominance, block structure, and weak long range interactions of chromosome 1 at 100 kb. Additional models fit to different chromosomes are shown in fig. 28.

and (3) looping structures where distant loci will have strong interactions that appear far from the diagonal on the matrix.

To recaptiulate these three structures, we utilize a combination of the WS and Caveman models of networks. In a caveman network, nodes are tightly clustered within their cave, and have few interactions between caves. This is similar to the high number of contacts observed within a TAD or arm of a chromosome as compared to more distal regions. In our model, we begin with two line lattices of size n_1 and n_2, rewire interactions on both with probability p_1 according to the WS model, and then rewire interactions between the two now SW networks with probability p_2. The four parameters n_1, n_2, p_1, and p_2 constitute the model, and the process to form this model is shown in fig. 13.

We fit one instance of this model to the individual chromosomes from [16] (see fig. 28). This model performs best, in terms of its qualitative resemblance of the Hi-C network, for the larger chromosomes and when the chromosome arms are relatively equal length. Regardless, this model is able to capture the diagonal dominance, self interacting regions, and spotty long range interactions of Hi-C with relatively few parameters and Hi-C structure.

4.3.3 The Nucleus is a Small World

To empirically validate the hypothesis that the nucleus is a small world, we computed the Small World Quotient (SWQ) for all Hi-C data paired with the PROLIFERATION and REPROGRAMMING datasets. We considered this data at various resolutions, ranging from 10kb to 25mb to, and computed SWQs for the entire genome as well as for individual chromosomes. For individual chromosomes, we used Toeplitz normalization (observed/expected).

To quantify small worldness, the SWQ measures the clustering coefficient and distance across the network relative to random graphs. In particular, the SWQ σ is the ratio of the clustering coefficient and diameter, characteristic path length (CPL), or other distance measure of the Small World network relative to the corresponding statistics of a random network i.e.

$$\sigma = \frac{C/\bar{C}_r}{L/\bar{L}_r}.$$

Here, C and L are the clustering coefficient and diameter of the network and C_r and L_r are the mean clustering coefficient and diameter of random graphs that are a similar size to the tested network. A small world coefficient $\sigma > 1$ indicates small world properties [57, 111, 87].

Computing distances and clustering on Hi-C networks, particularly at very low or very high resolutions, present several challenges. At a high resolution, the number of nodes is relatively large, so computing the exact CPL, diameter, or clustering coefficient can expensive. At low resolution, where there are fewer nodes, the network becomes a weighted, dense, where the sparse, small world structure is not immediately present, so to address this, we threshold the Hi-C to enforce sparsity. After thresholding Hi-C, at various values, we removed all disconnected vertices in the remaining network. We employed a hundred ERG models as a baseline reference used to compute $\bar{C}_r$ and $\bar{L}_r$.

Properties such as the SWQ, distance, clustering coefficients, and the threshold values used are shown in fig. 14 for the Hi-C data from [16, 65]. Thresholds for the minimum number of Hi-C contacts to count as an edge were set independently for each Hi-C matrix in order to include a fixed percent of the observed edges. As a result, the utilized thresholds are inversely proportional to the resolution (top right of fig. 14). Across both datasets, at various resolutions, and various thresholds, Hi-C data exhibits small world properties. Other metrics, such as the clustering coefficient and diameter change in a manner consistent with the resolution and network size.

4.4 Hi-C Guided Biomarker Identification

Based on the prior sections discussing observability in a Small World and the Small World properties of Hi-C, we aim to use knowledge of Hi-C structure to select biomarkers and sensor genes that render gene expression networks observable. Following the red panel of fig. 1, we first learn the structure among our state variables, i.e. genes, from Hi-C, and then propose constraints to DSS.

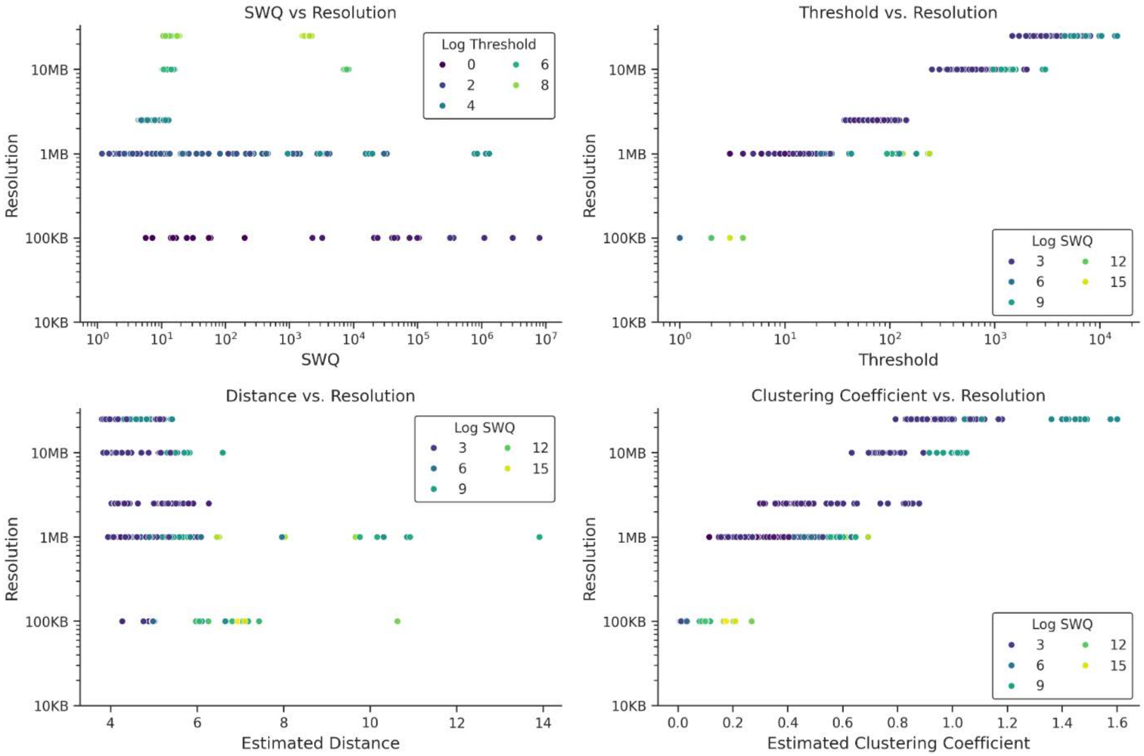

Figure 14: **Small World Properties of Hi-C.** Network properties of Hi-C data including the SWQ (upper left), distance (lower left), and clustering coefficient (lower right) are shown from genome wide Hi-C. These are viewed at multiple resolutions and thresholds in each plot, and the thresholding is performed based upon each resolution (upper right).

4.4.1 Learning Structure from Hi-C

Whereas the Small World networks previously considered assign each node a position in terms of (x, y) or θ, Hi-C data does not explicitly contain the geometry or architecture of the genome. Moreover, the variable resolution of Hi-C measure contacts on a state space that is not a well defined function of the gene state variables whose dynamics we consider. To address these challenges, we propose the two part framework to learn the structure of genes based on Hi-C:

(1) Construct gene resolution Hi-C matrices that indicate the conects between gene coding regions

(2) Utilize clustering as an unsupervised learning approach to identify genes which are spatially similar.

Gene by Gene Hi-C Matrix. To construct a matrix from Hi-C data that both represents the structure of and has nodes for individual genes, we propose GeneX-

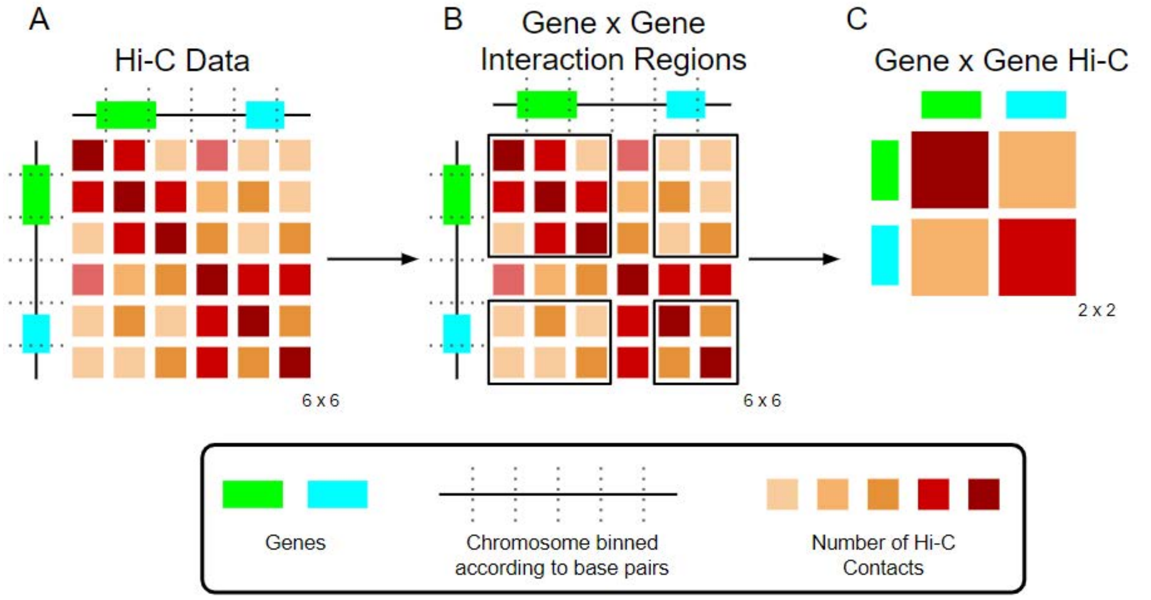

Figure 15: **Constructing Gene by Gene Hi-C Matrices.** Hi-C matrices, processed to any resolution, is constructed so that each Hi-C index or bin represents a fixed length of the genome (left). Based on the gene coding regions, we can identify the segments of Hi-C corresponding to gene-gene interactions (middle). Averaging over these regions, we can construct gene by gene Hi-C matrices where each row/column corresponds to a single gene and a variable length of the linear genome.

GeneHiC (see algorithm 3). Figure 15 illustrates this algorithm, which consists of only two steps. First, each row/column of the Hi-C matrix is assigned gene(s) or the absense of them, based upon the genomic coordinates of the row/column and location of the genes. Rows/columns representing intergenic regions may be assigned no genes, while others might be assigned multiple genes. Subsequently, the average contact frequency of the rows/columns for each pair of genes is recorded in a new matrix. The i, jth entry of this new matrix is the average value in the Hi-C matrix associated with gene i and gene j. The new matrix will have exactly one row/column per gene and can be interpreted in the context of our LTV dynamic models. A similar approach was employed by Chen et. al.; see, for instance [16, fig. S1F].

Learning Spatially Similar Genes. To identify genes with similar spatial characteristics, we clustered them based on a gene-by-gene Hi-C matrix. We employed agglomerative Principal Component (PC) clustering using a Euclidean metric and determined the optimal number of clusters using the Silhouette score. Leading eigenvalues or PCs were used for clustering due to their simplicity and prior implication in chromatin accessibility and epigenetic markers, such as DNAse1, H3K27^{me3}, and H3K36^{me3} [67, fig. 3G].

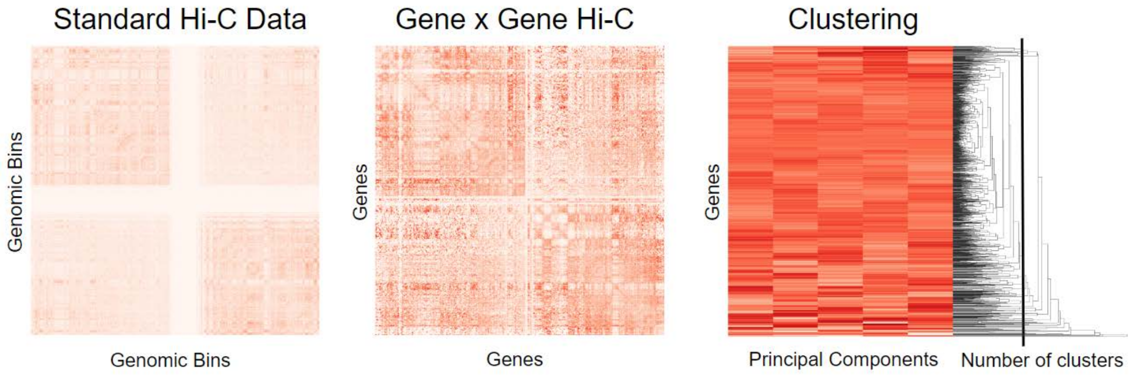

Figure 16: **Gene Clustering from Hi-C.** Standard Hi-C data from chromosome 1 is shown on the left, where genomic bins correspond to a fixed length of chromatin (i.e. 100kb). The gene by gene Hi-C matrix is constructed according the the process outlined above and in fig. 15 (middle). The principal components of the gene by gene Hi-C matrix are used to cluster genes, and the number of clusters is set to maximize the Silhouette score.

This approach to clustering genes from spatial data was selected due to its simplicity, and the end to end procedure is illustrated in fig. 16. For instance, the mathematics required to obtain such clusters amount to only averaging gene counts, the Singular Value Decomposition to compute the PCs, and computing the distance between vectors. While simple and direct, this provides several areas for potential improvement of the clustering approach, including but not limited to:

- Dimension reduction: rather than using PCA for dimension reduction, we could consider alternative methods of dimension reduction
- Improved metric: rather than a Euclidean metric, this approach could be modified or improved by using alternative metrics or Kernel functions to be used for the clustering.

We would like to note limitations of this approach. Namely, the generated clusters are not guaranteed to identify genes that are physically close to one another in space. Moreover, the available Hi-C were not phased, meaning that the expression and spatial features of maternal and paternal alleles are treated equivalently, despite the likelihood that there is great variability of their positioning in the nucleus. Regardless of these limitations, incorporating Hi-C constraints improves the estimation on the Reprogramming and does distribute the sensors across chromosomes, a proxy for distribution in space.

4.4.2 Estimation with SGSS

Due to challenges in estimating the Reprogramming dataset, we applied SGSS based on the above gene clustering approach to identify sensor genes. Due to the similar cell type and improved experimental conditions, we utilized Hi-C from the

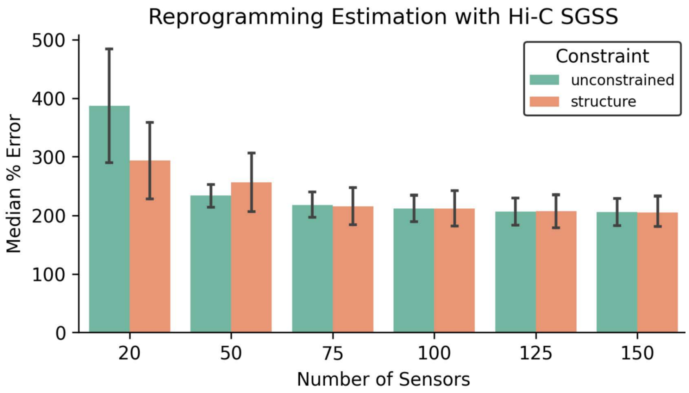

Figure 17: **Estimation of Cellular Reprogramming with SGSS**. We used Gramian based sensor selection and performed estimation on the REPROGRAMMING dataset with and without Hi-C constraints. When only 20 sensors are used, the structure constrained estimation improves the median error of estimating each gene from a factor of 4 to a factor of 3. As the number of sensors increases, the relative utility of these constraints decreases and the methods perform similarly.

PROLIFERATION dataset at 100kb to generate the gene clusters, and we applied the Gramian formulation of DSS.

Performing cross validation, we constructed LTV models using 2 replicates of the REPROGRAMMING data, and tested the models ability to estimate the held out replicate. For a relatively small number of sensors, applying SGSS improves both the average estimation error and reduced the variance in estimation errors. The relative improvement diminishes as more sensors were included in the estimation procedure (see fig. 17), which motivate the further work on the MYOGENICSIGNAL dataset (section 5.2.1).

To validate that SGSS does in fact improve the spatial distribution of biomarkers, we considered the distribution of sensor genes across chromosomes (fig. 17). We built LTI and LTV models of the dynamics and identified the top 2,000 ranked sensor genes from the observability Gramian both with and without Hi-C SGSS. When SGSS, the distribution of sensor genes mirrors the distribution of gene placement on different chromosomes. For example, the disproportionate number of sensor genes are placed on chromosomes 6 and 17 is remedied when the structured constraints are applied. We observed a similar result in improved sensor distribution when selecting time varying sensors as well (fig. 21-23).

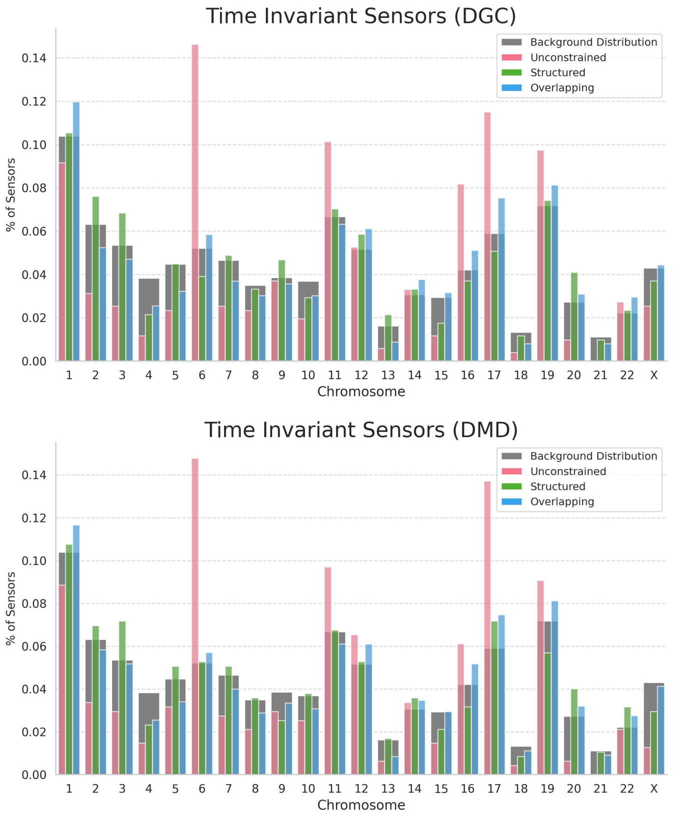

Figure 18: **Placement of Sensor Genes.** We built LTI and LTV models with the DMD and DGC formulations respectively, and we performed Gramian based fixed sensor selection with and without the Hi-C guided constraints to identify the best 2,000 sensor gene ($\approx 10\%$). When sensor selection is constrained by Hi-C, the sensor placement follows a distribution more similar to the background placement of genes across all chromosomes. When no structural constraints are applied, there is great variability so that a disproportionate number of sensors are on chromosomes 6, 11, and 17. Similar figures, where the sensor selection varies with time are shown in fig. 21-23 and illustrate a similar improvement in distribution across chromosomes.

Algorithm 3 Structure Guided Biomarker Identification

```
procedure GeneXGeneHiC(Hi-C Matrix, target genes, gene locations)
    identify Hi-C bins corresponding to each gene
    for each pair of genes i, j do
        H_ij = average interaction in the bins associated with genes i and j
    end for
    Return H
end procedure

procedure GeneClusteringFromHiC(HiC Matrix)
    Form gene resolution HiC matrix H
    Compute truncated SVD of H and threshold to select number of singular vectors for spectral embedding
    Perform agglomerative clustering on spectral embedding
    Return: gene clusters selected according to silhouette score
end procedure

procedure StructureGuidedBiomarker Identification(Time Series RNAseq, Hi-C)
    Cluster genes based on Hi-C matrix
    Learn A(t) based on DMD and DGC
    Evaluate eq. (3) or eq. (5) to selecting at most one gene per cluster
    Return: the selected sensors
end procedure
```

5 Applications to Data

Table 3 summarizes the time series data sets utilized in our study. The sourcing and processing of each dataset is discussed in the sections below. The complete data and associated code will be made available upon acceptance for publication.

Table 3: Time series datasets.

Dataset	Dimension	Time Points	Replicates	Ref.
Proliferation	19 235	8	2	[16]
Reprogramming	19 235	15	3	[69]
myogenicSignal	404	15	3	
SBW25	624	9	2	[47]
EEG	64	160	109	[102]

5.1 Human Fibroblast Proliferation

To investigate the 4D Nucleome, i.e. how the genome architecture and gene expression change over time, Chen *et. al.* generated time series RNAseq and Hi-C data collected at 8 hour intervals in cell cycle and circadian rhythm synchronized human fibroblasts [16]. The experimental protocols to generate this data are available in §Materials and Methods of the appendix of [16]. The FASTQ files from this study were aligned to Homo_sapiens.GRCh38.107 using Bowtie2 with the parameter –very-sensitive. Raw read counts were obtained using HTSeq and converted to transcripts per million (TPM).

5.2 Cellular Reprogramming

To study reprogramming into the myogenic lineage, Liu *et. al.* introduced exogenous MYOD into human fibroblasts, recreating Weintraub's original cell reprogramming experiments [120, 119], and collected paired RNAseq and Hi-C data at 8 hour intervals [69]. The experimental protocols associated with these data are available in the supplement of [69]. The corresponding FASTQ files were obtained and aligned to Homo_sapiens.GRCh38.107 using Bowtie2 with the parameter –very-sensitive. Raw read counts were obtained using HTSeq and converted to TPM.

5.2.1 Myogenic Signal

To generate a reduced dataset amplifying the myogenic signal from the Reprogramming data set, we identified groups of genes involved in myogenesis and cell proliferation, the two dominant biological processes in a reprogramming experiment. Genes related to the cell cycle were taken from the KEGG cell cycle pathway (hsa04110), genes associated with Fibroblasts and Myogenic cells from PanglaoDB, and other

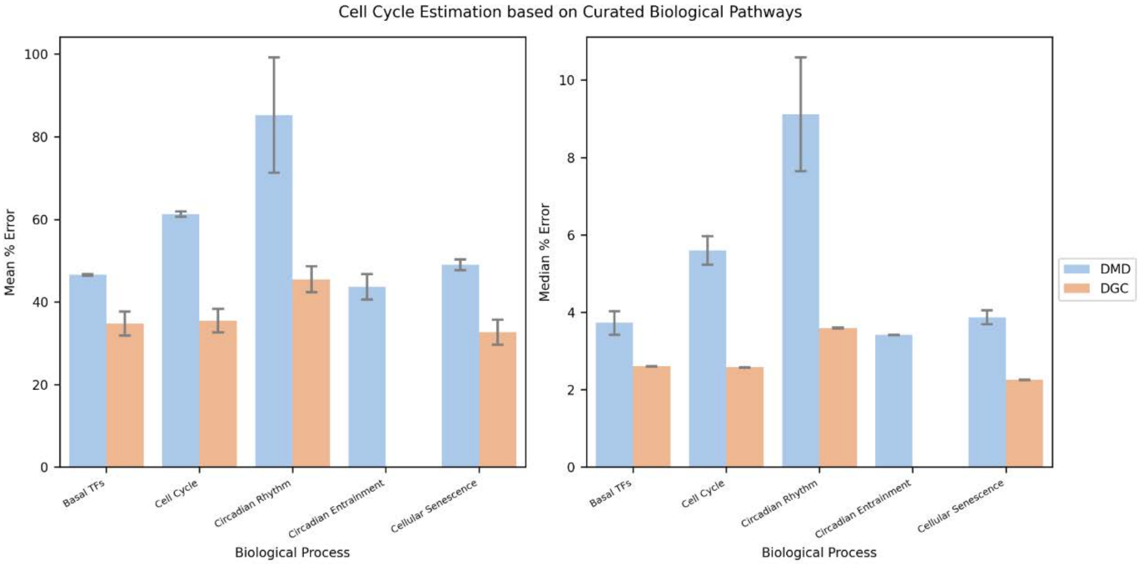

Figure 19: The mean percent error (left) and median percent error (right) are shown for the component wise estimation of the PROLIFERATION dataset. In most instances, the LTV or DGC models outperform the LTI or DMD model with smaller average errors between the multiple replicated. However, when using the Circadian Entrainment sensor set from KEGG with the DGC model, the LTV dynamics fail to produce biologically reasonable estimates, so they are omitted from the figure.

genes known to be involved in cell proliferation and myogenesis. A complete list is shown in Table 4.

5.3 Pesticide Detection in SBW25

To design biomarkers for pesticide detection, Hasnain *et. al.* collected bulk RNAseq of *pseudomonas fluorescens SBW25* every 10 minutes following treatment with the organophosphate malathion, an anti-parasite that is can be used to treat crops or head lice. Beyond being an excellent dataset, this experiment was introduced with the energy based formulation of sensor selection for the purpose of biomarker detection on LTI models of gene expression [47].

Following the quality control and normalization of the original authors, the gene set was filtered to contain only genes with above 100 TPM, leaving approximately 10% of the genes from the original data. These data were obtained and processed directly from the data and codes associated with [47] and accessed from `https://github.com/AqibHasnain/transcriptome-dynamics-dmd-observability`

5.4 Electroencephalogram BCI2000

We studied BCI2000 dataset, a benchmark dataset containing 64 lead EEG data collected from 109 participants performing different tasks [102]. We performed no

ABI3	ANKRD2	CCNB3	CDK4	CXCL3	FGF23	HEY2	MAU2	MYF6	NID2	PDZRN3	RBM20	SPEG	TNS1
ABL1	AQP1	CCND1	CDK6	CXCR4	FGFR4	HGF	MB	MYH1	NIPBL	PKMYT1	RBM24	SPHK1	TNXB
ACHE	ARAP1	CCND2	CDK7	DBF4	FHL2	HHIP	MCM2	MYH11	NKX2-5	PLD1	RBX1	STAG1	TP53
ACTA1	ARHGAP26	CCND3	CDKN1A	DBF4B	FIBIN	HSPB7	MCM3	MYH14	NOG	PLK1	RRAD	STAG2	TP63
ACTA2	ARL4D	CCNE1	CDKN1B	DDX11	FLNC	IGF2	MCM4	MYH4	NOTCH1	PLN	RYR2	STC1	TRDN
ACTC1	ART3	CCNE2	CDKN1C	DES	FMOD	IL11RA	MCM5	MYH6	NOTCH3	PNMT	SCARA5	STK40	TREML4
ACTG2	ASB2	CCNH	CDKN2A	DKK1	FOXF1	IL1R1	MCM6	MYH7	NOX4	POPDC2	SERPINB10	STRN	TRIM63
ACTN2	ATM	CD109	CDKN2B	DLL1	FST	IL4	MCM7	MYH7B	NPHS1	PPP2CA	SERPINB5	SULF1	TRIO
ACTN3	ATR	CD300E	CDKN2C	DMD	FZR1	IL6	MDFI	MYH8	NPPA	PPP2CB	SFN	TAGLN	TRIP13
ADAM12	ATRX	CD36	CDKN2D	DOCK1	GADD45A	ITGA3	MDM2	MYL1	NPPB	PPP2R1A	SGO1	TBX18	TRPV1
ADAM33	AURKB	CDC14A	CDT1	DOCK5	GADD45B	ITGA7	MEDAG	MYL2	NPPC	PPP2R1B	SGPL1	TBX20	TTK
ADAMTS10	BDNF	CDC14B	CHEK1	E2F1	GADD45G	JPH2	MEF2B	MYL3	NT5E	PPP2R5A	SIX1	TBX3	TTN
ADIPOQ	BMP4	CDC16	CHEK2	E2F2	GATA4	KLF4	MEF2D	MYL4	OBSCN	PPP2R5B	SIX4	TCAP	TXLNB
ADIPOR2	BRAF	CDC20	CHODL	E2F3	GATA5	KNL1	MEOX1	MYL7	ORC1	PPP2R5C	SKP1	TFDP1	VLDLR
ADM	BUB1	CDC23	CKM	E2F4	GATA6	KRT14	MFN2	MYLK	ORC2	PPP2R5D	SKP2	TFDP2	WAPL
ADPRHL1	BUB1B	CDC25A	CKMT2	E2F5	GEM	KRT17	MITF	MYLPF	ORC3	PPP2R5E	SLC5A1	TGFB1	WASHC1
ALPK3	BUB3	CDC25B	CLOCK	EDN1	GFAP	KRT5	MME	MYOD1	ORC4	PRG4	SLC6A13	TGFB2	WEE1
ANAPC1	CA3	CDC25C	CMKLR1	EGFR	GJA5	LAMA2	MMP2	MYOG	ORC5	PRKCQ	SLN	TGFB3	WEE2
ANAPC10	CAPN1	CDC26	CNN1	EN1	GJB2	LAMB3	MMP3	MYOM1	ORC6	PRKDC	SMAD2	THBS4	WIF1
ANAPC11	CAPZA3	CDC27	COL13A1	ENO3	GPIHBP1	LAMC2	MMP9	MYOM2	PAMR1	PTGIR	SMAD3	TICRR	WIPF1
ANAPC13	CASQ2	CDC45	COL4A3	EP300	GRWD1	LDB1	MRC1	MYOZ1	PAX3	PTK2	SMAD4	TMOD4	YWHAB
ANAPC15	CAV3	CDC6	COL4A4	ESCO1	GSK3B	LDB3	MSTN	MYOZ2	PAX7	PTTG1	SMC1A	TNNC1	YWHAE
ANAPC16	CCL11	CDC7	COL7A1	ESCO2	HAMP	LIF	MTBP	NBR1	PCNA	PTTG2	SMC1B	TNNC2	YWHAG
ANAPC2	CCL19	CDCA5	CORO6	ESPL1	HAND1	LOX	MTTP	NDC80	PCSK6	PXN	SMC3	TNNI1	YWHAH
ANAPC4	CCL2	CDH11	CPT1A	FABP4	HAND2	LRRK1	MUSK	NEB	PDE1A	PYGM	SMIM3	TNNI2	YWHAQ
ANAPC5	CCNA1	CDH15	CREBBP	FAP	HAS1	MAD1L1	MYBPC3	NEXN	PDE4D	RAD21	SMPX	TNNI3	YWHAZ
ANAPC7	CCNA2	CDH3	CSRP3	FBLN7	HDAC1	MAD2L1	MYC	NFATC1	PDLIM5	RB1	SORBS2	TNNT1	ZBTB16
ANGPT2	CCNB1	CDK1	CUL1	FBXO5	HDAC2	MAD2L1BP	MYD88	NGF	PDS5A	RBL1	SOX18	TNNT2	ZBTB17
ANKRD1	CCNB2	CDK2	CXCL1	FGF2	HDAC8	MAD2L2	MYF5	NGFR	PDS5B	RBL2	SOX2	TNNT3	ZFPM2

Table 4: Genes used for analysis of the Reprogramming dataset.

filtering or preprocessing to this data and used the raw signals as is. These data were obtained from the PhysioNet Database Portal and accessible at `https://www.physionet.org/content/eegmmidb/1.0.0/`

5.5 FUCCI Microscopy

We obtained microscopy signals from [22] where the FUCCI assay and Hoechst stain were applied to track cells and monitor their progression through the cell cycle. The mCherry and TagGFP are FUCCI markers and Hoechst is used to track the nucleus of individual cells over time. From the time series images, the expression of each of these three markers were measured every 5 minutes based on their intensity in the microscopy images.

On the left of fig. 20, we show a hundred trajectories of these three markers in their 3D phase portrait. The discrete time sampled data were linearly interpolated. On the right of the figure, each of the three pairs of data are shown during different cell cycle phases. We used codes from [22] for cell cycle phase segmentation based upon the FUCCI system. Similar to fig. 1, the variability or information content of each of the three signals changes between cell cycle stage. For instance, TagGFP contains more variability in S/G2/M than in G1.

5.6 Reference Databases

We used the KEGG, HumanTF, PanglaoDB, databases as references to identify pathways known in the literature, define transcription factors, and find genes associated with myogenesis in our study.

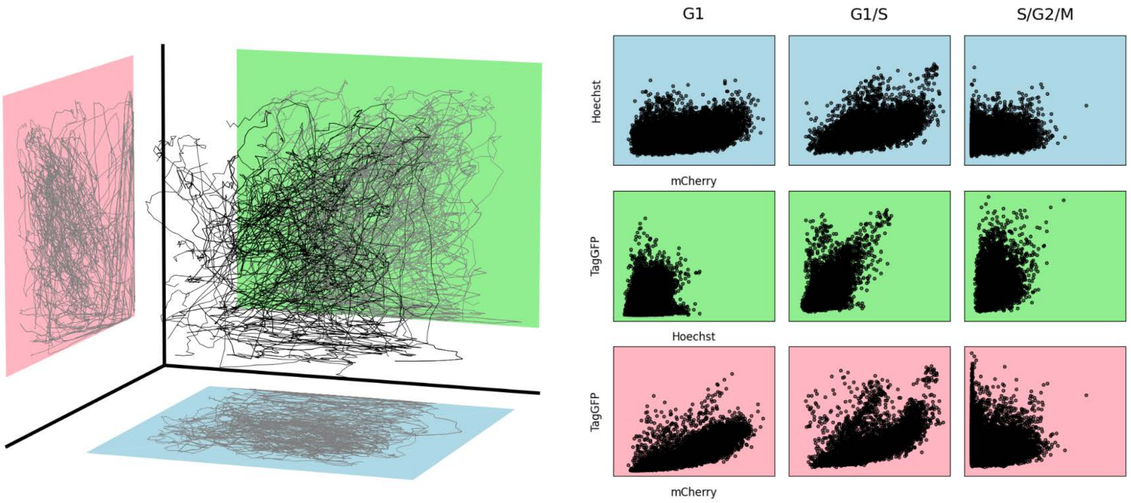

Figure 20: **FUCCI Microscopy**. (left) 3D phase portrate of the FUCCI system with Hoechst stains collected from time series data. (right) The same data of three pairwise observations of only 2 of the 3 signals, i.e. (1) Hoechset and mCherry, (2) TagGFP and Hoechst, and (3) TagGFP and mCherry, are shown and separated during the cell cycle stages.

5.6.1 KEGG

To identify known and biologically meaningful sensor genes, we utilized pathways from KEGG [55]. We downloaded and considered all human (HSA) pathways and used them as sensor genes to estimate the PROLIFERATION dataset. These data were from the February 2024 version of the database and accessed at `https://www.genome.jp/kegg-bin/show_organism?menu_type=pathway_maps&org=hsa`

5.6.2 HumanTF

To characterize genes and biomarkers as Transcription Factors, we utilized the Human Transcription Factors database (version 1.0.1), which contains 1639 genes as TFs [62]. The data were accessed at `http://humantfs.ccbr.utoronto.ca/`

5.6.3 PanglaoDB

To select genes for the MyogrenicSignal, we used marker genes for different myogenic cells and fibroblasts that were found in the PangloDB database [31]. These data were from the March 27, 2020 version of the database and can be found at `https://panglaodb.se/markers.html?cell_type=%27choose%27`

6 Supplementary Figures

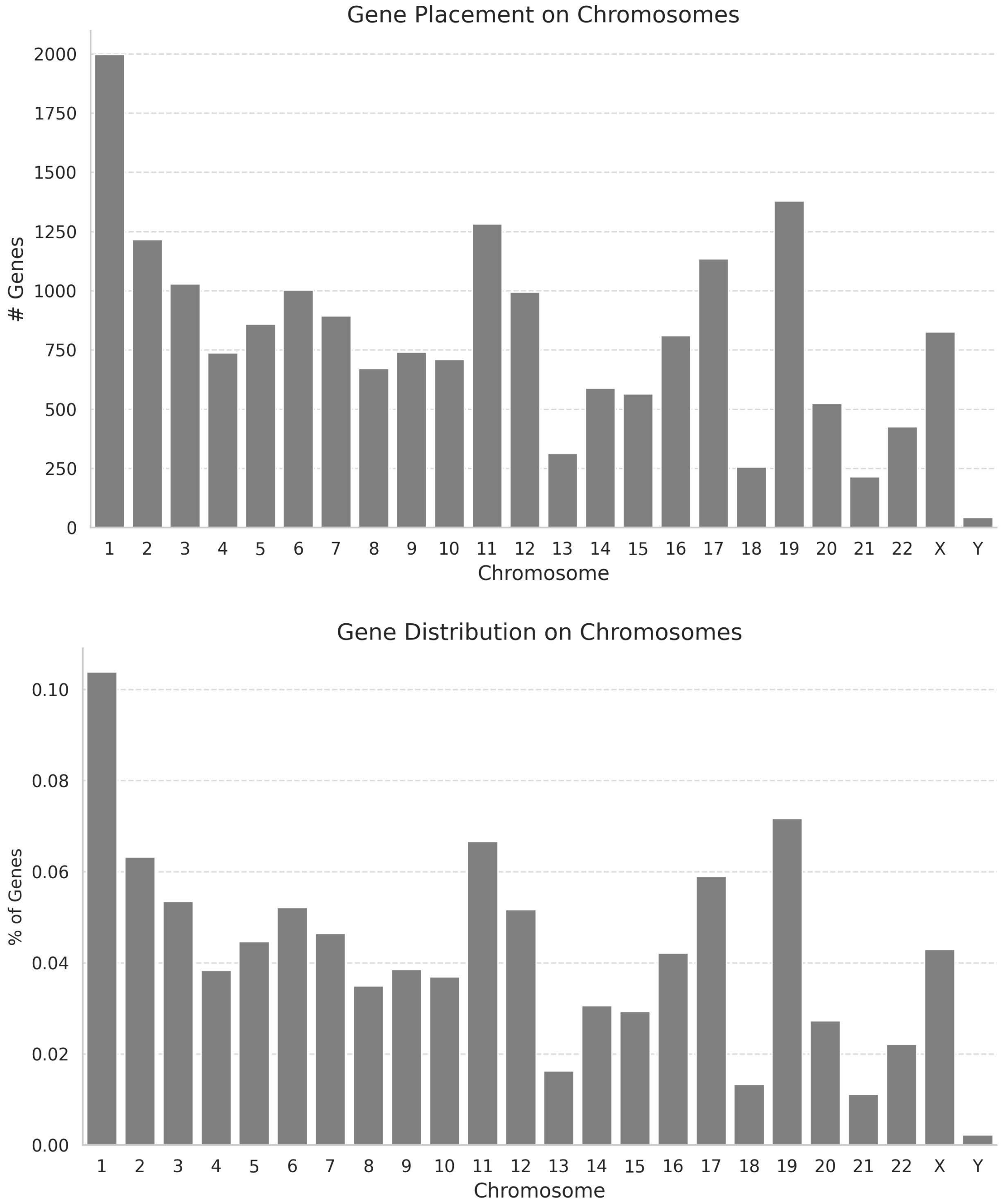

Figure 21: The distribution of genes across chromosomes is hardwired and contributes to the structure and spatial organization of gene regulatory networks.

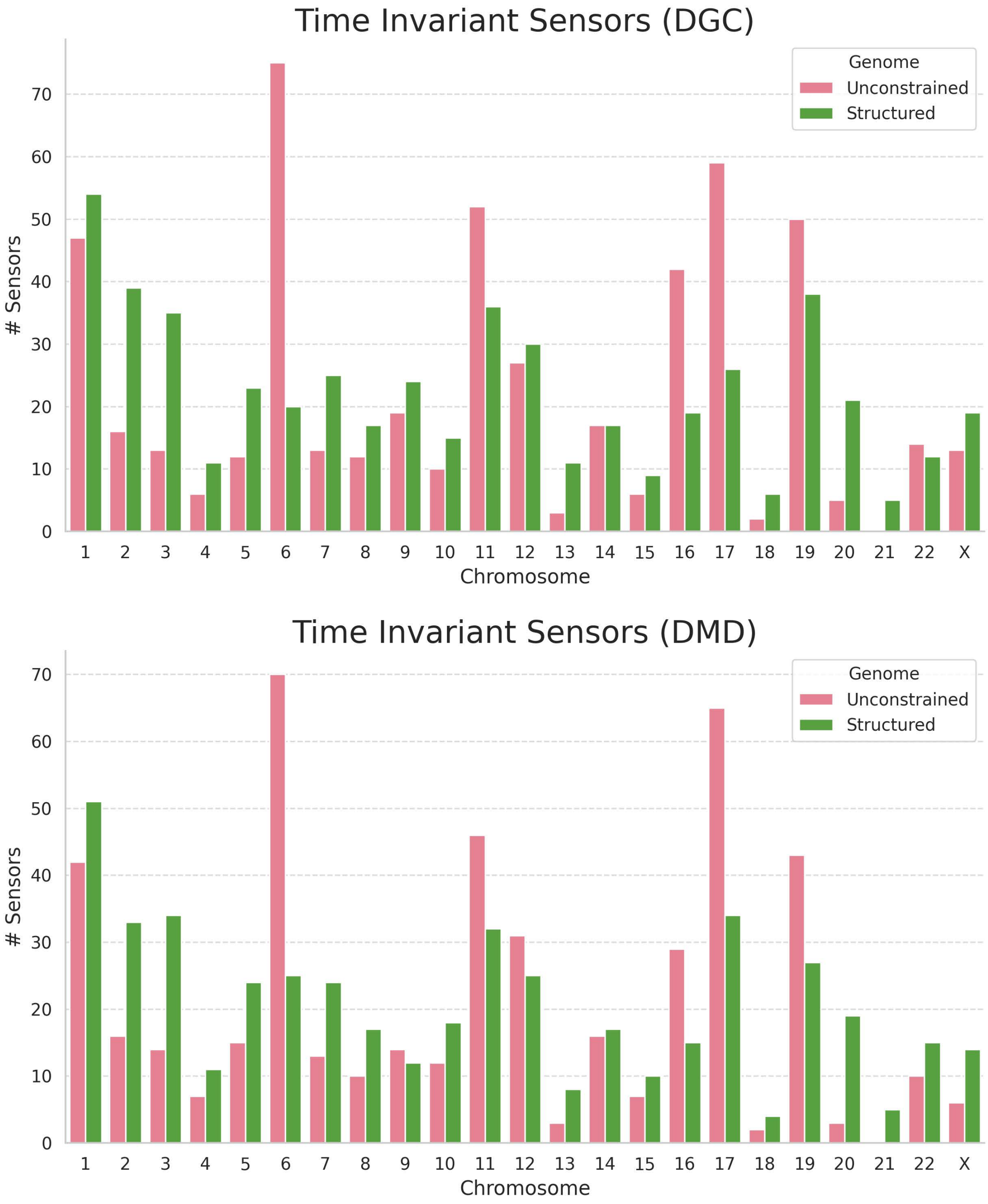

Figure 22: The placement of the top two thousand sensor genes across chromosomes is shown for the set difference of the selected sets.

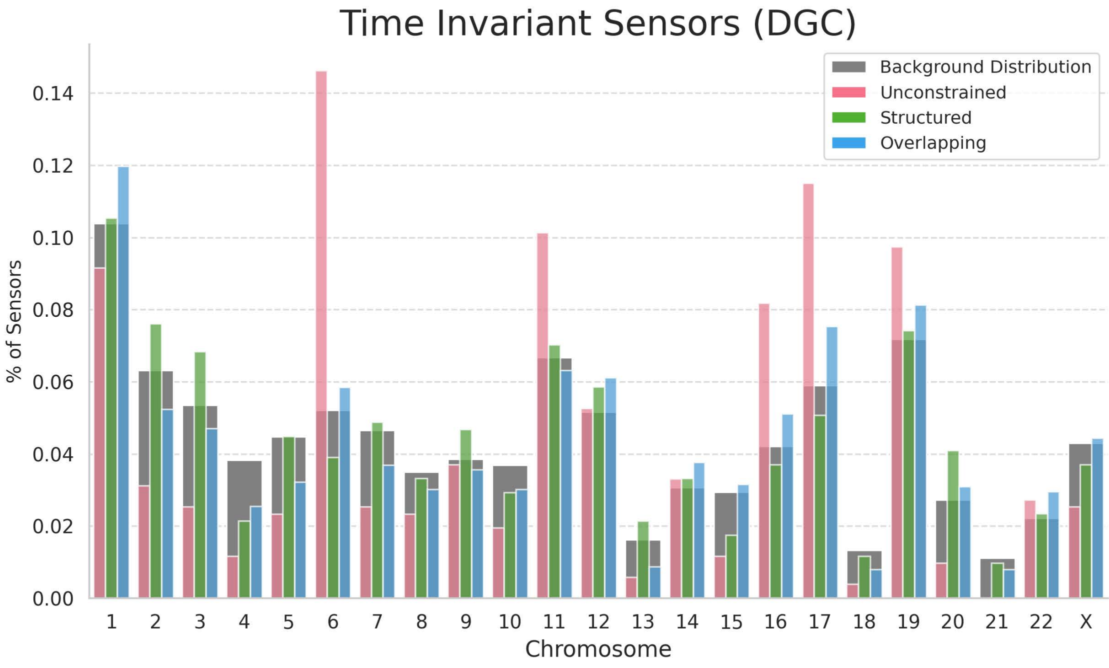
Time Invariant Sensors (DGC)
Background Distribution
Unconstrained
Structured
Overlapping
% of Sensors
0.00
0.02
0.04
0.06
0.08
0.10
0.12
0.14
1 2 3 4 5 6 7 8 9 10 11 12 13 14 15 16 17 18 19 20 21 22 X
Chromosome

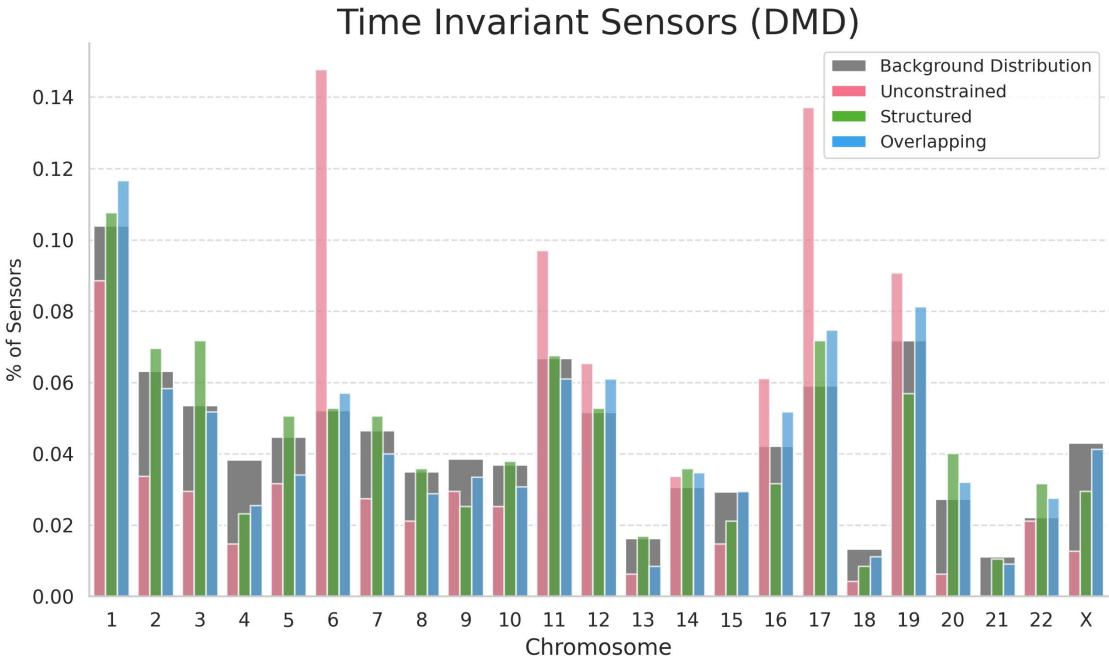
Time Invariant Sensors (DMD)
Background Distribution
Unconstrained
Structured
Overlapping
% of Sensors
0.00
0.02
0.04
0.06
0.08
0.10
0.12
0.14
1 2 3 4 5 6 7 8 9 10 11 12 13 14 15 16 17 18 19 20 21 22 X
Chromosome

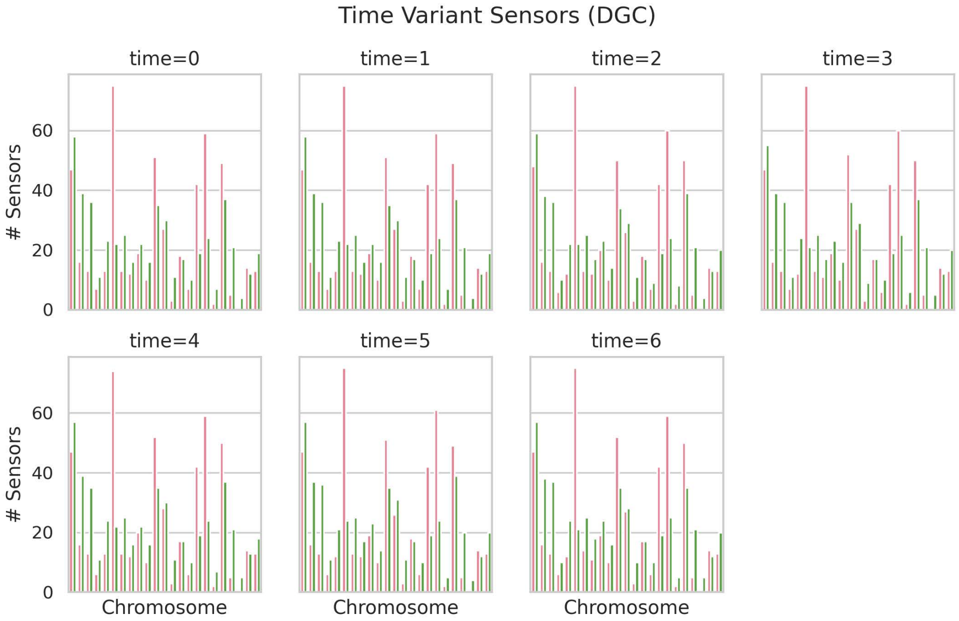
Time Variant Sensors (DGC)
time=0
time=1
time=2
time=3
time=4
time=5
time=6
# Sensors
0
20
40
60
Chromosome

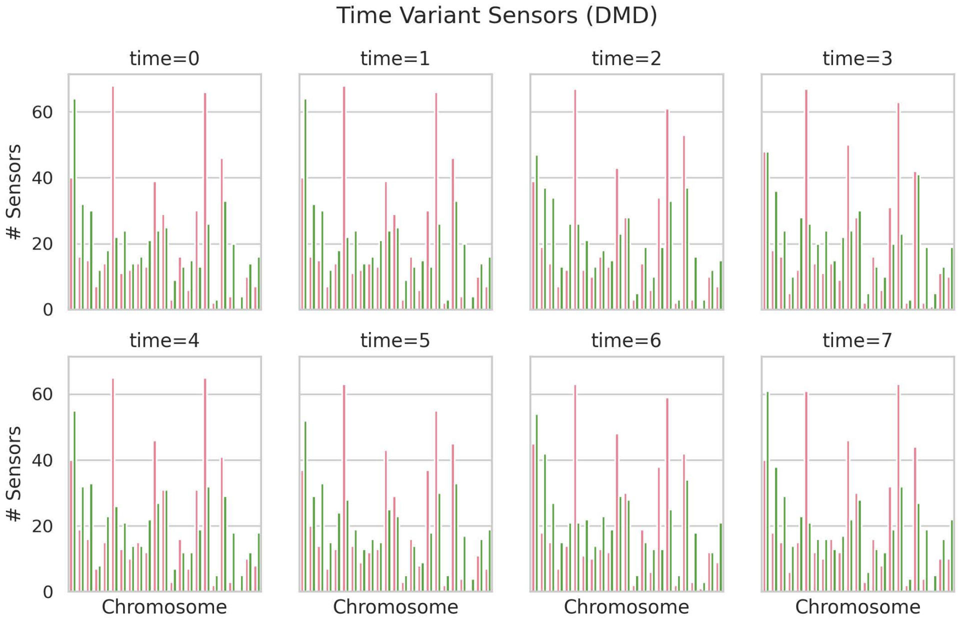
Time Variant Sensors (DMD)
time=0
time=1
time=2
time=3
time=4
time=5
time=6
time=7
# Sensors
0
20
40
60
Chromosome

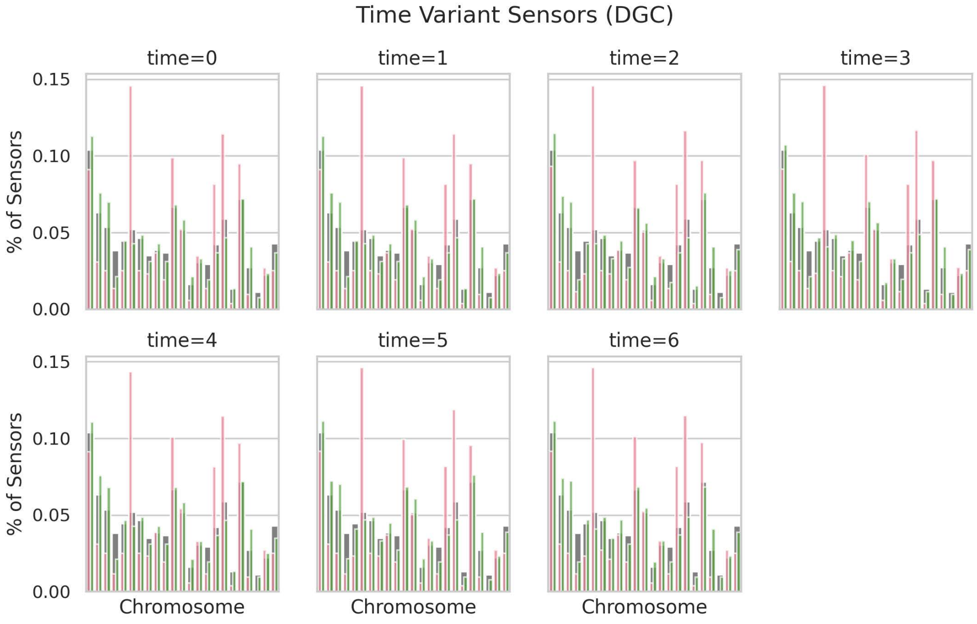

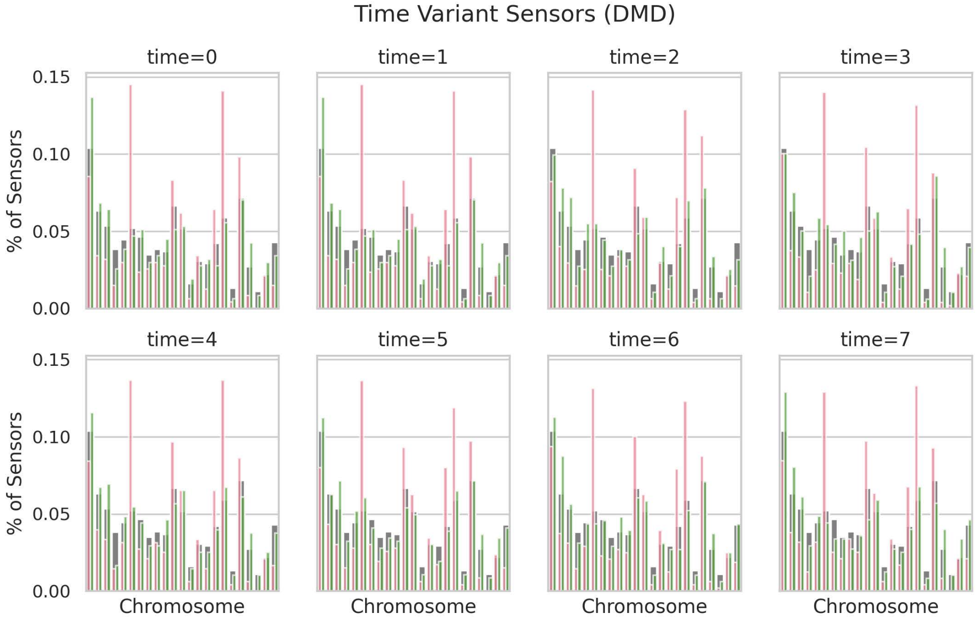

Figure 23: Final figure regarding gene sensor placement on different chromosomes.

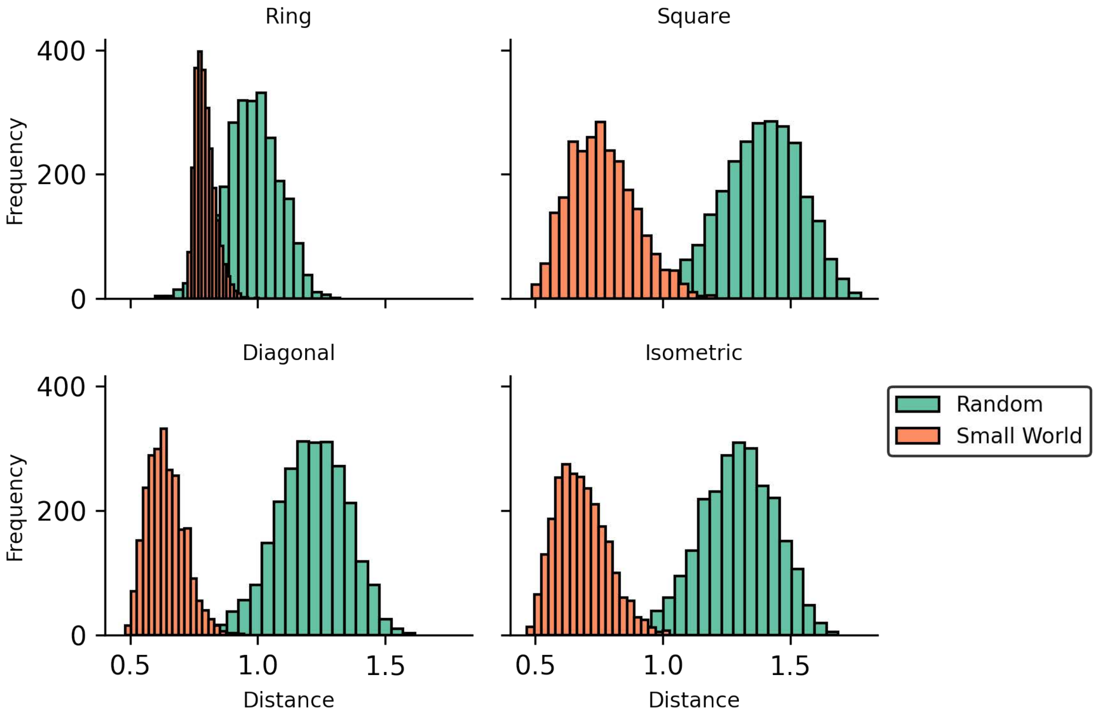

Figure 24: $\|\cdot\|_3$

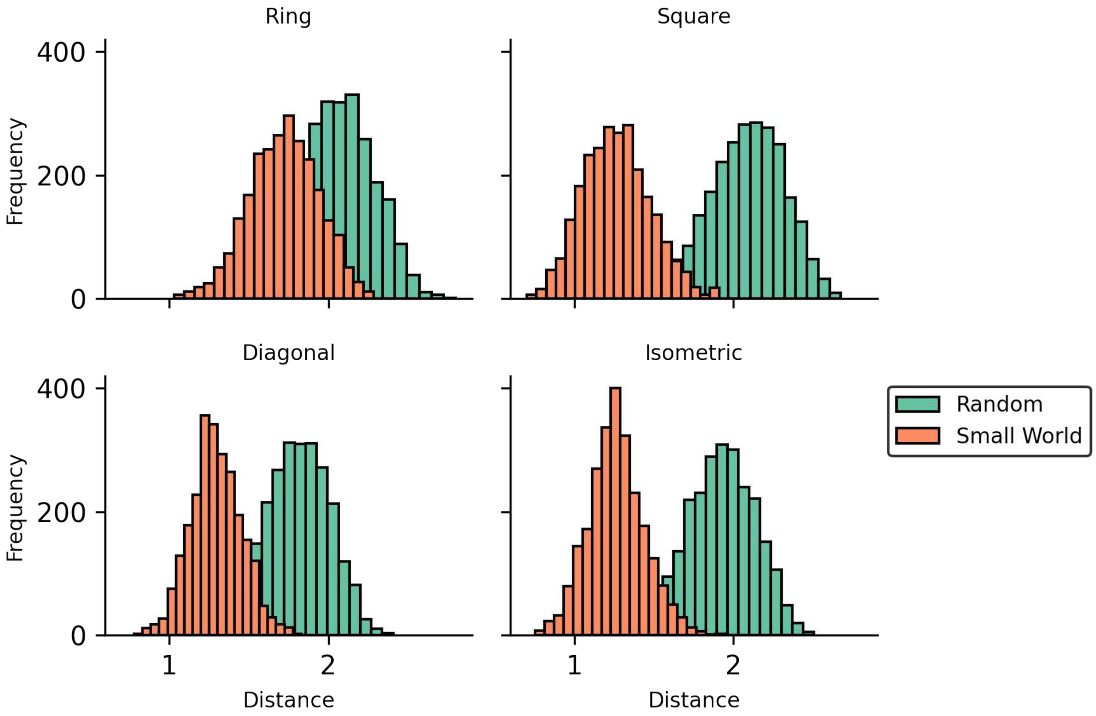

Figure 25: $\|\cdot\|_{10}$

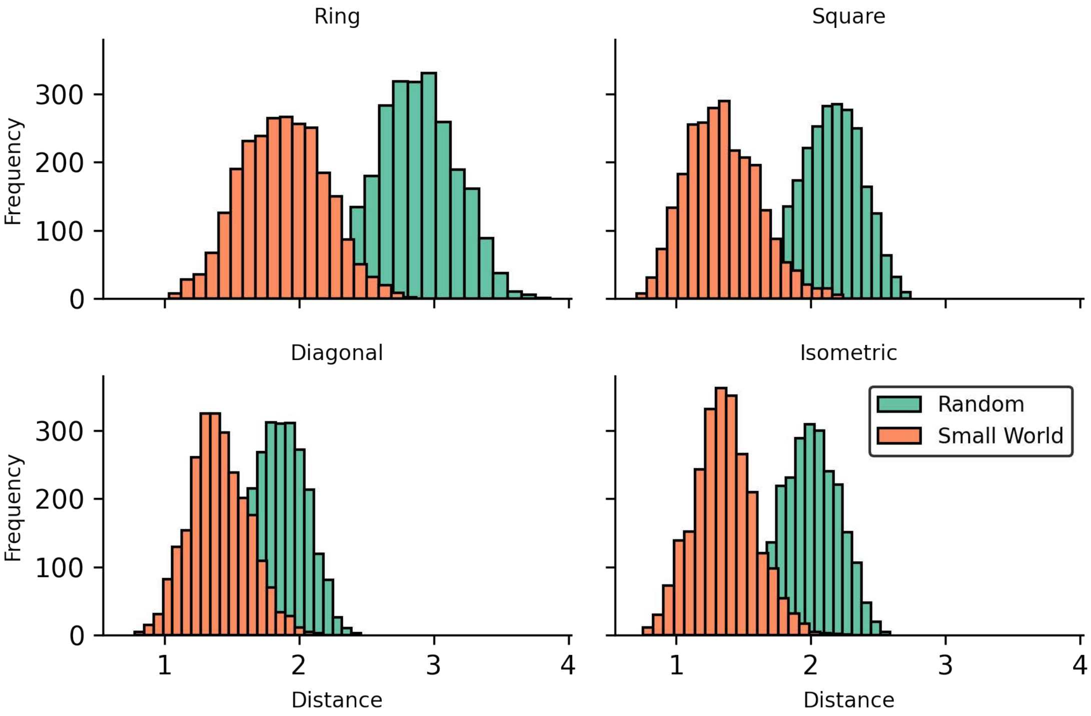

Figure 26: $\|\cdot\|_\infty$

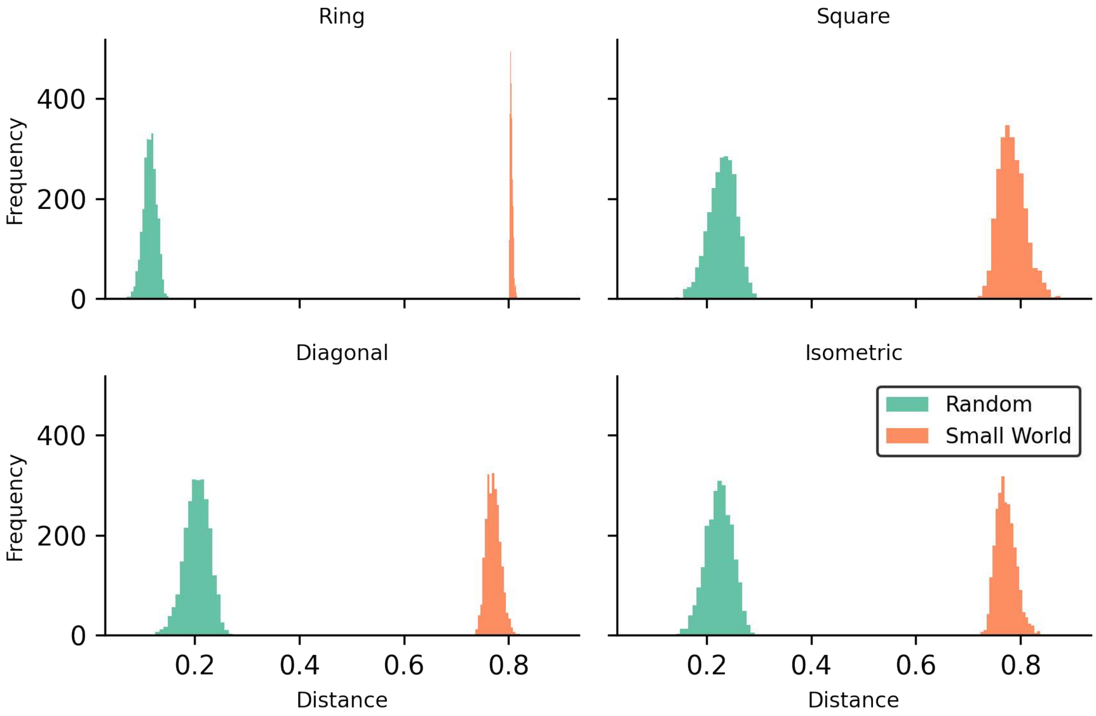

Figure 27: $\|\cdot\|_1$

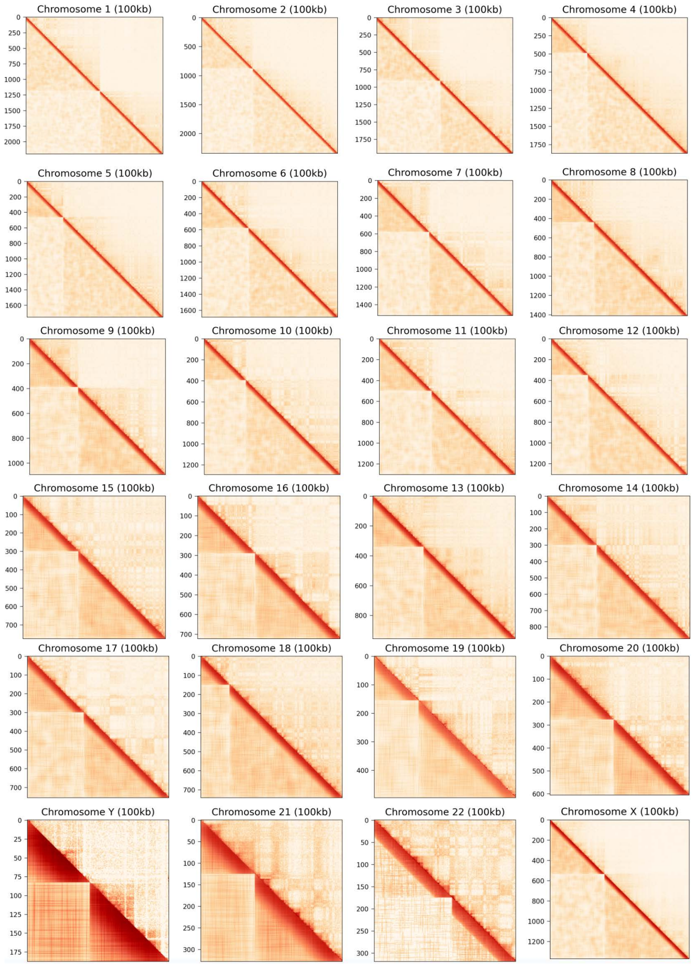

Figure 28: **Network Model and Hi-C.** This figure illstrates our simple network model applied to other chromosomes. It is interpreted similar to fig. 13.
